# A human CD137×PD-L1 bispecific antibody promotes anti-tumor immunity via context-dependent T cell costimulation and checkpoint blockade

**DOI:** 10.1038/s41467-021-24767-5

**Published:** 2021-07-21

**Authors:** Cecile Geuijen, Paul Tacken, Liang-Chuan Wang, Rinse Klooster, Pieter Fokko van Loo, Jing Zhou, Arpita Mondal, Yao-bin Liu, Arjen Kramer, Thomas Condamine, Alla Volgina, Linda J. A. Hendriks, Hans van der Maaden, Eric Rovers, Steef Engels, Floris Fransen, Renate den Blanken-Smit, Vanessa Zondag-van der Zande, Abdul Basmeleh, Willem Bartelink, Ashwini Kulkarni, Wilfred Marissen, Cheng-Yen Huang, Leslie Hall, Shane Harvey, Soyeon Kim, Marina Martinez, Shaun O’Brien, Edmund Moon, Steven Albelda, Chrysi Kanellopoulou, Shaun Stewart, Horacio Nastri, Alexander B. H. Bakker, Peggy Scherle, Ton Logtenberg, Gregory Hollis, John de Kruif, Reid Huber, Patrick A. Mayes, Mark Throsby

**Affiliations:** 1 Merus NV, Utrecht, The Netherlands; 2 Incyte Corporation, Wilmington, DE USA; 3 Perelman School of Medicine, University of Pennsylvania, Philadelphia, PA USA

**Keywords:** Cancer immunotherapy, Antibody therapy, Immunotherapy, Cancer immunotherapy

## Abstract

Immune checkpoint inhibitors demonstrate clinical activity in many tumor types, however, only a fraction of patients benefit. Combining CD137 agonists with these inhibitors increases anti-tumor activity preclinically, but attempts to translate these observations to the clinic have been hampered by systemic toxicity. Here we describe a human CD137xPD-L1 bispecific antibody, MCLA-145, identified through functional screening of agonist- and immune checkpoint inhibitor arm combinations. MCLA-145 potently activates T cells at sub-nanomolar concentrations, even under suppressive conditions, and enhances T cell priming, differentiation and memory recall responses. In vivo, MCLA-145 anti-tumor activity is superior to immune checkpoint inhibitor comparators and linked to recruitment and intra-tumor expansion of CD8 + T cells. No graft-versus-host-disease is observed in contrast to other antibodies inhibiting the PD-1 and PD-L1 pathway. Non-human primates treated with 100 mg/kg/week of MCLA-145 show no adverse effects. The conditional activation of CD137 signaling by MCLA-145, triggered by neighboring cells expressing >5000 copies of PD-L1, may provide both safety and potency advantages.

## Abstract (web summary)

The anti-tumour effect of immune checkpoint inhibitors is potentiated by CD137 agonists in preclinical models, but translation of these results to the clinical practice is hampered by toxicity. Authors describe here a human CD137xPD-L1 bispecific antibody with improved anti-cancer activity whilst maintaining low toxicity in non-human primates.

## Introduction

Cancer immunotherapy has made impressive strides in the last decade. A key factor behind this success is the progress made in effectively directing cytotoxic T cell responses against tumors via adoptive T cell therapy (with engineered T cells or expanded tumor-infiltrating lymphocytes) or immune checkpoint inhibitor (ICI) therapies^1^. Cytotoxic CD8+ T lymphocytes (CTLs) provide long-term protection against pathogens^2^ and carry out immune surveillance to eliminate tumor cells^3^. Tumor-specific CTLs are generated by the presentation of tumor-associated antigens (TAAs), derived from mutations and epigenetic changes on antigen-presenting cells (APC), driving tumor-specific T cell expansion, effector cell differentiation, and memory cell generation^4^. The extent and nature of CD8^+^ T cell activation is strongly influenced by the quality of T cell receptor (TCR)/peptide MHC interaction (signal 1) and the presence, type and strength of signaling through costimulatory receptors (signal 2); lack of appropriate costimulation has been shown to impact effective anti-tumor immune responses^5^.

Tumors exploit various mechanisms to evade immune destruction. One key barrier to effective anti-tumor immunity is immune checkpoints as exemplified by the PD-1/PD-L1 axis^6^. PD-1 is an inhibitory receptor expressed on activated T cells as well as other immune cells, and its expression is associated with T cell activation and subsequent exhaustion^7^. PD-1 binds to two ligands, PD-L1 (B7-H1) and PD-L2 (B7-DC), that are primarily expressed on dendritic cells (DCs) and myeloid lineage cells but can also be expressed on tumors, particularly in the case of PD-L1^8^. Furthermore, PD-L1 expression can be increased in the tumor microenvironment by IFNγ, a cytokine released by activated T cells^9^. Such PD-1/PD-L1 interactions deliver a negative signal to T cells dampening anti-tumor responses. This immune suppression can be blocked by ICIs such as antagonistic antibodies against PD-1 or PD-L1^8^. ICI treatment has demonstrated remarkably durable responses in a subset of cancer patients that are correlated with activated CD8+ T cell infiltration and proliferation^10,11^ Combinations of ICIs (e.g., anti-PD-1 and anti-CTLA-4) have been shown to further enhance efficacy, however at the cost of toxicity, as the majority of patients experience grade 3 or 4 treatment-related adverse events^12^. Strategies that add to the benefit of ICI therapies without further increasing toxicity are needed.

CD137 (4-1BB, tumor necrosis factor receptor superfamily 9) is an inducible costimulatory receptor expressed on activated T and natural killer (NK) cells. CD137 signaling is triggered by receptor clustering through interaction with its trimeric ligand (tumor necrosis factor superfamily 9), expressed on professional APCs, activating the NF-κB and MAPKs pathways^13^. On T cells, CD137 is transiently expressed after TCR engagement and once activated provides CD28-independent costimulation resulting in enhanced cytokine production, proliferation, survival, effector function, and immunological memory formation^14^. In the tumor microenvironment, CD137 is a marker for tumor-specific CTLs and is co-expressed with PD-1^15,16^. A body of orthogonal evidence suggests that the activity of tumor-specific CTLs can be enhanced by CD137 activation. Ectopic expression of CD137 ligand or an agonist antibody fragment on the surface of tumors has been shown to result in tumor elimination^17,18^. CD137 agonist antibodies drive CD137 clustering to induce signaling, analogous to the trimeric CD137 ligand, and demonstrate potent anti-tumor responses in preclinical models^19–24^. CD137 activation has been shown to upregulate PD-1 expression on effector T cells and PD-L1 on tumors via release of IFNγ and is linked to therapeutic resistance^22,23^. The combination of PD-1/PD-L1 pathway blocking antibodies with CD137 agonist antibodies in these models synergistically enhances anti-tumor responses^19–23^. Given the preclinical data, along with the high frequency of PD-1 and CD137 co-expression on tumor-specific CD8^+^ tumor-infiltrating lymphocytes (TILs) found in humans, there is a clear mechanistic rationale for dual targeting of the PD-1/PD-L1 axis and CD137 to optimally engage anti-tumor immunity.

The two most advanced therapeutic CD137 agonist antibodies in clinical testing are urelumab (IgG4) and utomilumab (IgG2). Utomilumab, in contrast to urelumab, requires Fc-mediated crosslinking to activate CD137 and shows weaker agonist activity^25,26^. Development of urelumab has been halted as a consequence of dose-dependent hepatitis resulting from systemic activation of the CD137 pathway in patients^27^. Safe administration of urelumab required a reduced dose of 0.1 mg/kg, which was chosen for combination studies with PD-1 inhibitors^27^. Utomilumab is better tolerated by patients but has modest anti-tumor activity as a monotherapy^28^ and no clear synergy with PD-1 blockade in combination therapy^29^. To address the shortcomings of current therapeutic strategies targeting CD137, we undertook an unbiased functional screening approach that identified a full-length bispecific antibody (bAb) that potently activates CD137 via engagement of PD-L1 to enhance tumor-specific T cell responses.

## Results

### Identification of MCLA-145 using unbiased functional screening

**Fig. 1:**
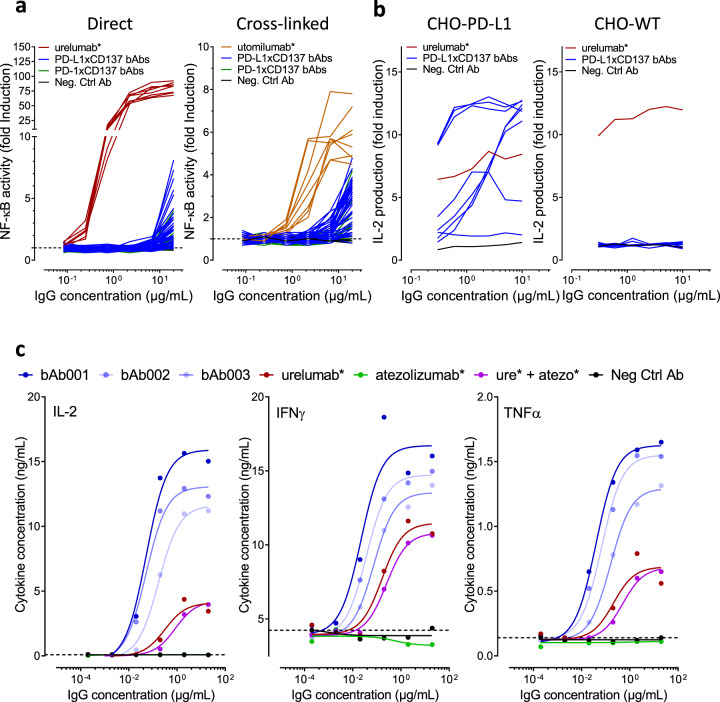
Screening of CD137 x PD-1/PD-L1 bAb combinations identifies MCLA-145. **a** Left panel activity of CD137xPD-L1 and CD137xPD-1 bispecifics in a CD137 NFκB-luc reporter assay (fold induction of luciferase signal) Right panel experiment repeated with the addition of an Fc binding antibody; **b** Left panel levels of IL-2 released by activated T cells after 3 days of incubation with eight candidate CD137xPD-L1 bAb combinations in a dose titration in the presence of CHO-PD-L1 cells, Right panel, experiment repeated in the presence wildtype CHO cells; **c** levels of IL-2, IFNγ and TNFα released by activated T cells after 3 days of incubation with the three best performing CD137xPD-L1 bAbs and control antibodies in a dose titration in the presence of CHO-PD-L1 cells (bAb001 = MCLA-145).

To produce dual T cell agonist and checkpoint blocking bispecific IgG antibodies, diverse common light chain (cLC) Fab panels against CD137, PD-1 and PD-L1 were generated. The Fab panels were reformatted into human immunoglobulin G1 (IgG1) and extensively characterized (Supplementary Table 1). A total of ~440 bAbs were produced using 47 different CD137 Fab arms (representing 11 epitope bins) combined with high affinity and functionally active PD-L1 [8×] or PD-1 [3×] Fab arms (Supplementary Table 1). The bAbs were expressed as IgG1 molecules with Fc CH3 modifications to force heavy chain heterodimerization^30^ and CH2 modifications to abrogate binding to Fc receptors^31^. Initial screening assays showed the CD137xPD-L1 and CD137xPD-1 bAbs weakly induced luciferase expression in anti-CD3-activated Jurkat NFκB−luc CD137 reporter cells (Fig. 1a), despite the fact that the CD137 reporter cells upon anti-CD3 treatment co-expressed CD137 with PD-1 and PD-L1 (Supplementary Fig. 1a). The addition of an anti-human IgG to crosslink CD137 antibodies did not meaningfully increase activation of CD137 reporter cells (Fig. 1a). Our data demonstrate that dual engagement of CD137 and PD-1 or PD-L1 by bAbs either on the same T cell (in cis) or possibly on adjacent T cells (in trans) was not able to effectively induce CD137 receptor downstream signaling.

When CHO cells expressing human PD-L1 were co-cultured with CD137 reporter cells under the same assay conditions, we observed several CD137xPD-L1 bAbs able to induce activation of CD137 reporter cells. We then performed serial titrations of a panel of CD137xPD-L1 bAbs in a human primary T cell assay containing WT or PD-L1 expressing CHO cells (Fig. 1b). Urelumab* potently activated T cells as measured by IL-2 secretion with or without PD-L1 expression on the co-cultured CHO cells. The CD137xPD-L1 bAb panel demonstrated a range of potency, some comparable to urelumab*, but only in the presence of PD-L1 antigen on the co-cultured CHO cells. The T cell agonist activity of the CD137xPD-L1 bAb panel was confirmed in a transactivation assay using purified T cells and staphylococcal enterotoxin B (SEB) activated PBMCs (Supplementary Fig. 1b). The most potent CD137xPD-L1 bAbs shown in (Fig. 1c) led to greater cytokine release than urelumab* or the combination of urelumab* and atezolizumab*. In the SEB assay, the polyclonal stimulation of T cells is also associated with the induction of high levels of PD-L1 expression on a subset of CD14+ cells and CD137 induction on CD8^+^ T cells (Supplementary Fig. 1c) demonstrating that effective costimulation can be achieved under inflammatory conditions. MCLA-145 was selected from the CD137xPD-L1 bAbs panel based on its potent function and optimal biophysical characteristics to facilitate IgG manufacturing.

### MCLA-145 exhibits high affinity for its targets and binds a distinct epitope on CD137

**Fig. 2:**
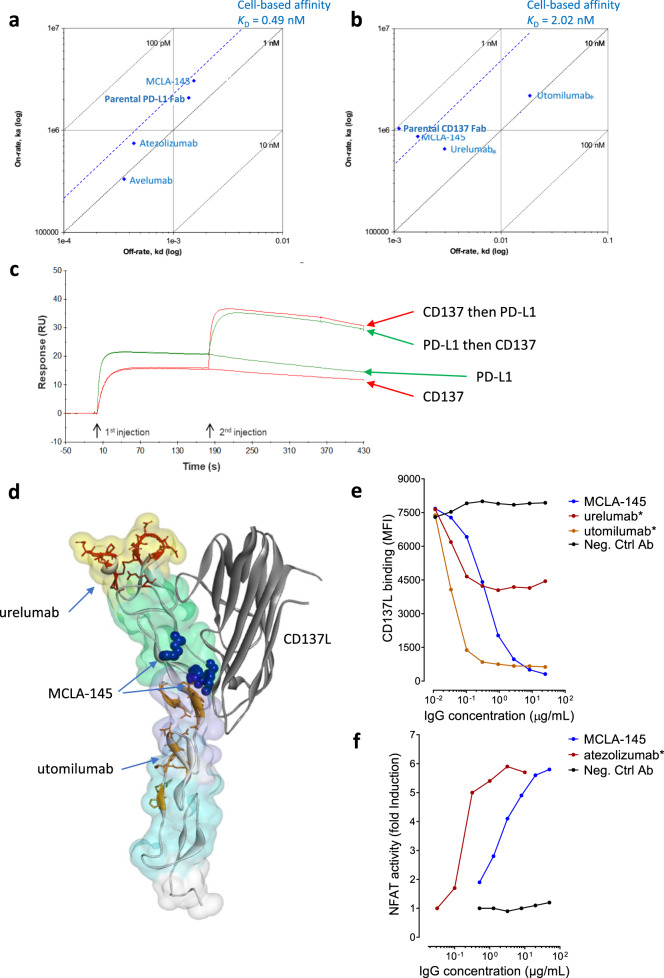
MCLA-145 binds with high affinity and to a unique epitope on CD137. **a** Summary of SPR and cell-based affinity measurements for binding to PD-L1; **b** Summary of SPR and cell-based affinity measurements for binding to CD137; **c** SPR affinity analysis showing dual binding of human PD-L1 and CD137 to immobilized MCLA-145. Green curves: human PD-L1 injected first followed by injection of human CD137 or running buffer. Red curves: human CD137 injected followed by human PD-L1 or running buffer; **d** surface homology model of the CD137 ectodomain in complex with CD137L (PDB: 6MGP), the CRD domains 1–4 are shaded in yellow, green, purple and cyan respectively, CD137L is depicted as a gray ribbon, the backbone and residues of urelumab* (dark red) and utomilumab* (orange) epitopes are highlighted, critical binding residues for MCLA-145 as determined by alanine scanning are shown as solid blue spheres; **e** binding of labeled CD137L to CHO-CD137^+^ cells was measured by flow analysis in a titration series of MCLA-145 and control antibodies **f** NFAT activity of PD-1 reporter cells cocultured with CHO-PD-L1^+^ and incubated with a titration of MCLA-145 and control antibodies.

MCLA-145 binds with high affinity to CHO cells expressing either human CD137 or PD-L1 and to activated human T cells as determined by flow cytometry (Supplementary Fig. 2a). The affinity of MCLA-145 for its targets was assessed by surface plasmon resonance (SPR) with recombinant proteins and confirmed in a cell-based system where the targets are in their native conformation (Fig. 2a, b). By SPR, MCLA-145 was shown to bind PD-L1 (0.51 ± 0.05 nM) and CD137 (1.9 ± 0.2 nM) with high affinity (Supplementary Table 2). MCLA-145 was capable of binding to both targets simultaneously on an SPR sensor (Fig. 2c). The cell-based affinity of MCLA-145 for human PD-L1 and human CD137 in native conformation was confirmed by Scatchard analysis (0.49 ± 0.03 nM and 2.02 ± 0.02 nM respectively) using radiolabeled antibody (Supplementary Table 3). The affinity and binding kinetics of MCLA-145 are in the same range as comparator antibodies atezolizumab* and avelumab* for PD-L1 (Fig. 2a) and urelumab* and utomilumab* for CD137 (Fig. 2b).

The binding location of CD137 mAbs strongly influences their functional properties^26^. The epitope of MCLA-145 on CD137 was first explored using a domain swap approach, exploiting the observation that MCLA-145 binds with low affinity to murine CD137. MCLA-145 was only capable of binding with high affinity to HEK293T cells transiently expressing constructs that retained the human cysteine-rich domain (CRD) 2 (Supplementary Fig. 2b). Using a separate mapping approach, critical interaction sites of MCLA-145 on CD137 were mapped through an alanine scanning experiment to residues R66, G70, V71, and F72 located in CRD2 (Fig. 2d, Supplementary Fig. 2c). Alanine replacement at C133 reduced MCLA-145 binding but this effect was considered due conformational alterations rather than directly interfering with MCLA-145 binding. Collectively the mapping data show that the MCLA-145 epitope on CD137 resides in CRD2, a region critical for CD137L interaction^32^ but distinct from the binding epitopes of utomilumab and urelumab (Fig. 2d). The CD137 epitope mapping is consistent with the functional characteristics of the CD137 binding arm of MCLA-145 which is not agonistic without crosslinking and is able to block CD137L binding to CD137 (Fig. 2e). It is noteworthy that these characteristics were shared by the most potent CD137xPD-L1 bAbs in the panel (indicated in bold in Supplementary Table 1) suggesting this binding region to be optimal for trans-activation of CD137 by bAbs, at least in the IgG1 format.

The PD-L1 binding arm of MCLA-145 plays a dual role; enabling context-dependent activation of CD137 while also blocking interaction with PD-1 on T-cells. The monovalent anti-PD-L1 activity of MCLA-145 was evaluated in a PD-1 reporter assay and compared to the bivalent monoclonal antibody atezolizumab* (Fig. 2f). Although we measured the monovalent affinity of MCLA-145 for PD-L1 to be equivalent to atezolizumab* (Fig. 2a) the ED_50_ of atezolizumab* was significantly lower than MCLA-145 in the reporter assay. The maximal PD-1 inhibition reached by MCLA-145 was similar to atezolizumab* and at concentrations comparable to those maximally activating CD137 on T cells in vitro. Collectively, these data suggest that MCLA-145 could intensify antigen-specific T cell responses in the tumor micro-environment by simultaneously blocking the inhibition of TCR signaling by PD-1 in addition to providing costimulation through CD137 signaling.

### MCLA-145 potently activates T cells, promotes immunological memory, and overcomes immunosuppression

**Fig. 3:**
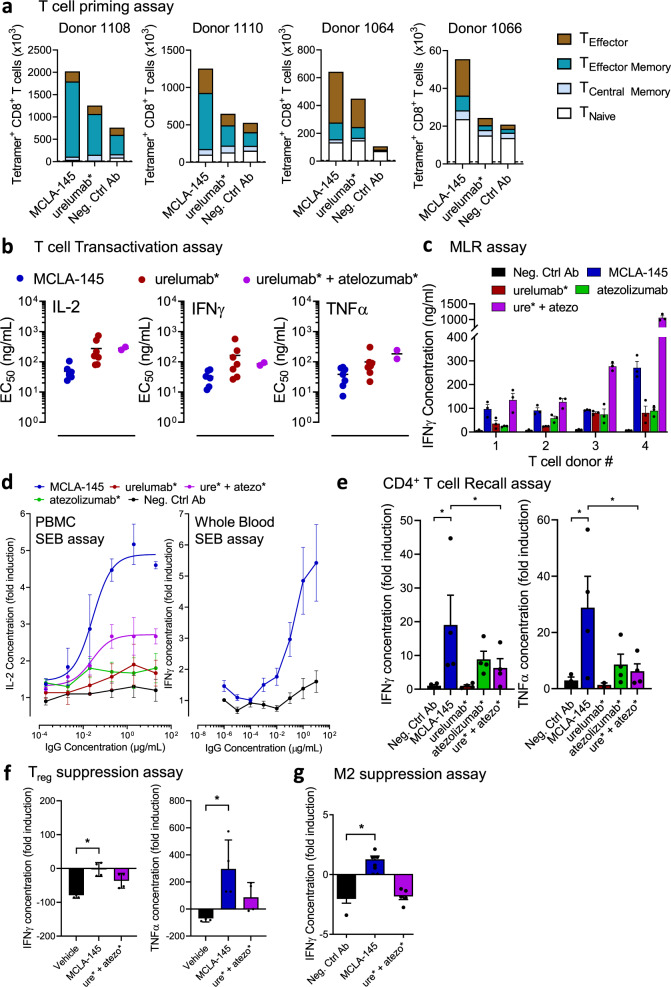
Effects of MCLA-145 activity on primary T cell function. **a** Effect of MCLA-145 on naïve CD8^+^ T cell priming. Graphs show the absolute number of dextramer+ antigen-specific CD8^+^ T cells from four donors after 10 days of co-culture with colored segments indicating the number of T naïve/memory stem cells (CD45RA + CCR7+), central memory T cells (CD45RA-CCR7+), effector memory T cells (CD45RA-CCR7-) and terminally differentiated effector T cells (CD45RA + CCR7−) as determined by flow cytometry (Dotted line represents control co-cultures without peptide priming). **b** Transactivation assay. EC_50_ of human T cell cytokine production (IL-2, IFNγ, and TNFα) in the presence of CHO-PD-L1 cells (*n* = 7); **c** mixed lymphocyte reaction (MLR) measuring IFNγ production by human donor leukocytes (*n* = 3 independent donors) stimulated with allogeneic DCs (*n* = 1) in the presence of 10 µg/ml of test compounds, (error bars are SEM), IFNγ concentrations were significantly greater for MCLA-145 compared to urelumab*, atezolizumab* and Neg. Ctrl Ab (*p* < 0.001) and significantly less compared to ure* + atezo* (*p* < 0001) as determined by two-way ANOVA and Holm-Sidak’s multiple comparisons test; **d** SEB assays. Fold change in IL-2 or IFNγ production by PBMC (left panel, *n* = 3 independent donors, error bars are mean ± SD) or Whole blood (right panel, *n* = 7 independent donors, error bars are SEM) respectively after stimulation with SEB in the presence of increasing concentrations of antibodies; **e** Recall assay measuring fold induction of IFNγ and TNFα production by human CD4+ memory T cells re-stimulated with a viral peptide pool (CEFT) in the presence of 10 µg/ml antibodies, (*n* = 4 independent donors, error bars are SEM), *p < 0.0001 for IFNγ and *p* = 0.0012 for TNFα production (Neg. Ctrl Ab vs. MCLA-145) and *p* = 0.0026 for IFNγ and *p* = 0.0025 for TNFα production (MCLA-145 vs. ure* + atezo*) as determined by two-way ANOVA and Tukey’s test; **f** Treg assay measuring fold induction of TNFα and IFNγ production by activated leukocytes in the presence of autologous regulatory T cells (1:1 ratio) and in the presence of 10 µg/ml antibodies (*n* = 4 independent donors error bars are SD of the mean). *p = 0.0005 for IFNγ and *p* = 0.012 for TNFα production (Vehicle vs MCLA-145), as determined by one-way ANOVA and Tukey’s test; **g** PBMC:M2 suppression assay measuring IFNγ production by human leukocytes co-cultured with activated M2-polarized macrophages together with 10 µg/mL antibodies (*n* = 5 independent donors, error bars are SEM, *p < 0.0001 by one-way ANOVA and Tukey’s test).

Antigen naïve T cells that encounter their cognate antigen will transiently express CD137^14^. We assessed the effects mediated by MCLA-145 on the priming of human naïve CD8+ T cells in an antigen-specific priming assay with peptide-pulsed human DCs expressing PD-L1. MCLA-145 significantly expanded the absolute number of antigen-specific CD8^+^ T cells (MCLA-145 compared to Neg. Ctrl Ab, *p* < 0.01, as determined by one-way ANOVA and Tukey’s multiple comparisons test) and preferentially enhanced the proportion of antigen-specific T cells with an effector and memory phenotype (Fig. 3a and Supplementary Fig. 3a). The absolute number of antigen-specific T cells was greater after MCLA-145 treatment compared to urelumab* while the proportion of T cell populations was more donor than treatment related. These results are consistent with studies in mouse models that show the engagement of CD137 during T cell priming is a critical determinant of secondary immune responses and clearly linked to the expansion of the antigen-specific memory CD8+ T cell pool^33^. The effect on T cell expansion or the proportion of differentiated T cells was unaltered when atezolizumab* was combined with urelumab* nor did atezolizumab* alone have activity (Supplementary Fig. 3b). Experiments with PD-1 and PD-L1 blocking antibodies confirmed that T cell expansion and differentiation was independent of the PD-1 signaling axis (Supplementary Fig. 3c). Taken together these observations indicate the potent enhancement of T cell priming by MCLA-145 is mediated by CD137 activation and not PD-1 axis blockade.

To assess the potency of MCLA-145 on pre-activated T cells, we used a battery of orthogonal in vitro assays. In a transactivation assay using purified T cells collected from 7 different donors, EC50 values for MCLA-145 ranged from 10 to 117 ng/mL and for urelumab* from 22 to 1752 ng/mL for the various cytokines (Fig. 3b). MCLA-145 also demonstrated potent activity in a mixed lymphocyte reaction (MLR) using allogenic human DCs (Fig. 3c) and a SEB assay using PBMCs or whole blood (Fig. 3d). In contrast to CD8^+^ T cells, there is no consensus on the role CD137 signaling has on CD4^+^ T cells^34^. MCLA-145 was incubated with human CD4^+^ memory T cells re-stimulated with their cognate antigen to investigate its activity in this population. MCLA-145 treatment enhanced IFNγ and TNFα release, whereas urelumab* had no effect. Atezolizumab* treatment induced an increase in IFNγ and TNFα levels that could not be increased further by the addition of urelumab* (Fig. 3e). This suggests the effect of MCLA-145 in the CD4^+^ T cell recall response was mainly due to inhibition of the PD-1 pathway.

Tumors in patients that do not respond to checkpoint blockade are often infiltrated by large numbers of suppressive immune cells, in particular T regulatory cells (Treg) and tumor-associated macrophages (TAM) that may dampen immune responses^35,36^. To test whether MCLA-145 could overcome the suppressive effect of these cell types, T cell activation assays were conducted in the presence of Tregs or suppressive M2 macrophages (Fig. 3f, g). The addition of Tregs was able to suppress T cell-dependent IFNγ and TNFα cytokine secretion by 79% and 69%, respectively. The addition of MCLA-145 was able to fully overcome the inhibition in cytokine secretion (Fig. 3f). In contrast, the combination of atezolizumab* and urelumab* partly overcame the Treg-induced inhibition of cytokine secretion. Similarly, the addition of MCLA-145 to PBMC:M2 macrophage co-cultures was able to overcome the immune suppression induced by M2 macrophages, as measured by IFNγ production, while the negative control antibody or the urelumab* and atezolizumab* combination could not (Fig. 3g). Together these results point to the multifunctional role MCLA-145 can play in an immune-suppressive environment.

### MCLA-145 recruits and activates T cells ex vivo and in vivo to enhance antitumor immunity

**Fig. 4:**
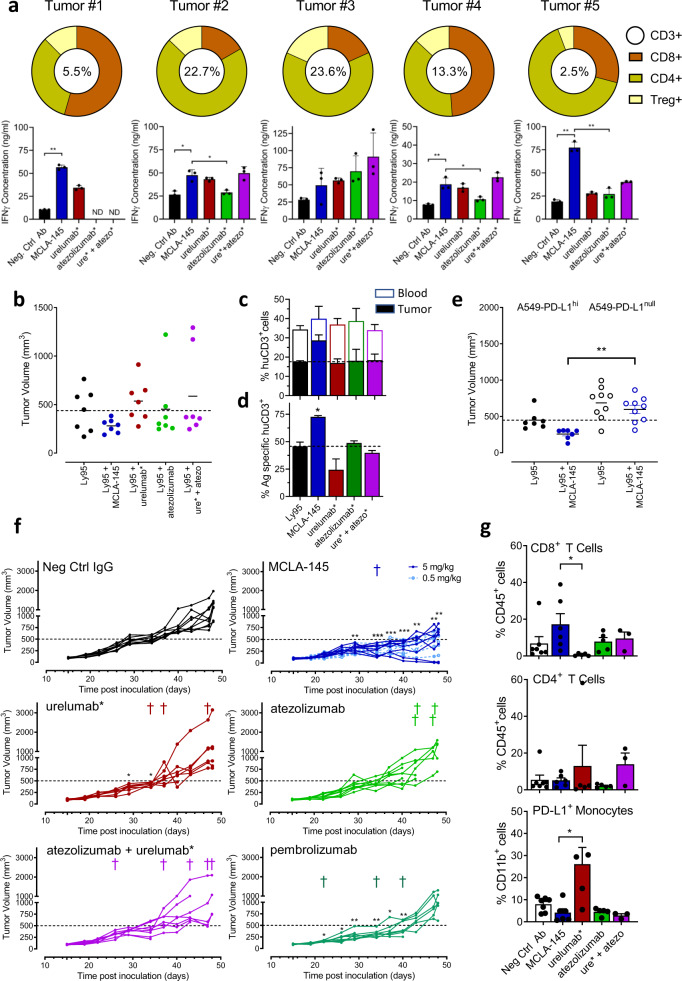
Ex vivo and in vivo activity of MCLA-145. **a** Top panel, donut chart of relative proportion of T cell subsets including percentage of CD3+ cells in single-cell suspensions derived from five human endometrial tumors; Bottom panel, IFNγ levels in supernatant of each tumor explant 6 days after antibody incubation. Error bars: mean ± SD of three replicates, ND = not done, **p < 0.0001 in Tumor #1 and Tumor #5, *p = 0.0018 (Neg. Ctrl Ab vs. MCLA-145) and *p* = 0.0042 (MCLA-145 vs. atezolizumab*); Tumor #2, **p = 0.0007 (Neg. Ctrl Ab vs. MCLA-145); Tumor #4 *p = 0.0074 (MCLA-145 vs. atezolizumab*), determined by one-way ANOVA and Tukey’s test; **b** Ly95 T cells expressing an NY-ESO-specific TCR were transferred to NSG mice bearing A549-PD-L1^hi^ tumors and treated with indicated antibodies, end point data are shown, (*n* = 7 mice); **c** Stacked histogram, percentage of human CD3^+^ lymphocytes in total live cell population in blood after RBC lysis (open bar) and tumor (solid colored bar) in each of the treatment groups in (**b**); **d** Proportion of NY-ESO-1-specific T cells in tumors per treatment group (**b**), expressed as a percentage of CD3 + TILs, (*n* = 7, error bars are SEM), *p < 0.0216 (MCLA-145 vs. Ly95), determined by one-way-ANOVA and Tukey’s test; **e** Ly95 T cells expressing an NY-ESO-specific TCR were transferred to NSG mice bearing A549-PD-L1^hi^ or A549-PD-L1^null^ tumors and treated with indicated antibodies, end point data are shown. (*n* = 9 mice), **p < 0.0018 MCLA-145 treatment (A549-PD-L1^hi^ vs A549-PD-L1^null^ groups), determined by one-way-ANOVA and Tukey’s test. **f** Tumor volume in individual human CD34+ engrafted NSG mice following grafting with MDA-MB-231 cells and treatment with indicated concentrations MCLA-145 or reference antibody (*n* c= 9 mice per group). Tumor growth and moment of death are indicated for the individual mice per group, a representative experiment of two independent experiments is shown, *p < 0.05, **p < 0.005, ***p < 0.001 of treatment group vs negative control antibody, determined by a two-sided mixed-effects model and Dunnett’s test. **g** Proportion of CD8+ (upper panel) and CD4+ (middle panel) TILs in tumors from each treatment group, expressed as a percentage of the total population of tumor cells, bottom panel, proportion of PD-L1+ monocytes (bottom panel) in tumors per treatment group, expressed as a percentage of all monocytes (CD11b+ cells), (*n* = 7 mice per group, error bars are mean ± SD, *p = 0.0492 for CD8+ T cells and *p* = 0.0014 for PD-L1^+^ monocytes, determined by one-way ANOVA and Tukey’s test.

To investigate the activity of MCLA-145 under therapeutically relevant conditions, fresh tumor explants containing tumor-specific effector T cells and Tregs were evaluated. Five primary endometrial tumors were dissociated into a single-cell suspension and analyzed by flow cytometry for cell counts or incubated with MCLA-145 or reference antibodies in the presence of soluble anti-CD3 antibody to measure IFNγ production. The percentage of CD3^+^ T cells, the proportion of CD4^+^, CD8^+^ and Treg subsets and the level of IFNγ production was heterogeneous between tumor samples (Fig. 4a). Treatment with MCLA-145 increased IFNγ production in all tumor samples relative to control antibody and, to a lesser extent, comparator antibodies. This effect was observed even in the context of high Treg numbers (Fig. 4a), consistent with our in vitro results (Fig. 3f). Like our observations in the SEB assay (Supplementary Fig. 1c), small numbers of PD-L1^hi^ myeloid and/or tumor cells (Supplementary Fig. 4a) appear to be sufficient to drive MCLA-145 activation of T cells and cytokine release.

Efficacy evaluation of MCLA-145 in vivo was carried out in murine xenograft models. The binding arms of MCLA-145 do not cross react with the murine homologues of PD-L1 and CD137, so two different strategies were used to humanize the murine xenograft models. In the first model, a human T cell clone expressing an NY-ESO-specific TCR (Ly95) was adoptively transferred into immunodeficient mice bearing human A549 tumors. The NSCLC cell line A549 expresses NY-ESO antigen in the appropriate HLA context^37^ and was modified for the study to stably express high levels of PD-L1 (Supplementary Fig. 4b). Ly95 cells express PD-1 and CD137 and a greater percentage of double-positive Ly95 cells were observed in tumors at the end of the experimental period (Supplementary Fig. 4c). In mice engrafted with A549 PD-L1^hi^ cells, treatment with urelumab* and atezolizumab alone or in combination along with Ly95 cells did not significantly alter tumor growth compared to control (Fig. 4b). All mice treated with MCLA-145 were able to control tumor growth (Fig. 4b). Importantly, MCLA-145 dependent tumor growth inhibition was clearly associated with a skewed distribution of adoptively transferred huCD3^+^ Ly95 cells to tumor vs blood (Fig. 4c) and increased NY-ESO antigen-specific T cells within the tumor compared to mice treated with controls (Fig. 4d).

To further explore the context-dependent nature of MCLA-145 activity, a second A549 line was prepared in which PD-L1 was knocked out (Supplementary Fig. 4b). MCLA-145 was unable to activate T cells when co-cultured with the A549 PD-L1^null^ line in contrast to A549 PD-L1^hi^ cells (Supplementary Fig. 4d). Similarly, MCLA-145 treatment in vivo had no effect on A549 PD-L1^null^ tumor size compared to control and in contrast to A549 PD-L1^hi^ tumors (Fig. 4e).

To evaluate the impact of MCLA-145 treatment in the context of a more heterogenous T cell population, we engrafted NSG mice with human CD34+ hematopoietic stem cells and then implanted the PD-L1 expressing human MDA-MB-231 breast cancer line (Fig. 4f). In animals receiving control IgG, tumors grew progressively over the first 40 days after which more rapid growth kinetics were observed in most animals (Fig. 4f). The group treated with CD137 agonist, urelumab* showed a very similar tumor growth pattern to control. In the groups treated with pembrolizumab, atezolizumab or the combination of atezolizumab and urelumab* there was evidence of tumor growth control in some mice but, by the end of the observation period, tumors in all surviving mice were growing rapidly (Fig. 4f). In contrast, all animals treated with MCLA-145 experienced tumor control including two complete responses and showed significantly slower growth kinetics at the end of the treatment period (Fig. 4f). A cohort of mice treated with a 10-fold lower dose of MCLA-145 (0.5 mg/kg) also showed similar inhibition of tumor growth (Fig. 4f). Consistent with the NY-ESO model, analysis of TILs in this model showed that MCLA-145 treatment resulted in an increased frequency of CD8^+^ T cells in the tumor (Fig. 4g). Strikingly, and consistent with the NY-ESO model, urelumab* treatment alone significantly reduced the number of CD8^+^ cells in the tumor compared to MCLA-145 and this was associated with an increase in the frequency of CD4^+^ T cells and PD-L1^+^ monocytes (Fig. 4g). These results demonstrate that the potent T cell agonism observed with MCLA-145 in vitro also translated into PD-L1 dependent tumor control in vivo.

We also noted that some mice in the pembrolizumab, atezolizumab or urelumab* groups and all mice in the combination treatment group exhibited ruffled fur and skin and a hunched posture, in some cases associated with excessive weight loss (Supplementary Fig. 4e), that required euthanasia (3–5 mice in each group). No animals in the control or MCLA-145 groups showed signs of stress (although one animal in the MCLA-145 group was euthanized for unrelated reasons). In particular, we observed that the combination treatment of urelumab* and atezolizumab was significantly more toxic than the control or MCLA-145 treatment (Supplementary Fig. 4f). One possible explanation for the observed toxicity is an acceleration of graft vs host disease (GvHD) which has been reported for ICI agents^38^. If true, the apparent lack of GvHD in MCLA-145 treated mice may be attributed to its tumor-restricted T cell agonism.

### MCLA-145 is well tolerated in non-human primates at high doses

**Fig. 5:**
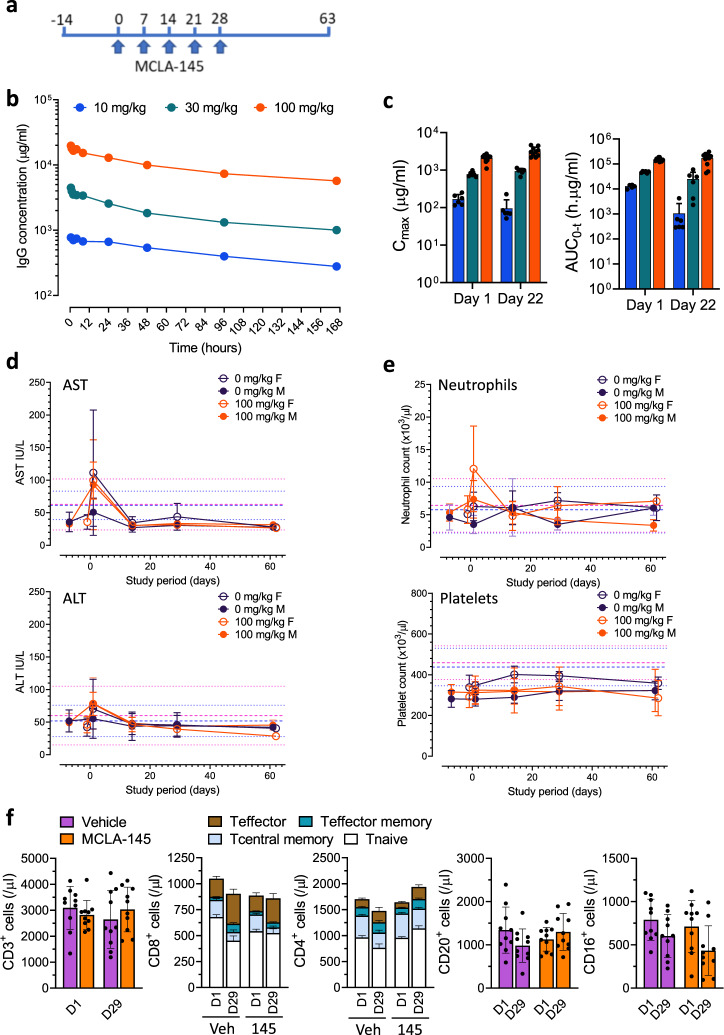
Safety of MCLA-145 in a repeat dose non-human primate study. **a** Schematic showing the design for the repeat dose safety study; **b** mean serum concentrations of MCLA-145 measured over one cycle (*n* = 10 animals for vehicle and 100 mg/kg, *n* = 6 animals for other treatment groups); **c** mean maximum serum concentration (*C*_max_) and area under the curve (AUC) at 10 mg/kg (blue), 30 mg/kg (green) and 100 mg/kg (orange) of MCLA-145 measured 24 h after the first dose and 24 h after the fifth dose combined for males and females; *n* = 10 animals for vehicle and 100 mg/kg, *n* = 6 animals for other treatment groups, error bars are mean ± SD); **d** Mean serum concentrations of AST and ALT and **e** mean number of circulating neutrophils and platelets measured over the study period at the indicated doses (*n* = 10 animals for vehicle and 100 mg/kg, *n* = 6 animals for other treatment groups, error bars are SD). Reference mean (male and female bold blue and pink lines respectively) and mean +1 or −1 SD (male and female bold blue and pink lines respectively) as previously reported;^69^ **f** Number of circulating B (CD20^+^), NK (CD16^+^) and T cells (CD3^+^) with CD4^+^ and CD8^+^ T cell numbers and proportion of different subpopulations shown for the 100 mg/kg/week dose group or vehicle, mean ± SD of male and female groups combined (*n* = 10 animals).

To further investigate safety, MCLA-145 was evaluated in non-human primates. MCLA-145 is fully cross-reactive with the cynomolgus homologues of PD-L1 and CD137 (Supplementary Fig. 5a, b) and showed similar functionality in a cynomolgus whole blood SEB assay (Supplementary Fig. 5c). In a repeat dose study, male and female cynomolgus monkeys received five weekly doses of MCLA-145 with a subset of animals being observed for a further 31 (males) or 32 (females) days (Fig. 5a). All dosed animals were shown to be exposed to MCLA-145. Pharmacokinetic analysis of MCLA-145 showed dose-proportional systemic exposure with a PK profile and half-life consistent with standard monoclonal IgG antibodies (Fig. 5b). Exposure increased with escalating dose in a manner that was dose-proportional on Day 1, and greater than proportional on Day 22, reflecting the loss of exposure in the lower dose levels after repeat dosing (Fig. 5c). In the 100 mg/kg/week dose group 70% of animals showed continuous exposure throughout the treatment period. The systemic exposure data are consistent with the expected anti-drug antibodies (ADA) titers observed in animals receiving lower doses. MCLA-145 was shown to be well tolerated at all doses up to the maximal dose of 100 mg/kg. No change in organ weight or any gross or microscopic pathological findings were noted. Blood chemistry was within normal ranges at all doses and time points including the liver enzymes AST and ALT (Fig. 5d). There was no effect on hematological parameters including neutrophil and platelets numbers (Fig. 5e). An integrated review of safety data from clinical trials of the agonist mAb urelumab noted elevated transaminases, neutropenia, thrombocytopenia as the most common serious adverse events along with fatigue^27^. None of these symptoms were observed even at the highest dose tested which is 1000 times higher than the maximum tolerated dose (MTD) of urelumab in man (0.1 mg/kg). Liver toxicity has been associated with the appearance of immune infiltrates in the liver^39,40^. Pathological examination of the liver and other tissues did not show any evidence of increased immune infiltration. Minor fluctuations in the lymphocyte numbers and T cell subpopulations were noted in both the vehicle and 100 mg/kg/week dose group (Fig. 5f). Collectively these data demonstrate that MCLA-145 is capable of potent T cell agonism resulting in tumor growth reduction in vivo but does not appear to exhibit the safety liabilities that have been observed with other potent CD137 agonists.

### MCLA-145 transactivates CD137 in a PD-L1 dependent manner via receptor clustering

**Fig. 6:**
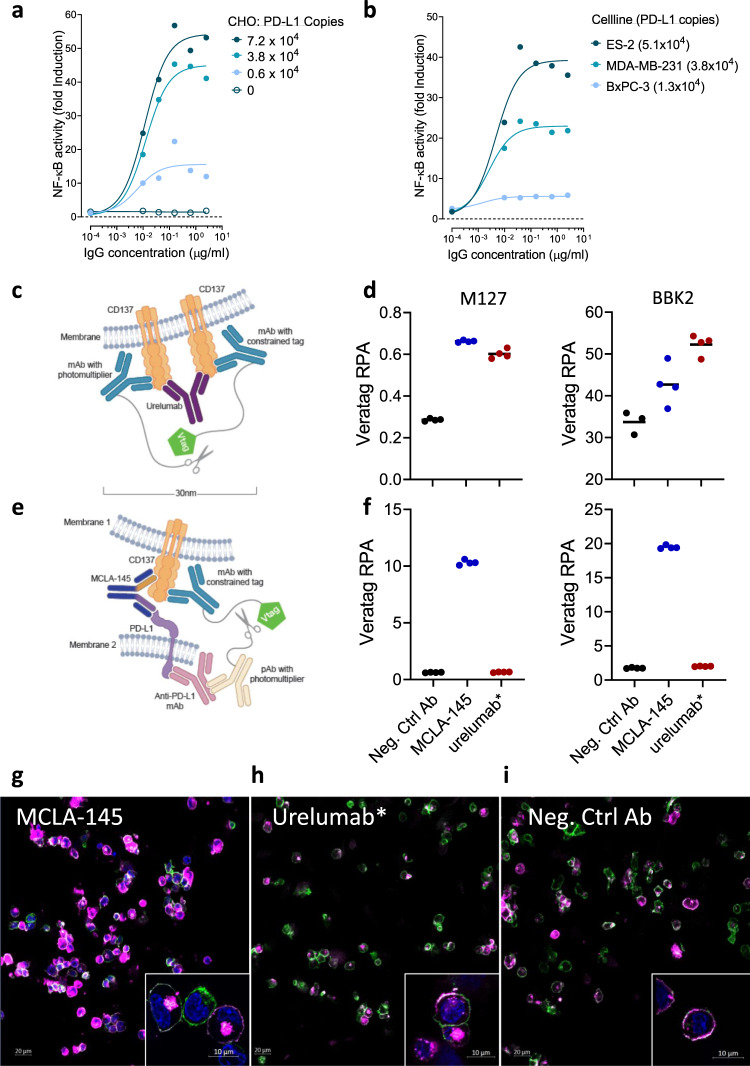
Colocalization of CD137 and PD-L1 mediates CD137 clustering. Fold induction of CD3-stimulated Jurkat NFκB-luc-CD137 reporter activity after 24 hours of co-incubation with either **a** CHO-PD-L1 cells or **b** human tumor cells endogenously expressing PD-L1 at increasing concentrations of MCLA-145; numbers in brackets indicate PD-L1 binding sites per cell as determined by QIFIKIT analysis; **c** Schematic illustrating the VeraTag assay to measure CD137 clustering; **d** CD137-expressing activated Jurkat T cells and PD-L1-expressing CHO cells cocultured with 10 µg/mL test antibody, fixed and measured for CD137 proximity using two different CD137 detection antibodies; **e** Schematic illustrating the VeraTag assay to measure CD137 and PD-L1 proximity; **f** Cells cultured and prepared as in (**d**) to measure PD-L1 and CD137 in proximity, RPA relative peak area; confocal images of CD137 receptor internalization in cocultures of activated CD8+ T cells and CHO-PD-L1^+^ incubated with (**g**) MCLA-145, (**h**) urelumab* or (**i**) negative control antibody for 15 min, nuclear (DAPI) staining (blue), CD8 (green), CD137 (magenta). Single plane view, magnification ×20 (insets ×63). Data are representative of two repeat experiments for Fig. 6g–i.

Under no experimental setting could we show that MCLA-145 was able to activate CD137 without the presence of PD-L1 expressed on an accessory cell. To investigate the effect of PD-L1 expression level on MCLA-145 agonistic activity, experiments were conducted with the reporter assay Jurkat cells co-cultured with CHO cell lines overexpressing human PD-L1 at different protein levels (Fig. 6a) or human tumor cell lines with different levels of endogenous PD-L1 expression (Fig. 6b). In both settings, there was a direct relationship between PD-L1 expression on accessory cells and MCLA-145 mediated CD137 receptor activation. Based on this set of experiments, we estimate a threshold of >5000 molecules of PD-L1 per cell is required to drive CD137 receptor clustering and activation.

The natural ligand for CD137 is a trimer and engages three copies of CD137 at the cell surface resulting in the clustering of CD137 to trigger downstream signaling. Agonistic CD137 mAbs are similarly capable of activating CD137 via clustering of receptor dimers resulting in rapid internalization and continued signaling of the receptor complex^41^. We performed receptor proximity assays (RPA) to address experimentally whether MCLA-145 induces CD137 clustering. RPA is capable of quantifying protein expression and dimerization in formalin-fixed, paraffin-embedded (FFPE) tissues or cells^42^. Expression of cell surface targets can be detected directly using this method and the signal for both CD137 and PD-L1 was equivalent in the presence of MCLA-145 and controls (Supplementary Fig. 6a–c). In the co-culture assay system, CD137 expression was specifically detected on activated Jurkat cells, and detection of PD-L1 was restricted to CHO-PD-L1 cells (Supplementary Fig. 6d–e). We first probed the cocultures of T cells and CHO-PD-L1 cells for the presence of CD137 clusters (schematically illustrated in Fig. 6c). MCLA-145 was shown to enhance clustering of CD137 (Fig. 6d). The activity of MCLA-145 was comparable to the agonist antibody urelumab* that is active without crosslinking or accessory cells (Fig. 1a). We next focused on the measurement of PD-L1 and CD137 in proximity (experimental set up schematically illustrated in Fig. 6e). Based on published structures, the distance between the CD137 epitope of MCLA-145 and the cell membrane is estimated to be 8–9 nm and for the PD-L1 epitope 7–8 nm; the opposing Fab arms of an IgG1 can reach 12 nm apart. Thus MCLA-145 could be expected to bring the two surface molecules within range of the proximity assay detection (30–100 nm)^42^. Indeed PD-L1 and CD137 proximity could only be measured in co-cultures of Jurkat and CHO-PD-L1 cells treated with MCLA-145; addition of urelumab* and negative control antibody induced no signal above background (Fig. 6f). These data indicate that binding of MCLA-145 translocates CD137 and PD-L1 towards a contact zone between neighboring cells resulting in CD137 clustering.

To investigate whether MCLA-145 triggers CD137 clustering and internalization in the presence of PD-L1 expressing cells, we performed confocal imaging of co-cultures of activated human primary CD8^+^ T cells with CHO-PD-L1 or CHO-WT cells (Fig. 6g-i). In co-cultures with CHO-PD-L1 cells, an intense punctate staining pattern of CD137, indicative of receptor clustering and internalization, was visible on CD8^+^ T cells 15 min after treatment with MCLA-145 (Fig. 6g) or urelumab* (Fig. 6h) but not a negative control antibody (Fig. 6i). In co-cultures with CHO-WT cells, treatment with MCLA-145 failed to induce CD137 clustering or internalization (Supplementary Fig. 6f). A 3D rendering of confocal images shows MCLA-145 activated CD8^+^ T cells with punctate CD137 staining at the interface of a monolayer of CHO-PD-L1^+^ cells (Supplementary Fig. 6g). Clustering and internalization results in brighter CD137 staining, which is significantly enhanced in MCLA-145 treated co-cultures compared with urelumab* (Supplementary Fig. 6h), consistent with our in vitro functional data. Collectively these data show direct evidence of MCLA-145 induced CD137 clustering resulting in receptor internalization that is dependent on adjacent cells expressing PD-L1 above a threshold of >5000 molecules per cell.

## Discussion

ICI therapies are now standard of care in many cancer indications and result in remarkably durable responses. However, most patients receiving ICI therapies do not respond to treatment and many whom do respond eventually relapse^43^. New approaches to enhance anti-tumor immunity are urgently needed. Pharmaceutical intervention to activate co-stimulation pathways has long been hypothesized as a logical companion to ICI therapies analogous to “hitting the gas and releasing the brakes”^44^.

**Fig. 7:**
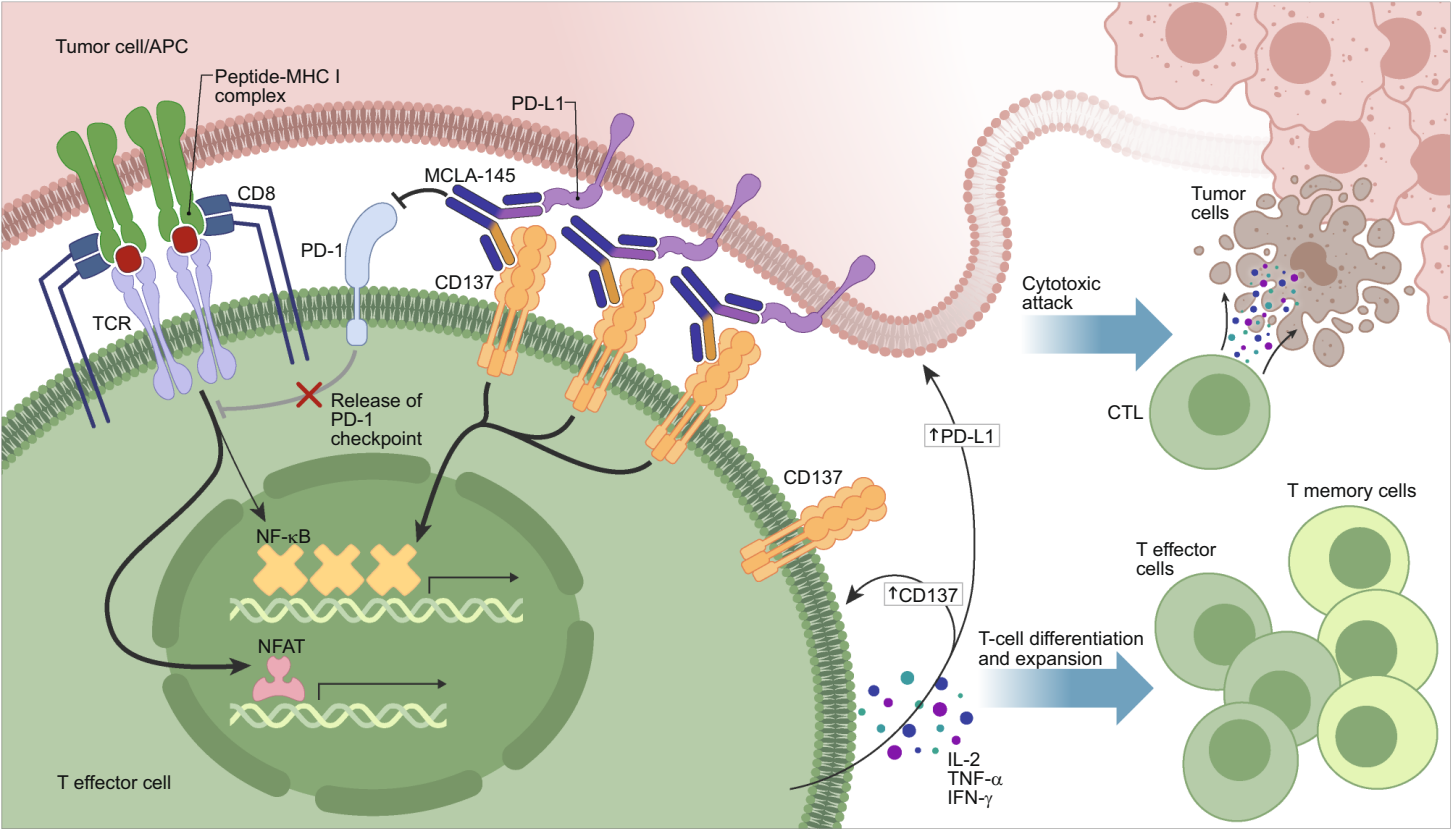
Schematic of the MCLA-145 mechanism of action. MCLA-145 is shown binding to PD-L1 expressed on a tumor cell or APC (depicted in pink) and CD137 expressed on a T effector cell (depicted in green). Engagement of MCLA-145 results in clustering of CD137 molecules on the surface of the effector T cell and downstream activation of the NF-kB signaling pathway. In addition, engagement of PD-L1 by MCLA-145 blocks interaction with its receptor PD-1 reversing PD-1 dependent suppression of downstream signaling from the T cell receptor complex. The net effect of MCLA-145 binding in trans to tumor cell/APC and T effector cells is shown: upregulation of CD137 (on T cells) and PD-L1 (on tumor cells and APCs), the release of pro-inflammatory cytokines from effector T cells resulting in T cell differentiation and expansion and activation of effector T cells to promote cytotoxic attack of tumor cells.

A number of T cell costimulatory molecules including CD137, OX40, CD40, ICOS, GITR, and CD27 are being targeted pharmacologically with agonists to improve outcomes in cancer patients^24,45^. They are transiently expressed upon ligation of the TCR with cognate antigen (e.g., tumor neo-antigens). Clustering of these receptors results in downstream signaling cascades that enhance T cell activity. Targeting CD137 to improve therapeutic response is clinically validated based on its application in CAR-T and TIL therapies^46,47^. Activation of the CD137 pathway appears to favor extending persistence and activity of pre-existing tumor-specific CD8+ T cell responses^14^. Relative to CD28 (also sometimes used in CAR-T constructs) CD137 co-stimulation expands memory CD8+ T cells and results in longer retention of effector T-cell functions^48^. Unfortunately, it has been difficult to translate the success in cell-based therapies to antibody-based therapeutic approaches targeting CD137. Strong agonists such as urelumab are hampered by major dose-limiting liver toxicity^27^ while other antibody approaches that depend on FcγR cross-linking (e.g., utomilumab) are weakly active preclinically and have not shown clinical activity even in combination with checkpoint inhibitors^29^. Here we describe an unbiased functional screen evaluating a large panel of bAbs targeting the PD-1/PD-L1 axis and the costimulatory receptor CD137. Applying this approach, MCLA-145 was identified as a potent T cell agonist with several advantageous mechanistic attributes. Our screen included antagonistic Fab arms to PD-1 and PD-L1 in combination with a panel of Fab arms binding across the ectodomain of CD137, and we evaluated T cell activity alone or in co-culture. We observed that only specific combinations of PD-L1 and CD137 binding arms in the bAb format were capable of potent T cell activation and only in the context of accessory cells expressing PD-L1. These bAbs all bind to the CRD2 of CD137, a region that is also associated with CD137L binding. Indeed, fine epitope mapping carried out with MCLA-145 identified critical binding residues that overlap with the CD137L epitope, and cross-blocking assays showed that MCLA-145 inhibits CD137L binding. We hypothesize that the CRD2 epitope bound by both MCLA-145 and CD137L may be the optimal for “trans” based engagement and activation of CD137. Furthermore, MCLA-145 engagement could drive the formation of an ‘immunological synapse’, as demonstrated for other bispecific constructs^49^. In the MCLA-145 driven synapse, T cells would benefit from effective TCR signaling relieved of PD-1 inhibition and potent CD137 activation (see Fig. 7 for a schematic representation). In this way, MCLA-145 may effectively and specifically promote antigen presentation and target cell lysis^50,51^.

MCLA-145 is a fully human IgG1 based bAb with substitutions in the Fc region to prevent FcR binding and thus higher-level crosslinking via FcR bearing cells. A prevailing assumption has been that trivalent^52,53^ or at least bivalent engagement^54,55^ is required to optimally cluster and activate members of the TNFRSF. We demonstrate that monovalent engagement of CD137 with a bAb anchored in trans is capable of clustering CD137 followed by rapid internalization and potent activation of T cells. The clustering and internalization activity induced by MCLA-145 resembled urelumab* in our experiments and CD137 agonist mAbs described in other studies^41^. The monovalent engagement of CD137 in this context has several potential advantages. Monovalent constructs often demonstrate a greater level of cell binding compared to bivalent constructs because in the latter case the two binding arms may sterically compete at higher concentrations. This property is likely to be an advantage as T cells and PD-L1-bearing cells are brought in proximity. Even under saturating conditions MCLA-145 is not able to activate CD137 without the presence of a neighboring cell expressing PD-L1. In addition, T cell activation required signal 1 (i.e., TCR stimulation via CD3 mAb or polyclonal activation). Furthermore, we show that the monovalent binding to CD137 places strict criteria on the number/density of PD-L1 molecules required to trigger the CD137 clustering and signaling. We estimate that expression of >5000 PD-L1 molecules per cell is required to promote effective engagement and activation of CD137 by MCLA-145.

Using a battery of orthogonal functional assays, we demonstrate MCLA-145 engages CD137 and PD-L1 to enhance T cell responses relevant for anti-tumor immunity. In a T cell priming assay where PD-1/PD-L1 axis inhibition has no functional impact, MCLA-145 increased antigen-specific T cell numbers in comparison to negative control antibodies and urelumab*. In some donors we observed a clear expansion of the effector memory compartment consistent with the unique role CD137 has among the TNFRSF costimulatory receptors in promoting durable antigen-specific CD8^+^ T cell responses^13,33,56^. Conversely, in a CD4^+^ T cell recall assay, where urelumab* treatment was ineffective, we showed MCLA-145 to be more active than atezolizumab*. These experiments demonstrate that both targeting arms of MCLA-145 are functionally active. In more heterogenous cell-based assays, such as the SEB and MLR assay, the synergy of targeting both CD137 and PD-L1 pathways was even more apparent and we show MCLA-145 is capable of T cell activation comparable to the combination of urelumab* and atezolizumab*.

Tumors in patients unresponsive to ICI therapy are often characterized by a lack of T cell infiltration and/or a highly immunosuppressive microenvironment^4,10^. Several pieces of evidence in this study suggest MCLA-145 may benefit patients with suboptimal responses to ICIs. First, we demonstrate in vitro that enhancement of T cell responses by MCLA-145, but not urelumab* + atezolizumab*, was evident in co-cultures of Tregs or M2 macrophages reversing their suppressive activity on T cell cytokine release. Secondly, in fresh tumor explants MCLA-145 increased T cell cytokine release in the presence of a significant proportion of Treg cells and this effect was greater than atezolizumab* in the majority of donors. We hypothesize that T cell hyperactivation induced by MCLA-145 in co-cultures containing PD-L1 bearing cells counteract the suppressive signals that are released by Tregs and TAMs. We note that CD137 expression is upregulated on Tregs^57^ and that CD137 agonism can modulate Treg activity^58^ thus MCLA-145 could be acting directly on the Treg population in addition to increasing effector T cell activity. Finally, in two different humanized xenograft models treated with MCLA-145, we demonstrated an expansion of CD8^+^ T cell numbers in tumors that were associated with greater tumor growth control than negative control or comparator antibodies. In the A549 model where human PD-L1 is only present on the tumor, we observed recruitment of adoptively transferred NY-ESO specific T cells from the periphery to the tumor and this was confirmed in the MDA-MB-231 model where PD-L1 expressing myeloid cells are also present. Unexpectedly, treatment with urelumab* alone in both models resulted in fewer tumor CD8^+^ T cells while increasing the number of CD4^+^ T cells and myeloid cells. While both models suffer the limitation of not providing a complete ‘human’ tumor microenvironment (e.g., with Tregs and TAMs), the differential effect of MCLA-145 on tumor growth and immune cell infiltration may point to a potential benefit of localized activation of CD137.

Systemic activation of CD137 by agonistic antibodies results in toxicity in animal models and humans^27,44^. We observed that immunocompromised mice engrafted with CD34^+^ human cord blood exhibited signs of accelerated GvHD when treated with urelumab*, atezolizumab or pembrolizumab. In some cases, the GvHD was severe enough to require euthanasia on the study. In contrast, MCLA-145 treated mice remained healthy throughout the treatment period, resembling negative control-treated animals. This suggests the context-dependent nature of MCLA-145 T cell activation did not extend to graft reactive T cells. In a GLP repeat dose non-human primate (NHP) study, MCLA-145 was well tolerated at suprapharmacological doses with no evidence of elevated liver enzymes, neutropenia or thrombocytopenia, these being the most common serious adverse events reported in clinical studies with urelumab^27^. Based on an integrated safety analysis, the MTD of urelumab was determined to be 0.1 mg/kg, a dose three orders of magnitude lower than the highest MCLA-145 dose tested in NHPs. To our knowledge, this is the first report of a fully human and cynomolgus cross-reactive CD137 agonist tested in NHPs at this dose level. While NHP studies have failed to pick up safety issues with immune agonists previously^59^, MCLA-145 was evaluated as safe at supraphysiological doses. Taken together with the rodent experiments our data suggest MCLA-145 will have a favorable safety profile in humans.

MCLA-145 is a targeted drug capable of both T cell costimulation and checkpoint blockade which may have broad clinical utility. The preclinical data presented suggest MCLA-145 has the potential to be active as a monotherapy in settings where ICI treatments fail to improve patient outcomes. MCLA-145 could also be utilized as an adjuvant to boost responses to cancer vaccines and other treatment modalities (e.g., chemotherapy) that promote neoantigen presentation in tumors or draining lymph nodes. Professional APCs express the highest levels of PD-L1^60,61^. Based on our data, MCLA-145 could be expected to enhance naïve T cell priming and promote long-term T cell immunity. Importantly, MCLA-145 is unlikely to have the same safety liabilities that have limited the clinical development of CD137 agonists. MCLA-145 is currently being evaluated in an open label, dose-escalation single-agent study with expansion cohorts for dose confirmation/safety and preliminary efficacy in advanced or metastatic malignancies (NCT03922204).

## Methods

### DNA constructs

Constructs expressing human PD-L1 and human CD137 were generated in the pVAX1 vector (Invitrogen, cat. no. V260-20) for DNA immunizations. Constructs expressing human PD-L1 and human CD137 as well as the rhesus macaque orthologues were generated in the pIRES-neo3 vector (Clontech, cat. no. 6988-1) for cell transfections. Chimeric domain-swapped constructs for CD137 were generated by swapping domains with one of the mouse orthologues in the pIRES-neo3 vector. All VHs derived from the phage selection process were cloned into an IgG1 backbone that lacked Fc effector function upon the introduction of amino acid variations in the CH2 regions^31^. All constructs were sequence verified.

### Immunizations

Transgenic mice, including strains capable of generating human common light chain (cLC) antibodies, were used in the study. These mice (referred to as MeMo mice) were immunized at varying intervals with recombinant huPD-L1-His (40 μg/mouse) in Gerbu Adjuvant MM (Gerbu Biotechnik c#3001) or with pVax-CD137 DNA. Sera were screened for binding to Freestyle 293F cells transfected with PD-L1 or CD137 constructs.

### Phage library generation, phage selection, and antibody production

Common light chain phage libraries were constructed from immunized mouse. From each individual mouse, RNA was isolated, cDNA was synthesized, and VH-family-specific PCRs were performed. Subsequently, all VH family PCR products per mouse were purified, digested, and ligated in a phage-display vector containing the common light chain to generate a phage library containing mouse-human chimeric Fab domains^62^. All phage libraries contained >10^6^ clones with an insert frequency of >85%. Phage libraries were rescued with VCS-M13 helper phage (Stratagene) and selected for two rounds in immunotubes (Nunc) coated with recombinant protein. Positive phage clones binding CD137 and PD-L1 were then identified by FACS for binding to Freestyle^TM^ 293-F cells and Freestyle 293F cells transfected with PD-L1 or CD137. The VH genes of all PD-L1 and CD137 specific clones were sequenced. VH gene rearrangements were established with VBASE2 software to identify unique clones. All unique clones were then tested in phage format for binding in FACS to Freestyle^TM^ 293-F cells (negative control) and Freestyle^TM^ 293-F cells transfected with PD-L1 or CD137. VH genes of unique antibodies, as judged by VH gene sequence and some sequence variants thereof, derived from the immunized mouse phage libraries were cloned in an IgG1 backbone vector^63^. IgGs were produced in transiently transfected Freestyle^TM^ 293-F (Invitrogen) cells according to the manufacturer’s instructions. bAbs were generated with a mutated IgG1 Fc variant region known as DEKK^30^ to ensure efficient hetero-dimerization and formation of a bAb. This technology uses charge-based point mutations (L351D/L368E and L351K/T366K) in the CH3 region to allow efficient pairing of two different heavy chain molecules. Antibodies were purified using protein A batch purification (Pierce). Antibodies were characterized based on their affinity in FACS titrations. The panel of CD137 antibodies was grouped or binned based on their binding to Freestyle^TM^ 293-F cells transiently transfected with mouse human CD137 chimeras. The PD-L1 antibodies were binned based on their capacity to block PD-1 PD-L1 interaction in a PD-L1 reporter bioassay (Promega).

### Antibody reagents

Reference antibodies were analogs of anti-CD137 monospecific antibodies urelumab and utomilumab, and anti-PD-L1 antibody atezolizumab and avelumab. These antibodies were generated by synthesizing their VH and VL sequences and recloning them into the appropriate IgG backbone vector. The atezolizumab analog was based on the information disclosed in WO2010077634A1, the urelumab analog was based on WO 2005/035584, the avelumab analog was based on WO 2016/137985 Al, whereas the utomilumab analog was based on information obtained from and WO2015119923. These analog reference antibodies are marked with an asterisk in the manuscript. When available clinical reagents were used for pembrolizumab and atezolizumab. Negative control antibodies were common light chain antibodies directed against tetanus toxoid (TT) or respiratory syncytial virus protein G (RSV-G). A complete list and information of the antibodies used in this study can be found in Supplementary Table 4.

### Human blood samples

Blood samples were obtained from two sources Sanquin Blood Supply (Amsterdam, the Netherlands) and BioIVT, Westbury, N.Y. Samples were collected from licensed and accredited facilities following IRB-approved protocols. Sanquin: All donors provided written informed consent in accordance with the Declaration of Helsinki and the protocol of the local institutional review board, the Medical Ethics Committee of Sanquin Blood Supply. BioIVT: all informed consent were documented in writing from each individual leukopak donor per the requirements of the Department of Health and Human Services regulations for the protection of human subjects (45 CFR §46.116 and §46.117) and Good Clinical Practice (GLP), (ICH E6). Mononuclear cells were isolated by Ficoll-Paque PLUS (GE Healthcare Life Sciences) density gradient centrifugation.

### Cell lines

All cell lines were maintained at 37 °C in a humidified atmosphere at 5% CO_2_, unless otherwise indicated in figure legends or method details. Human ES-2 cells (cat. no. CRL-1978) were purchased from ATCC and routinely maintained in McCoy’s 5A (Gibco) supplemented with 10% FBS (Lonza). Freestyle^TM^ 293-F cells (cat. no. p/n51-0029) were obtained from Invitrogen and routinely maintained in 293 FreeStyle^TM^ medium. The CHO-K1 cell line (cat. no. DSMZ ACC110) was obtained from DSMZ and routinely maintained in DMEM/F12 (Gibco) supplemented with L-Glutamine (Gibco) and 10% FBS (Lonza). MDA-MB-231 cells (cat. no. CRM-HTB-26) were obtained from ATCC and routinely maintained in DMEM high glucose (Gibco) supplemented with 100 mM sodium pyruvate (Gibco), MEM non-essential amino acids (Gibco), and 10% FBS (Lonza). BxPC-3 cells (cat. no. CRL-1687) were obtained from ATCC and routinely maintained in RPMI-1640 (Gibco) supplemented with 10% FBS (Lonza). A549-A2-ESO tumor cells are derived from a human lung cancer cell line and express the cancer-testis antigen NY-ESO-1 in the appropriate HLA context and were cultured in RPMI 1640 (Gibco) supplemented with 10% heat-inactivated fetal calf serum (FCS), 100U/ml penicillin, 100 µg/ml streptomycin sulfate, and 1% L-glutamine (complete cell culture medium).

### Generation of stable CHO-K1 cell lines expressing PD-L1 and CD137

pIRES-Neo3 expression constructs were used to generate CHO-K1 clones stably expressing CD137 and CHO-K1 clones stably expressing different levels of PD-L1. Constructs were transiently transfected into CHO-K1 cells using lipofectamine transfection and screened by FACS using antibodies specific for the respective proteins. After confirmation of expression, transiently transfected cells were seeded in limiting dilution and cultured under selection pressure relevant for the expression construct used to obtain stable cell clones. After 2–3 weeks of selection, clones were screened by FACS. Clones were selected based on the expression level of the different targets and renamed CHO-PD-L1 and CHO-CD137 cells.

### Generation of Jurkat CD137-NFκB-luc cell lines

A Jurkat CD137-NFκB-luc stable reporter cell line was generated by stably integrating the CD137 and the NF-κB luciferase reporter genes into Jurkat E6 cells. In short, the [pIRESneo3] vector (Clontech) containing the CD137 gene was transfected, and stable clones expressing CD137 were generated following antibiotic selection. Next, the NF-κB luciferase reporter constructs pGL4.32 [luc2P/NF-κB-RE/Hygro] (Promega) was transfected into the clone with the highest CD137 expression, and stable clones expressing both CD137 and NF-κB luciferase were selected following antibiotic selection. Clones were selected for their capacity to respond to CD137L after initial activation by plate-bound CD3 antibodies (clone OKT-3) and PMA/ionomycin. Jurkat CD137-NFĸB-luc reporter cells were cultured in 9% FBS-HI RPMI 1640, 2 mM L-Glutamine containing 500 µg/mL G418 (Sigma cat. no. G8168) and 400 µg/mL Hygromycin B gold (Invivogen, cat. no. ant-hg-1).

### Generation of PD-L1 high and PD-L1 knockout A549-A2-ESO cells

To generate PD-L1 knockout cells, A549-A2-ESO cells were nucleofected using Lonza 4D Nucleofector with Cas9-gRNA protein RNA complex for CRISPR-mediated genome editing. 3–4 days after nucleofection, A549-A2-ESO cells were treated with 10 ng/mL human IFNγ for 24 h, before human PD-L1 cell surface expression was analyzed using a Novocyte flow cytometry instrument. Human PD-L1 knockout cells were then sorted twice using a BD FACSAria Fusion sorter. After 1 week of cell expansion and stimulation with IFNγ, Novocyte flow cytometry confirmed that >95% of cells were PD-L1 knockout. A549-A2-ESO-1-PD-L1-hi cells were generated by adding high titer 3rd generation lentivirus encoding for human PD-L1 at 5:1 MOI (multiplicity of infection) to A549-A2-ESO tumor cells in logarithmic growth. The tumor cells were grown for one week at which point they were subjected to flow cytometric sorting for HLA-A2-positive and PD-L1-high expressors.

### Confocal imaging of MCLA-145-induced CD137 clustering

Induction of CD137 receptor clustering by MCLA-145 was imaged using a co-culture of CHO cells over-expressing human PD-L1 and primary CD8+ T cells isolated from healthy donors. Briefly, CD8+ cells were enriched from PBMCs using a negative selection kit (Miltenyi Biotec). The CD8+ T cells were activated with CD3/CD28 magnetic beads for 3 days, and upregulation of CD137 was monitored by flow cytometry. 50,000 T cells were added to CHO-PD-L1 cells at a ratio of 1:10 (CHO-PD-L1 to T cells), previously plated on CellView Cover Slip bottom chamber slides (cat. no. 543979, Greiner Bio-One North America Inc.) in the presence of test antibody (5 µg/mL). Test antibodies were MCLA-145, urelumab*, a human IgG4 isotype control antibody (Biolegend), and single-arm control antibodies CD137xTT and PD-L1xTT. The single-arm control antibodies contain the same CD137 and PD-L1 Fabs that are present in MCLA-145 in combination with a non-targeting Fab arm directed to Tetanus Toxoid. Cells were incubated for 5 and 15 min at 37 °C and fixed with 2× PFA (4% final concentration). The cell was permeabilized and stained with anti-CD137-Alexa 647 (Biolegend, 309823), anti-CD8 –Alexa 488 (BD Pharmingen, #557696), and Actin filaments were labeled with Texas Red™-X Phalloidin (Thermo Fisher Scientific, #T7471). Images were acquired with a ×20 air objective and a 63x oil immersion objective on a Zeiss 880 confocal microscope. Z-stacks were acquired using 0.25 µm intervals to include both CHO-PD-L1 and T cells.

### **M**ouse experiments

All experiments were performed according to institutional guidelines and were approved by the Incyte Corporation and University of Pennsylvania institutional animal care and use committee (IACUC).

### CD34+ NSG mice bearing MDA-MB-231 tumors

To generate human stem cell-engrafted NSG mice, immunodeficient female NSG mice (6–8 weeks of age; The Jackson Laboratory, Bar Harbor, ME, stock# 705557 JAX, co-housed under barrier conditions) received 15 mg/kg intraperitoneal busulfan (Busilvex, Pierre Fabre) and 24 h later an intravenous injection containing 1 × 10^5^ human CD34+ cord blood cells (purchased from STEMCELL Technologies). The experiment only included mice that had >25% huCD45^+^ cells and T cell levels above 80 counts/µL in peripheral blood. Mice were inoculated subcutaneously with a total of 3 × 10^6^ MDA-MB-231 tumor cells suspended in 100 μL of serum-free culture medium and matrigel matrix (Corning) in equal volumes. When tumors reached ~80–100 mm^3^, mice were randomized into the following groups (*n* = 7 per group): PBS (vehicle control), Fc-silenced IgG1 control (5 mg/kg), atezolizumab (5 mg/kg), urelumab* (5 mg/kg), equimolar mix of atezolizumab and urelumab* (5 mg/kg), and MCLA-145 (5 mg/kg). Antibodies were diluted in PBS (Life Technologies) and administered intraperitoneally. Animals were dosed intraperitoneally once every 5 days for a period of 31 days. Tumors were measured using calipers, and tumor volume was calculated by assimilating them to an ellipsoid using the formula: l (length) × w^2^ (width) × ½. Statistical significance was determined by one-way ANOVA. Body weights were also monitored throughout the study.

### Ly95-NY-ESO adoptive T cell transfer model

This mouse model makes use of human transgenic Ly95 T cells that resemble patient TILs. This enables the effects of antibody treatment on such TILs to be studied in a non-human model that mimics the human tumor microenvironment^37^. The Ly95 T cells express an optimized TCR (called Ly95) that is directed against a peptide within the cancer-testis antigen NY-ESO-1^63,64^. The tumors in this model express the NY-ESO-1 antigen in the appropriate HLA context. NSG female mice (6–8 weeks of age; The Jackson Laboratory, Bar Harbor, ME, stock# 005557 JAX, co-housed under barrier conditions) were first inoculated subcutaneously with 5 × 10^6^ A549-A2-ESO tumor cells suspended in 100 µL serum-free culture medium and matrigel membrane matrix (Corning) in equal volumes. After tumors were established (mean volume of 150 mm^3^), the mice were randomized into six groups of 7 mice whereby one group received a single intravenous tail-vein injection of PBS alone, and five groups were injected with PBS containing 10 × 10^6^ NY-ESO1-reactive Ly95 TCR construct-expressing human T cells. The five groups that had undergone adoptive transfer with the tumor-specific transgenic Ly95 T cells were subsequently treated intraperitoneally every five days with PBS, atezolizumab (5 mg/kg), urelumab* (5 mg/kg), equimolar mix of atezolizumab and urelumab* (5 mg/kg), or MCLA-145 (5 mg/kg). Over a period of four weeks, tumor volume was recorded twice a week using a study log system.

### PD-L1-dependent Ly95-NY-ESO adoptive T cell transfer model

The details of this model are the same as those of the Ly95-NY-ESO adoptive T cell transfer model with the following exception: two variants of the A549-A2-ESO-1 tumor cell line were used. One variant had been transduced to stably express high levels of PD-L1 (A549-PD-L1^hi^) and another variant lacked PD-L1 expression due to PD-L1 gene knockout via CRISPR-Cas9 (A549-PD-L1^null^).

### Cynomolgus monkey toxicity study

Cynomolgus monkeys (*Macaca fascicularis*) were given 5 once-weekly IV doses of MCLA-145 (0, 10, 30, or 100 mg/kg). The 10 and 30 mg/kg groups consisted of three males and three females. The control and high-dose groups consisted of five males and five females, including two recovery animals. Main study animals were sedated using ketamine, terminally sedated with sodium pentobarbital, and euthanized by exsanguination on days 31 or 32, and recovery animals were maintained for a 4-week off-dose period to evaluate any potential delayed onset or reversibility of toxicity. Animals were monitored for clinical signs, including injection site reactions, body weight, and ophthalmoscopy. Safety pharmacology endpoints (ECG, blood pressure, body temperature, and respiration rate) were assessed pre-dose and post-dose on day 8. Laboratory investigations (hematology, coagulation, clinical chemistry, and urinalysis) were performed prior to study start as well as on days 2, 15, and 30 and in week 4 of the recovery period. Lymphocyte subset analysis (immunophenotyping) was performed on blood samples collected prior to study start as well as on days 2, 15, 30 and at the end of the recovery period. At study termination (days 31/32 for main study animals, days 31/32 of the recovery period for recovery animals), all cynomolgus monkeys were subjected to full macroscopic postmortem examination and organ weight recording, and a full list of tissues was taken for histopathological examination.

The experiments in cynomolgus monkeys were conducted at Covance (Harrogate, UK) in accordance with the requirements of the Animals (Scientific Procedures) Act 1986 (UK), and approved by the Covance Institutional Ethical Review Committee.

### Pharmacokinetics

On day 1 of the toxicology study described above, pharmacokinetics was evaluated in all cynomolgus monkeys treated with MCLA-145 (10, 30, or 100 mg/kg). Blood samples were obtained at 0 (pre-dose), 0.5, 1, 3, 6, 24, 48, 96, and 166 h after dosing. MCLA-145 serum concentrations were determined via an electrochemiluminescence (ECL) assay (MSD) using a dual-binding ELISA. The pharmacokinetic parameters were derived by non-compartmental analysis using Phoenix WinNonlin software (Certara).

### Statistical tests

All statistics were calculated using Graphpad Prism Version 5 for Windows. Statistical significance was determined using one-way or two-way ANOVA to compare differences among multiple groups. For multiple comparisons, a post hoc Tukey’s or Dunnett’s test (as indicated in figure legends) was applied when comparing a control with multiple groups, and a Holm-Sidak method was used for comparisons between all groups. *P*-values < 0.05 were considered statistically significant.

### Binding kinetics and affinity using SPR

All SPR experiments were run on a Biacore T200 instrument at an analysis temperature of 25 °C and controlled by T200 control software. SPR running buffer was 10 mM HEPES, 150 mM NaCl, 3 mM EDTA, and 0.05% v/v Surfactant P20, pH 7.4, prepared from 10× HBS-EP + Buffer (Biacore). An anti-human IgG chip was prepared by immobilizing anti-human IgG Fc antibody (GE Healthcare BR100839) via amine coupling on all four flow cells of a CM5 chip (Biacore). Immobilization levels were ~9000 resonance units (RU) on flow cells 1 and 3, and ~7000 RU on flow cells 2 and 4. MCLA-145 and reference antibodies were captured using a flow rate of 1 μg/mL on flow cells 2 or 4 for 30 s, resulting in a capture level of ~100 RU.

A concentration series of human PD-L1 in SPR running buffer was prepared in a 3-fold serial dilution (total 6 concentrations, highest at 64 nM) of human PD-L1 stock (R&D 9049-B7-100, 500 μg/mL 19.2 μM, reconstituted with running buffer) or cyno PD-L1 stock (ACRO Biosystems PD1-C52H4, 400 μg/mL, 14.8 μM). Running buffer without PD-L1 was used as a control (0 concentration). The injection was for 180 s (contact time), immediately followed by running buffer for 240 s (dissociation time) at a flow rate of 30 μL/min. All experiments were run in 2-1 or 4-3 dual running mode. The surface was regenerated with a 30s injection of 3 M MgCl_2_.

A concentration series of CD137 in SPR running buffer was prepared in a threefold serial dilution (total 7 concentrations, highest at 92.7 nM) of human CD137 stock (R&D 9220-4B-100, 500 μg/mL 27.8 μM, reconstituted with running buffer) or cyno CD137 stock (lot 597923-FC-032018, 827 μg/mL, 45.9 μM or lot DGUK011803A, 1000 μg/mL, 55.6 μM). Running buffer without CD137 was used as a control (0 concentration). The injection was for 180 s (contact time), immediately followed by running buffer for 240 s (dissociation time) at a flow rate of 30 μL/min. All experiments were run in 2-1 or 4-3 dual running mode. The surface was regenerated with a 30 s injection of 3 M MgCl_2_.

Data of binding kinetics of human and cynomolgus PD-L1 and CD137 was analyzed using Biacore T200 Evaluation Software with double-reference subtraction (0 concentration and reference flow cell 1 or 3 with no antibody capture). Binding kinetics and affinity parameters were obtained from a global fit of the data to 1-to-1 binding model.

We also studied dual binding of human PD-L1 and CD137: after capture of MCLA-145 (~100 RU), one of three solutions (100 nM human PD-L1, 100 nM human CD137, or running buffer as control) was injected for 180 s, immediately followed by the injection of another of the three solutions for another 180 s, using the “dual” injection command. Binding data was analyzed using Biacore T200 Evaluation Software.

### Binding affinity determination using radiolabeling

Antibodies were radiolabeled with ^125^I using the method described by van Uhm et al.^65^. Radiolabeled IgGs had a specific radioactivity of ~10–25 GBq/µmol. Immuno-reactive fraction analysis performed according to Lindmo et al.^66^ with the labeled and non-labeled IgG using CHO-PD-L1 and CHO-CD137 cells showed that radiolabeling caused no reduction in binding, or only minor signs. Steady-state cell affinity measurements were performed in 96-well plates: CHO-PD-L1 cells were seeded at 4000 cells/well and CHO-CD137 cells at 16,000 cells/well. Various concentrations of radiolabeled antibodies were added and incubated at 4 °C for 3 h. Unbound radiolabeled antibodies were washed away, and cell-bound radioactivity was measured using a gamma counter (WIZARD2, PerkinElmer). Non-specific binding was measured by adding a receptor-blocking concentration (100-fold excess) of unlabeled antibody during incubation with radiolabeled antibody. Each condition was tested in triplicate and three independent experiments were performed per antibody. KD values were calculated based on a non-linear regression model that compensates for non-specific binding (Prism 7.0, GraphPad).

### Epitope mapping using shotgun mutagenesis

Alanine scanning mutagenesis^67^ was used to map the CD137 epitope of MCLA-145. Clones were generated by substituting each amino acid residue of the CD137 extracellular domains (ECDs) with alanine (native alanine residues were substituted with serine). For each clone and WT version, DNA was transfected into HEK293T cells. 24 h later, the reactivity of antibodies was measured by immunofluorescent staining, leading to binding maps and identification of critical residues for antibody binding. Fluorescently labeled secondary antibodies against human or mouse IgG were used to detect binding of MCLA-145 or mouse control antibody (BD Biosciences cat. no.555955). To identify clones that had a high level of CD137 expression but low binding to MCLA-145, MCLA-145 binding was compared with that of the relevant mouse positive control antibody. To identify preliminary critical clones, the following threshold was applied, i.e., >70% WT binding to control antibody and <20% WT reactivity to MCLA-145.

### T cell transactivation assay

The transactivation assay was performed with primary T cells or CD137-NFκB-luc reporter cells. For both transactivation assays, the inner wells of 96-well flat-bottom plates (Cellstar #655180) were coated overnight with 5 µg/mL anti-CD3 (OKT-3 eBioscience, cat. no. 16-0037-85) in PBS. T lymphocytes were isolated from PBMCs from healthy donors using the EasySep T cell enrichment (pan CD3) purification procedure as described by the manufacturer (Stem Cell Technologies, cat. no. 19051). The EasySep procedure uses negative selection. Next, OKT-3 coated plates were washed with PBS and 25 µL of the prediluted antibody was added, followed by 50 µL purified T cells (50,000 cells/well) and 25 µL CHO-PD-L1 cells (30,000 cells/well). Cells were allowed to incubate for 72 h at 37 °C. The supernatant was then collected and tested fresh or stored at −80 °C. Cytokine levels were measured in culture supernatants by AlphaLISA or Luminex Multiplex assay. IL-2 levels were detected by AlphaLISA in accordance with the manufacturer’s instructions (Perkin Elmer). Concentrations were calculated based on the standard curve. Alternatively, cytokine production in vitro was determined using multiplex analysis developed by eBioscience. Levels of IFNγ, IL-2, and TNFα were measured in culture supernatants following the manufacturers’ instructions. Results were analyzed by eBioscience analysis software.

Alternatively, OKT-3 coated plates were washed with PBS, and 25 µL of the prediluted antibody was added, followed by 25 µL CD137-NFkB-luc reporter cells (50,000 cells/well) and 25 µL CHO-PD-L1 cells (12,500 cells/well), followed by 25 µL assay medium. Each plate contained a serial dilution of negative control antibody (anti-TT) and positive control antibody that served as reference controls. Plates were incubated overnight at 37 °C, 5% CO_2_, in 95% relative humidity. 50 µL of luciferase (Promega, Bright-Glo™, cat. no. E2610) was added the next day, and the amount of luciferase activity was measured using a PerkinElmer EnVision Multimode Plate Reader.

### PD-L1 reporter assay (Promega)

The PD-1/PD-L1 blockade bioassay from Promega (thaw-and-use format) was performed according to the manufacturer’s instructions. The assay is based on a two-cell system requiring CHO cells expressing both PD-L1 and a T cell activator, and a Jurkat/NFAT-RE Reporter Cell Line overexpressing PD-1. Potency was calculated as luciferase activity relative to the background activity obtained using the negative control antibody (RSV-G) that was included in each plate.

### Whole blood SEB stimulation

Blood from healthy human donors or cynomolgus monkey (COVANCE) was collected in heparin tubes and processed the same day. Briefly, blood was diluted tenfold with AIM-V^®^ Medium (ThermoFisher Scientific) and stimulated with 5 ng/mL staphylococcus enterotoxin B (SEB; Toxin Technology, USA). Various concentrations of therapeutic antibodies were added, and after 3 days of culture, cytokine levels were assessed in the culture supernatant. Cytokine levels in human samples were determined using ProcartaPlex immunoassay (Th1/Th2/Th9/Th17/Th22/Treg Cytokine 18-Plex Human ProcartaPlex™ Panel; Thermo Fisher Scientific) and cynomolgus monkey samples by IFNγ ELISA (R&D Systems) according to the manufacturer’s recommendations. Results were expressed as the fold change in cytokine secretion relative to the level secreted in the absence of therapeutic antibodies.

### PBMC SEB stimulation

Human PBMCs were plated in RPMI 1640 medium with L-glutamine (Life Technologies) and 9% FBS at 200,000 cells/well in U-bottom 96-well plates. Cells were stimulated with 2 µg/mL SEB (Sigma, cat. no. S4881) for 37 °C, 5% CO_2_, in 95% relative humidity in the presence or absence of test antibodies. The supernatant was then collected and tested fresh or stored at −80 °C. Cytokine levels were measured in culture supernatants by AlphaLISA or Luminex Multiplex assay. In some experiments, unstimulated and SEB-stimulated PBMCs were harvested after 24 and 48 h and stained with fluorescently labeled antibodies (anti-CD3, -CD4, -CD5, -CD8, -CD11c, -CD14, -CD16, -CD19, -CD20, -CD56, -CD123, -HLA-DR, -CD137, and -PD-L1) to determine CD137 and PD-L1 expression levels of cell subsets by FACS analysis.

### Human MLR assay

Primary human CD3+ pan T cells were freshly isolated from PBMCs from healthy donors using Dynabeads™ Untouched™ Human T Cells isolation kit (Thermo Fisher Scientific) and mixed with previously frozen monocyte-derived DCs from three different donors (HemaCare) at a 10:1 T to DC ratio. To this end, 10^5^ T cells/well and 10^4^ DCs/well were plated in 96-well round-bottom plates (Corning #4515). The cell mixture was then incubated with test antibody for 6 days. IFNγ levels in the supernatant were measured by V-PLEX Human IFNγ Kit (Meso Scale Discovery).

### Memory T cell recall assay

Human PBMCs were isolated from the peripheral blood of healthy donors by standard density gradient centrifugation on Ficoll-Hypaque (GE Healthcare-Life Sciences, USA). Memory CD4^+^ T cells were enriched by magnetic beads separation (Memory CD4^+^ T Cell Isolation Kit; Miltenyi Biotec, USA) according to the manufacturer’s recommendations. Autologous monocytes were isolated by magnetic beads separation (MagniSort™ Human CD14 Positive Selection Kit; Thermo Fisher Scientific, USA) according to the manufacturer’s recommendations. Autologous memory CD4^+^ T cells and monocytes were mixed at a 1:1 ratio, and viral-specific T cells were re-stimulated through the addition of a pool of selected peptides from cytomegalovirus, Epstein–Barr virus, influenza and tetanus toxin (CEFT) (0.1 µg/mL of each peptide; Axxora, USA). Various concentrations of therapeutic antibodies were added, and after 7 days of culture, cytokine levels were assessed in the culture supernatant using ProcartaPlex immunoassay according to the manufacturer’s recommendations (Th1/Th2/Th9/Th17/Th22/Treg Cytokine 18-Plex Human ProcartaPlex™ Panel; Thermo Fisher Scientific). Results were expressed as the fold change in cytokine secretion relative to the level secreted in the absence of therapeutic antibodies.

### Regulatory T cell suppression assay

Human PBMCs were isolated from the peripheral blood of healthy donors by standard density gradient centrifugation on Ficoll-Hypaque (GE Healthcare-Life Sciences, USA). Regulatory T cells (Tregs) were isolated from PBMCs using magnetic beads technology (CD4^+^ CD25^+^ CD127^dim/-^ Regulatory T Cell Isolation Kit II; Miltenyi Biotec, USA) according to the manufacturer’s recommendations. Autologous PBMCs were stimulated with soluble anti-CD3 and anti-CD28 antibodies (1 and 0.5 µg/mL respectively; BD Biosciences, USA) in the presence of Tregs (1:1 PBMC to Treg ratio). The therapeutic antibodies were added, and after 3 days of culture, cytokine levels were measured in the culture supernatant using ProcartaPlex immunoassay according to the manufacturer’s recommendations (Th1/Th2/Th9/Th17/Th22/Treg Cytokine 18-Plex Human ProcartaPlex™ Panel; Thermo Fisher Scientific). Results were expressed as a percentage of inhibition of cytokine secretion relative to the level secreted in the absence of Tregs.

### M2 suppression

PBMCs were isolated from fresh blood collected from five healthy donors (Tissue Solutions, Glasgow, UK). Magnetic cell sorting was used to isolate monocytes by negative selection (without CD16 depletion; #19058, STEMCELL Technologies, Cambridge UK). A subset of PBMCs from each of the five donors was also cryopreserved for use later in the assay for PBMC/M2 co-culture. M2 macrophages were generated by culturing isolated monocytes with 50 ng/mL M-CSF (Peprotech, London UK) in RPMI-10 (RPMI-1640, 10% heat-inactivated FBS, 100 U/mL penicillin, 100 μg/mL streptomycin, and 2 mM L-glutamine, 50 μM β-mercaptoethanol) for 8 days in 96-well round-bottom plates. During the culture period, cells were replenished with fresh RPMI-10 supplemented with M-CSF on days 3 and 6. On day 8, medium was removed, fresh medium (without M-CSF) was added and the cells were activated with 0.1 µg/mL LPS (*E. coli*, #L2654, Sigma Aldrich) for 2 h. The macrophages were then washed to remove LPS and replenished with fresh media (without M-CSF). The M2 macrophages were co-cultured with autologous PBMCs (thawed and washed) at a 4:1 ratio (PBMC:M2) in the presence or absence of test antibodies or isotype controls (10 µg/mL), in triplicate. After 24 h of crosstalk, the cultures were stimulated with 1 µg/mL anti-CD3 (OKT3, #317315, BioLegend) and 2 µg/mL anti-CD28 (CD28.2 #302923, BioLegend) for three days to activate T cells via the TCR receptor complex. IFNγ was then measured in culture supernatant by IFNγ ELISA, (#88-7316-77, Life Technologies) with supernatants diluted 1:10 or 1:20 in the appropriate ELISA diluent to bring values within the detection range of the kits. Statistical analyses compared the results obtained with test antibodies and appropriate isotype control antibodies. This involved either a ratio paired *t*-test, or a one-way-ANOVA for multiple comparisons. For multiple comparisons, a post hoc Dunnett’s test was applied when comparing a control with multiple groups, and a Holm-Sidak method was used for comparisons between all groups. Statistical significance was assumed for *P* < 0.05.

### CD8+ T cell priming assay

The priming assay is based on a previously described protocol^68^. Briefly, DCs were generated from plastic-adherent human monocytes isolated from PBMCs of HLA-A2 + healthy donors. After 72 h of culture in GM-CSF/IL4-containing CellGro DC medium (CellGenix, cat. no. 20801-0500) with 1% human serum (HS), DCs were matured in medium containing 10 ng/mL IL4, 800 IU/mL GM-CSF, 10 ng/mL LPS, and 100 IU/mL IFNγ. Mature DCs were cultured in the presence or absence of 2.5 µg/mL Melan-A antigen peptide (ELAGIGILTV, JPT, cat. no. SP-MHCI-0006) for 16 h. The cells were then irradiated (30 Gy) and washed. Next, autologous naive CD8+ T cells were isolated from PBMCs with the EasySEP Human Naïve CD8+ T Cell Isolation Kit (Stem cell Technologies, cat. no. 19258) and co-incubated with mature DCs at a 4:1 ratio in CellGro DC medium with 5% HS and 30 ng/mL IL-21. Test antibodies at 10 µg/mL were added on day 0 and day 3 of co-culture. Fresh medium containing 5 ng/mL IL-7 and 5 ng/mL IL-15 was added on days 3 and 5. On day 10 of co-culture, T cells were harvested, counted, and stained with CD8, CD45RA, and CCR7 antibodies and ELAGIGILTV Dextramer (Immudex, cat. no. WB2162-APC) and analyzed by FACS.

### Ex vivo human primary endometrial tumor samples

Surgically resected primary endometrial tumors (Avaden Biosciences) were dissociated into a single-cell suspension using a Human Tumor Dissociation Kit (Miltenyi Biotec) according to manufacturer’s instructions and analyzed by flow cytometry for immune infiltration and surface marker expression. Cells were placed into 96-well round-bottom plates and incubated with 10 μg/ml test antibody in the presence of 0.01 μg/ml soluble anti-CD3 antibody (Clone Hit3a) for 6 days. IFNγ levels in the supernatant were measured by V-PLEX Human IFNγ Kit (Meso Scale Discovery).

### Co-culture of NY-ESO-1-specific T cells with A549-A2-ESO-1 tumor cells

NY-ESO-1-specific T cells were prepared according to^37^. The A549-A2-ESO-1 tumor cells were co-cultured with NY-ESO-1-specific T cells in RPMI 1640 medium (Gibco, cat. no. 11875-085) supplemented with 10% heat-inactivated FBS (HyClone, cat. no. SH30071.03). A549-NY-ESO-1 cells were added to 96-well flat-bottom plates, followed by NY-ESO-1-specific Ly95 T cells at a ratio of 1:1. Cells were co-cultured for 72 h at 37 °C with or without antibody treatment, whereby MCLA-145 was compared with atezolizumab, urelumab* or an equimolar mix of atezolizumab and urelumab* (all antibodies at 10 µg/mL final concentration). After 72 h, supernatants from co-cultures were collected and analyzed for IFNγ secretion using an IFNγ Quantikine ELISA Kit (R&D Systems, cat. no. DIF50) according to the manufacturer’s instructions.

### PD-L1 density analysis

Three CHO-PD-L1 stable cell lines were selected that expressed PD-L1 at levels similar to those of human tumor cell lines expressing relatively high (ES-2 cells), intermediate (MDA-MB231), and low (BxPC-3) levels of PD-L1. The PD-L1 cell surface density on these cells was analyzed by quantitative flow cytometry (Qifikit, DAKO) in accordance with the manufacturer’s instructions. Saturating amounts of anti-PD-L1 antibody (clone M1H1, BD, c#557924) or isotype control IgG1 were used to stain the tumor cell lines and the stable CHO-PD-L1 cell lines, followed by goat anti-mouse IgG FITC staining (DAKO). Data were acquired using a FACSCalibur, and the data were analyzed according to the Qifikit instructions.

### FACS-based CD137 ligand-blocking assay

huCD137L with an N-terminal FLAG tag (AdipoGen, cat. no. AG-40A-0198T) was added to CHO cells expressing huCD137 at 0.06 µg/mL together with threefold serial dilutions of each test antibody (25–0.034 µg/mL). Incubation was for 60 min on ice in the dark. Bound huCD137L-FLAG was detected using 1 µg/mL biotin-conjugated anti-huCD137L (R&D Systems, cat. no. BAF2295) and 1:200 streptavidin-PE. PE staining was detected with flow cytometry using mean fluorescence intensity (MFI) as readout.

### Flow cytometric analysis of human primary tumor samples

Endometrial tumor samples were obtained from Avaden Biosciences (Seattle WA). Every sample was actively consented in an IRB-approved research protocol at a US-based CLIA/CAP-accredited laboratory. Surgically resected endometrial tumors were dissociated into a single-cell suspension as described above. Tumor samples were then stained with an 18-color flow cytometry phenotyping panel. Live cells were distinguished from dead cells using (Biolegend, cat. no. 423110). T cells were identified by first isolating CD45+ cells (BD Biosciences, cat. no. 560178), then excluding CD14+ cells (Thermo Fisher Scientific, cat. no. 15-0149-42) and CD19+ cells (Biolegend, cat. no. 302210), and then selecting CD3+ cells (BD Biosciences, cat. no. 563546). T cells were then separated into CD4+ cells (BD Biosciences, cat. no. 564305) and CD8+ cells (BD Biosciences, cat. no. 564804). CD4+ cells were further separated into Treg cells by double-positive FoxP3 staining (Thermo Fisher Scientific, cat. no. 25-4777-42) and CD25+ cells (BD Biosciences, cat. no. 563159). The CD4+ Treg−, Treg+ and CD8+ T cell subsets were then classified by their positive expression of CD226 (Biolegend, cat. no. 338330), ICOS (Thermo Fisher Scientific, cat. no. 62-9948-42), CTLA-4 (BD Biosciences, cat. no. 555853), CD137 (BD Biosciences, cat. no. 745256), OX40 (Biolegend, cat. no. 350018), Lag-3 (Biolegend, cat. no. 369312), Tim-3 (BD Biosciences, cat. no. 565564), IL-10 (Biolegend, cat. no. 501411), GITR (BD Biosciences, cat. no. 747661), and PD-L1 (BD Biosciences, cat. no. 565188). Stained samples were run on an LSR Fortessa X-20 cell analyzer (BD Biosciences) and gates drawn using fluorescence minus one (FMO) controls.

### Flow cytometric analysis of TILs in MDA-MB-231 tumors

Upon termination of the in vivo phase of the study, 31 days after the start of treatment, tumor cells were analyzed by flow cytometric analysis. To this end, tumors were harvested and transferred into 15 mL conical tubes (Corning) containing 3 mL RPMI 1640 medium with 10% fetal calf serum. To obtain a single-cell suspension for flow cytometric analysis, tumors were micro-dissected, and digested using a Tumor Dissociation Kit (Miltenyi Biotec) according to the manufacturer’s instructions. Tumor cells were analyzed using viability dye for live/dead cells and fluorochrome-conjugated antibodies against human CD45 as a leukocyte marker. Expression of T cell surface markers was analyzed using fluorochrome-conjugated antibodies against CD3 (BD Biosciences, cat. no. 564307), CD8 (BD Biosciences, cat. no. 557834) and CD4 (BD Biosciences, cat. no. 557852). Analysis of expression of PD-L1 on monocytes used fluorochrome-conjugated antibodies against CD11b (BD Biosciences; Cat. # 564443) and PD-L1 (Biolegend; Cat. # 329706). Cells were run and analyzed using a BD LSR Fortessa flow cytometry analyzer and the FlowJo software package.

### Flow cytometric analysis of TILs from the Ly95-NY-ESO adoptive T cell transfer model

Upon termination of the in vivo phase of the study, 28 days after the start of treatment, TILs were analyzed by flow cytometric analysis. To this end, tumors were harvested, micro-dissected, and digested using a Tumor Dissociation Kit (Miltenyi Biotec) according to the manufacturer’s instructions. Following red blood cell lysis, cells were stained for FACS analysis using a viability dye and marker-specific fluorochrome-conjugated antibodies. Cells were run and analyzed using a BD LSR Fortessa flow cytometry analyzer and the FlowJo software package. Living TILs were identified as staining negative for viability dye and positive for CD45 (BD Biosciences, cat. no. 555483) and CD3-specific antibodies (BD Biosciences, cat. no. 564307). To identify NY-ESO-1-specific T cells, the T cell fraction was then characterized for expression of CD4 (BD Biosciences, cat. no. 557852), CD8 (BD Biosciences, cat. no. 557834), and GITR (eBiosciences, cat. no. 46-5875-42), as well as Vβ13.1 TCR chain (Miltenyi Biotec, cat. no. 130-108-742).

### Receptor proximity assay (VeraTag)

The VeraTag technology (Monogram) is based on a fluorescent antibody-based proximity assay and can be used to quantify protein-protein interactions in tissue sections or sections of densely packed cell pellets^42^. When target proteins are in proximity, a fluorescent tag is released into illumination buffer that can then be collected from the glass slide used in the assay and quantified using capillary electrophoresis. Each sample contains internal controls for standardization. Since many cells are required to generate formalin-fixed cell pellets for the VeraTag assay, cells were grown in suspension in large volumes and centrifuged. Cell pellets from the transactivation assay were prepared as follows: T75 flasks were coated overnight with 2 μg/mL anti-CD3 (clone OKT3, eBioscience, cat no 16-0037-85) in PBS. Next, Jurkat T cells expressing CD137 (Jurkat_CD137K) were added to at a concentration of 1.8 × 10^6^ cells/mL in 50 mL medium (9% FBS-HI RPMI 1640, 2 mM L-Glutamine) and incubated for 4 h at 37 °C. For the transactivation assay, the Jurkat cells were then co-cultured with CHO-K1 cells expressing PD-L1 (CHO-PD-L1) at a concentration of 0.45 × 10^6^ cells/mL in 50 mL medium (Jurkat to CHO). The cells were allowed to interact for 4 h, then MCLA-145 or reference/control antibody (10 µg/mL) was added for a further 2 h. The cells were then collected from the flasks by resuspension and scraping and fixed as follows: following centrifugation for 10 min at 290 × *g*, 4 °C, the medium was poured off and the cell pellet loosened and resuspended in ice-cold PBS. Centrifugation was repeated twice whereby the PBS was poured off and the cell pellet was loosened by vortexing. After the second wash, the pellet was resuspended in 30 mL 10% neutral buffered formalin (10% NBF, catalog number 5701, Thermo Fisher Scientific) and rotated gently overnight at 4 °C. After centrifugation for 10 min at 453 × *g*, 4 °C, the formalin was removed and the cell pellet resuspended in 80% ethanol at 25 × 10^6^ cells/mL and stored at 4 °C before processing as described previously^42^. For the various VeraTag assays cell pellets were prepared as follows: 4.5 × 10^5^ cells were placed on positively charged glass slides (Thermo Fisher Scientific), antigen retrieval was performed via heat using a pressure cooker (Biocare Medical). Following antigen retrieval, antibody pairs were added, and the released fluorescent VeraTags either by DTT (total expression) or antibody pairs (proximity) were detected by capillary electrophoresis. The released VeraTags were normalized to sample buffer volume to give units of relative fluorescence per 4.5 × 10^5^ cells. CD137 total expression was measured by DTT release and anti-CD137 mouse mAb M127 (BD Pharmingen cat#552532) or anti-CD137 mouse mAb BBK2 (ThermoFisher cat#MS-621). A goat anti-mouse IgG secondary antibody (Jackson ImmunoResearch cat#115-005-146) conjugated to VeraTag was paired with the primary antibody. In the isotype control experiment, the CD137 antibody was replaced by mouse IgG (BD Pharmingen cat#554121). PD-L1 total expression was measured by DTT release and anti-PD-L1 rabbit mAb E1L3N (Cell Signaling Technology cat#13684) and Pepsin digest of Goat Anti-Rabbit IgG(H + L) (Southern Biotech cat#4052-01) labeled with a fluorescent VeraTag reporter via a disulfide bond. In the isotype control experiment, the PD-L1 antibody was replaced by rabbit IgG (Cell Signaling Technology cat#3900). CD137 proximity clustering was measured by two different primary antibodies: anti-CD137 mouse mAb M127 (BD Pharmingen cat#552532) and anti-CD137 mouse mAb BBK2 (ThermoFisher cat#MS-621). Equal concentrations of anti-CD137 antibodies were labeled with either a fluorescent VeraTag reporter or biotin. Similarly, for the second primary antibody, equal concentrations of anti-CD137 mouse mAb BBK2 (ThermoFisher) were labeled with either a fluorescent VeraTag reporter or biotin. The CD137–PD-L1 proximity interaction was measured by the proximity-dependent release of a VeraTag reporter from an anti-CD137 mouse monoclonal antibody (clone BBK2, Thermo Fisher Scientific cat#MS-621, ectodomain) paired with an anti-PD-L1 rabbit monoclonal antibody (E1L3N, Cell Signaling cat#13684, c-terminus) and a biotinylated goat F(ab′)2 anti-rabbit IgG secondary antibody (Rockland cat#611-101-122). In the isotype control experiment, the PD-L1 antibody was replaced by rabbit IgG (Cell Signaling cat#3900).

### Reporting summary

Further information on research design is available in the Nature Research Reporting Summary linked to this article.

## Supplementary information

Supplementary Information

Reporting Summary

## Data Availability

The data supporting the findings of this study are available within the article and its Supplementary Information files and from the corresponding authors upon reasonable request. Source data are provided with this paper.

## Source data

Source Data
