# Supplementary material for: A human CD137×PD-L1 bispecific antibody promotes anti-tumor immunity via context-dependent T cell costimulation and checkpoint blockade
Source: Nat Commun. 2021 Jul 21;12:4445. doi: 10.1038/s41467-021-24767-5 (PMC8295259; doi:10.1038/s41467-021-24767-5)
Supplement: Supplementary file 1 — Supplementary Information [file 41467_2021_24767_MOESM1_ESM.pdf]

A human CD137×PD-L1 bispecific antibody promotes anti-tumor immunity via context-dependent T cell costimulation and checkpoint blockade

SUPPLEMENTARY INFORMATION

Supplementary Figures

Figure S1

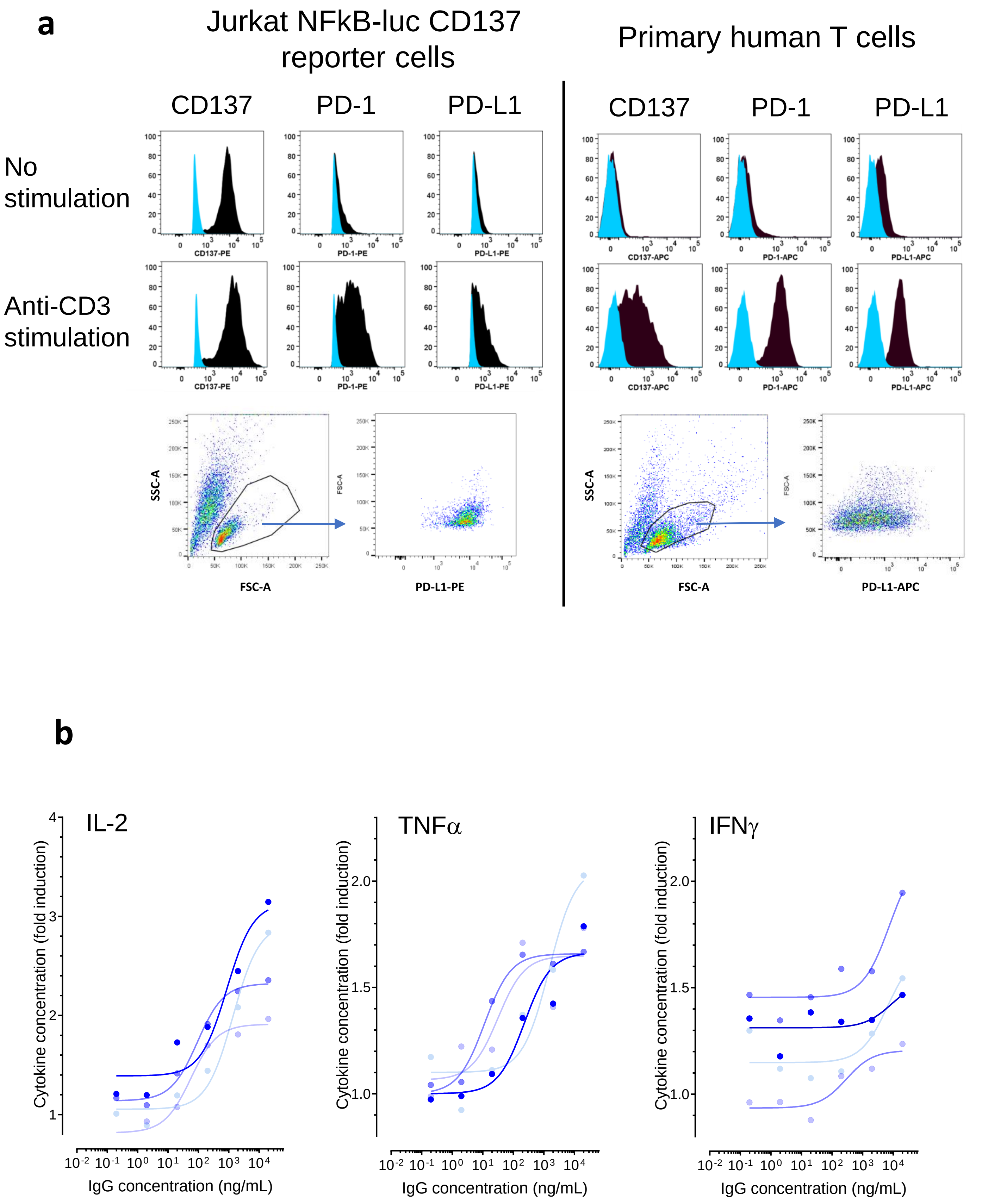
a
Jurkat NFkB-luc CD137 reporter cells
Primary human T cells
CD137
PD-1
PD-L1
No stimulation
Anti-CD3 stimulation
CD137-PE
PD-1-PE
PD-L1-PE
CD137-APC
PD-1-APC
PD-L1-APC
SSC-A
FSC-A
b
IL-2
TNFα
IFNγ
Cytokine concentration (fold induction)
IgG concentration (ng/mL)

Figure S1 cont

c

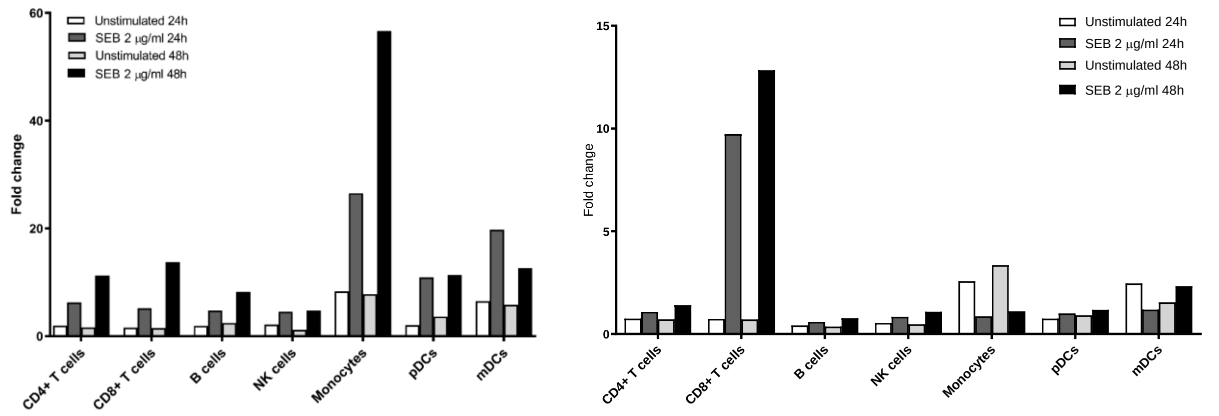

d

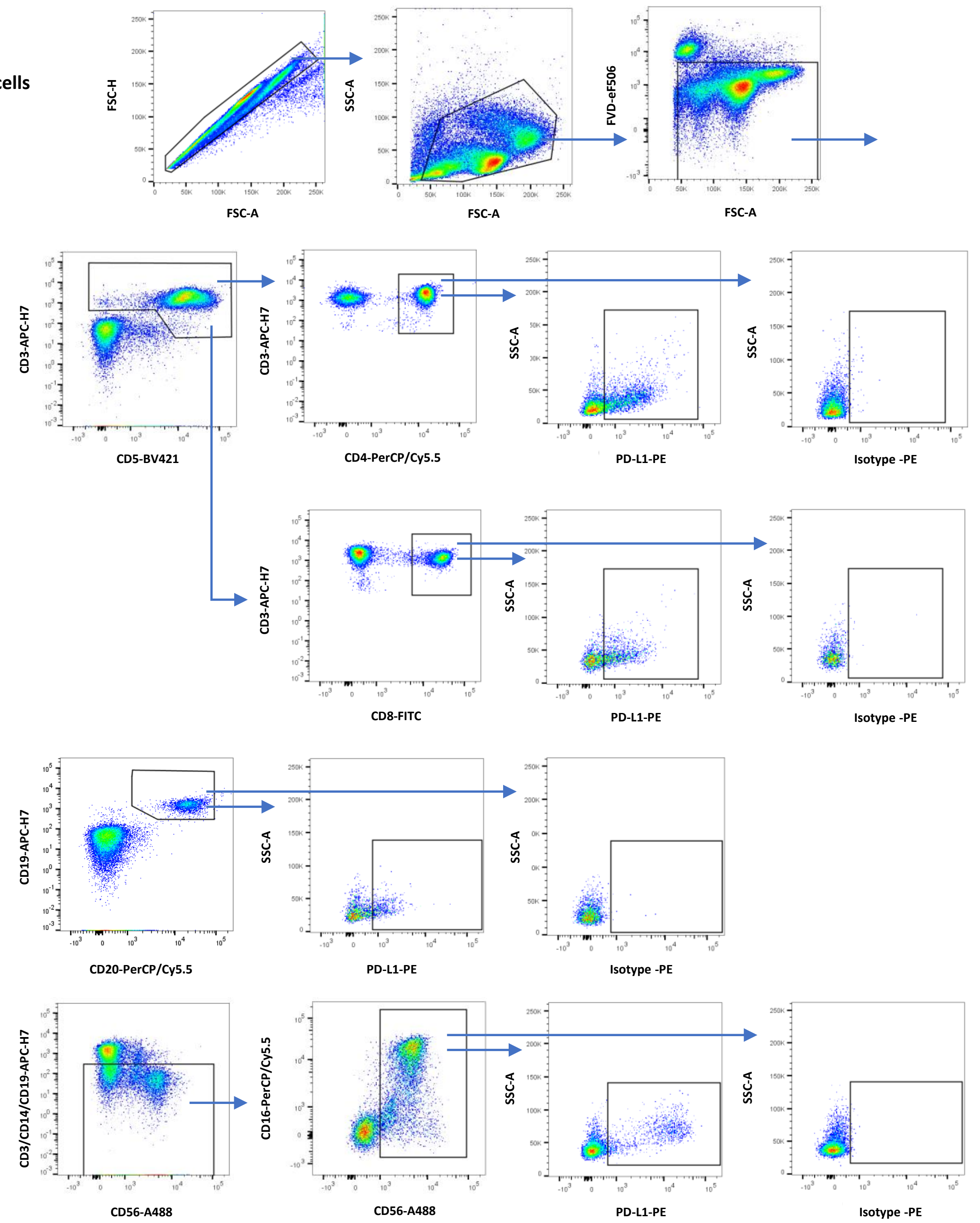

Figure S1 cont

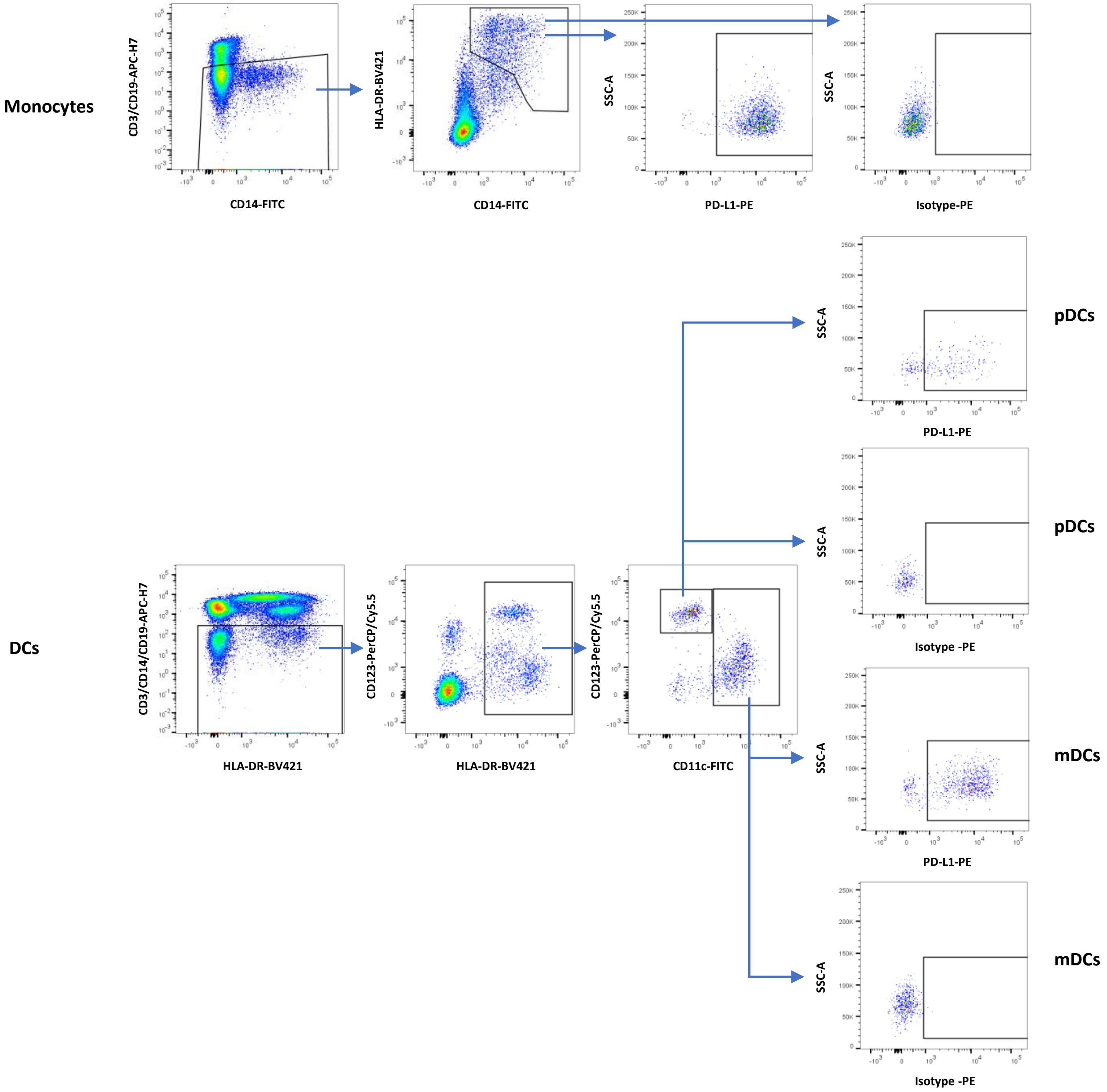

Supplementary figure 1. Identification of MCLA-145 using unbiased functional screening

(a) *top panels* Flow cytometry histograms that show upregulation of PD-1, PD-L1 and CD137 after CD3 stimulation on Jurkat reporter (left) and primary T cells (right). CD137, PD-1 and PD-L1 staining's are shown in black, isotype control staining's in blue, *lower panels* Examples of the gating strategy used to obtain Jurkat reporter and primary T cell histograms. (b) Fold change in IL-2, TNFα and IFNγ production by PBMC (n=3) stimulated with SEB in the presence of increasing concentrations of PD-L1 x CD137 bispecific antibodies (c) *top panels* Fold change in MFI of PD-L1 (Left, bar graph) and CD137 (Right, bar graph) staining after SEB stimulation of immune cells within the PBMC population as determined by flow cytometry. (d) Example of the flow cytometry gating strategy of SEB-stimulated PBMC to determine PD-L1 expression levels within the PBMC population.

Figure S2

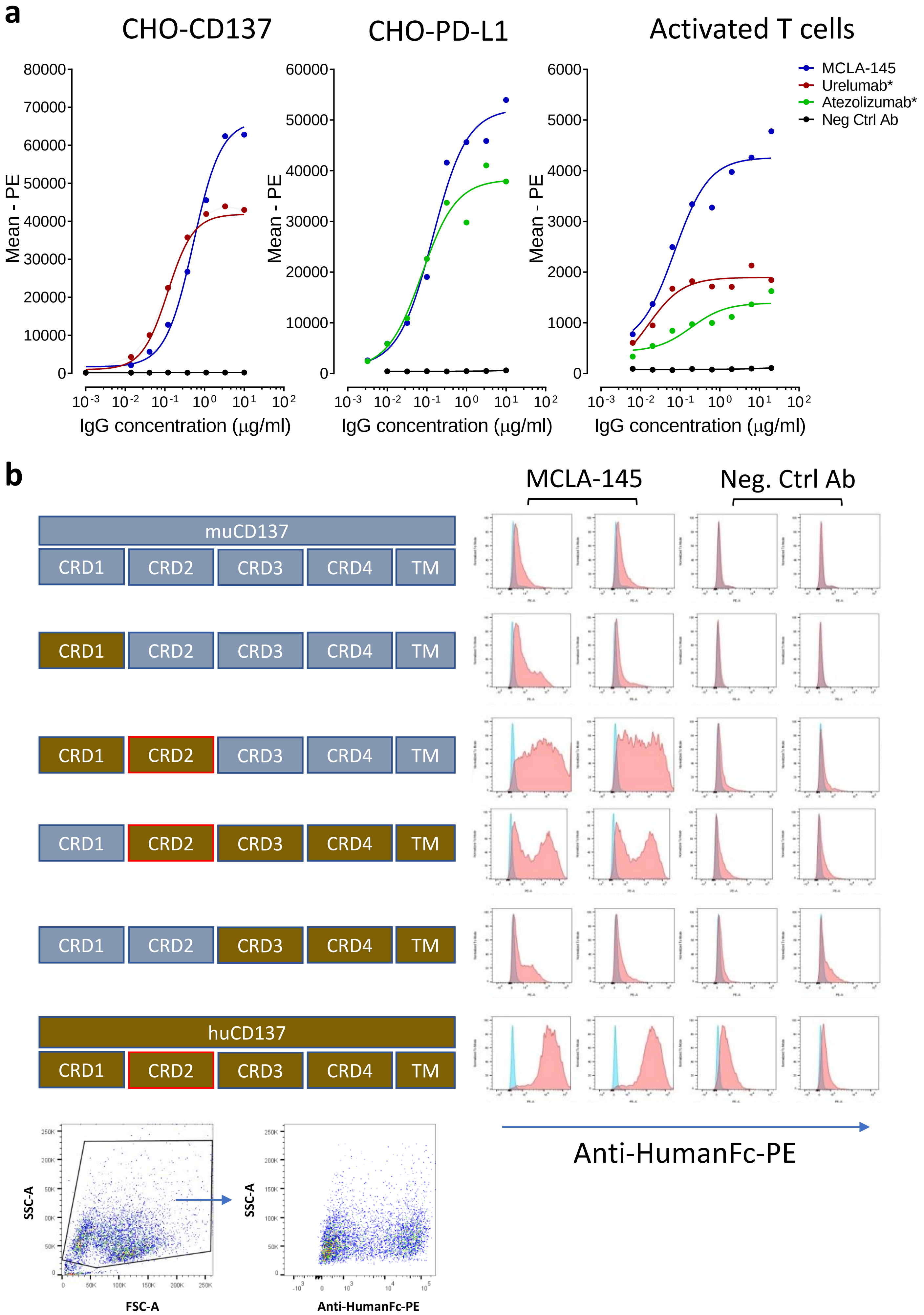
a
CHO-CD137
CHO-PD-L1
Activated T cells
MCLA-145
Urelumab*
Atezolizumab*
Neg Ctrl Ab
Mean - PE
IgG concentration (µg/ml)
b
MCLA-145
Neg. Ctrl Ab
muCD137
huCD137
CRD1
CRD2
CRD3
CRD4
TM
Anti-HumanFc-PE
SSC-A
FSC-A

Figure S2 cont

c

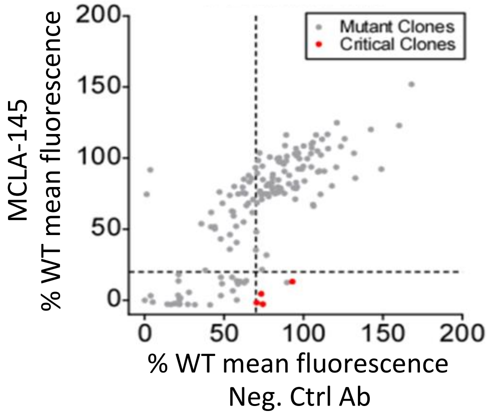

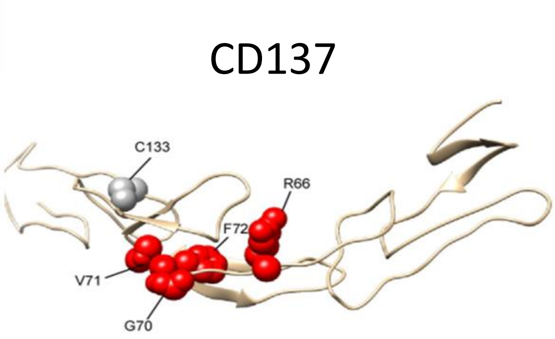

Supplementary figure 2. MCLA-145 exhibits high affinity for its targets and binds a distinct epitope on CD137

(a) Binding of MCLA-145 to CHO cells overexpressing CD137 (left), CHO-PD-L1 cells (middle) or activated T cells that express both receptors (right) as determined by flow cytometry; (b) *top left panel* schematic figure showing location of human and murine domains in chimeric CD137 expression constructs, *top right panel* Histograms showing MCLA-145 binding to HEK293 cells transfected with chimeric expression constructs, *lower panels* Example of flow cytometry gating strategy of HEK293 transfected cells; (c) *left panel* binding of MCLA-145 (y-axis) vs control CD137 mAb (x-axis) to members of a mutagenesis library of CD137, *right panel* location of residues critical for MCLA-145 binding shown in red on a ribbon structure of human CD137 6MGP.pdb.

Figure S3

a

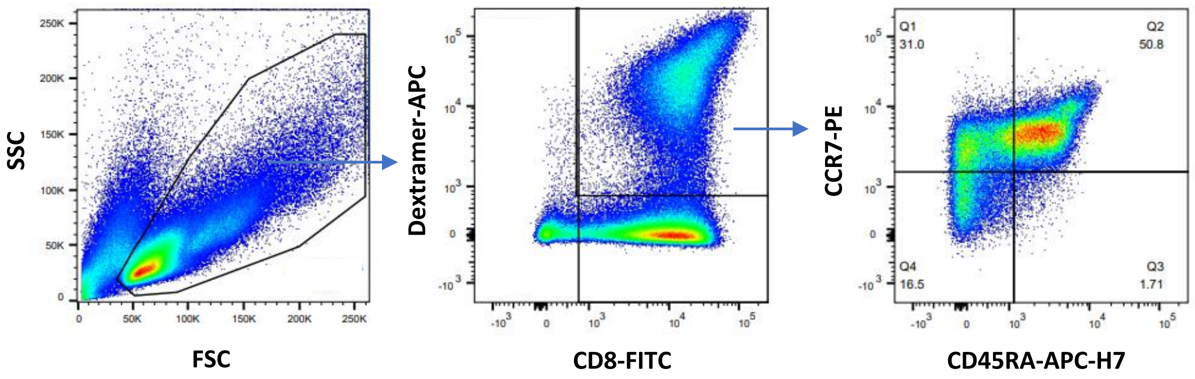

b

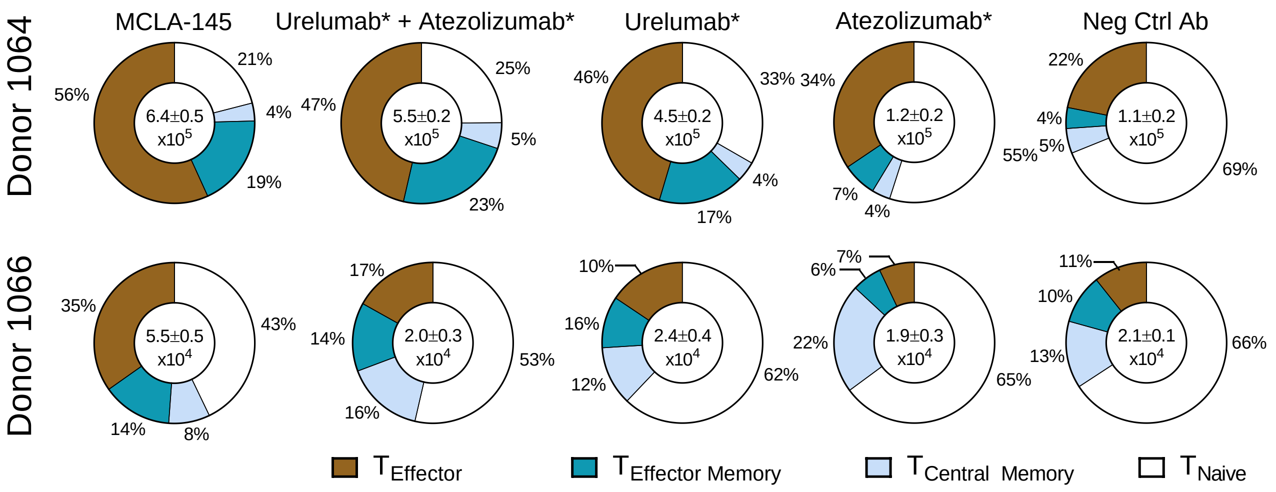

c

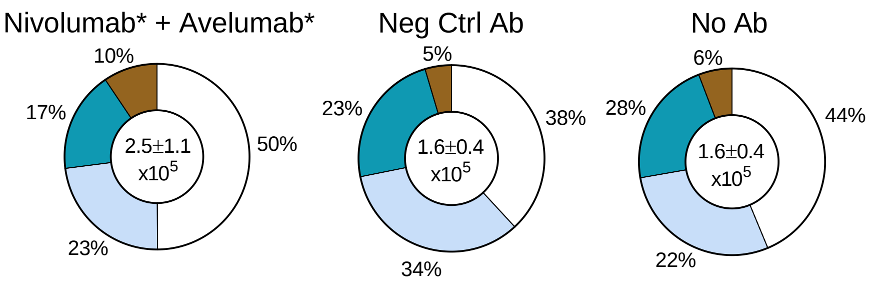

Supplementary figure 3. MCLA-145 potently activates T cells, promotes immunological memory and overcomes immunosuppression

Effect of PD-1 and PD-L1 antibodies on naïve CD8+ T cell priming. (a) Example (data from Figure 3a, main text and S3b and S3c) of day 10 of DC/T cell co-culture flow cytometry gating strategy to determine T naïve/memory stem cell (CD45RA+CCR7+), central memory T cell (CD45RA-CCR7+), effector memory T cell (CD45RA-CCR7-) and terminally differentiated effector T cell (CD45RA+CCR7-) frequency within the dextramer+ antigen-specific CD8+ T cell population; (b) Relative proportion of T cell subsets in colored segments and the absolute amount of CD8+dextramer+ antigen-specific T cells in the center of the donut from two donors on day 10 of DC/T cell co-culture after treatment with MCLA-145 or control antibodies as determined by flow cytometry; (c) Donut charts as described in (a) after treatment with combination of nivolumab* and avelumab* or control antibodies, average of two donors.

Figure S4

a

	PDL1 Percentages	
	**CD45+**	**CD45-**
**Tumor 1**	1.52	0.8
**Tumor 2**	0.19	0.39
**Tumor 3**	1.78	1.89
**Tumor 4**	2.13	0.46
**Tumor 5**	3.01	1.01

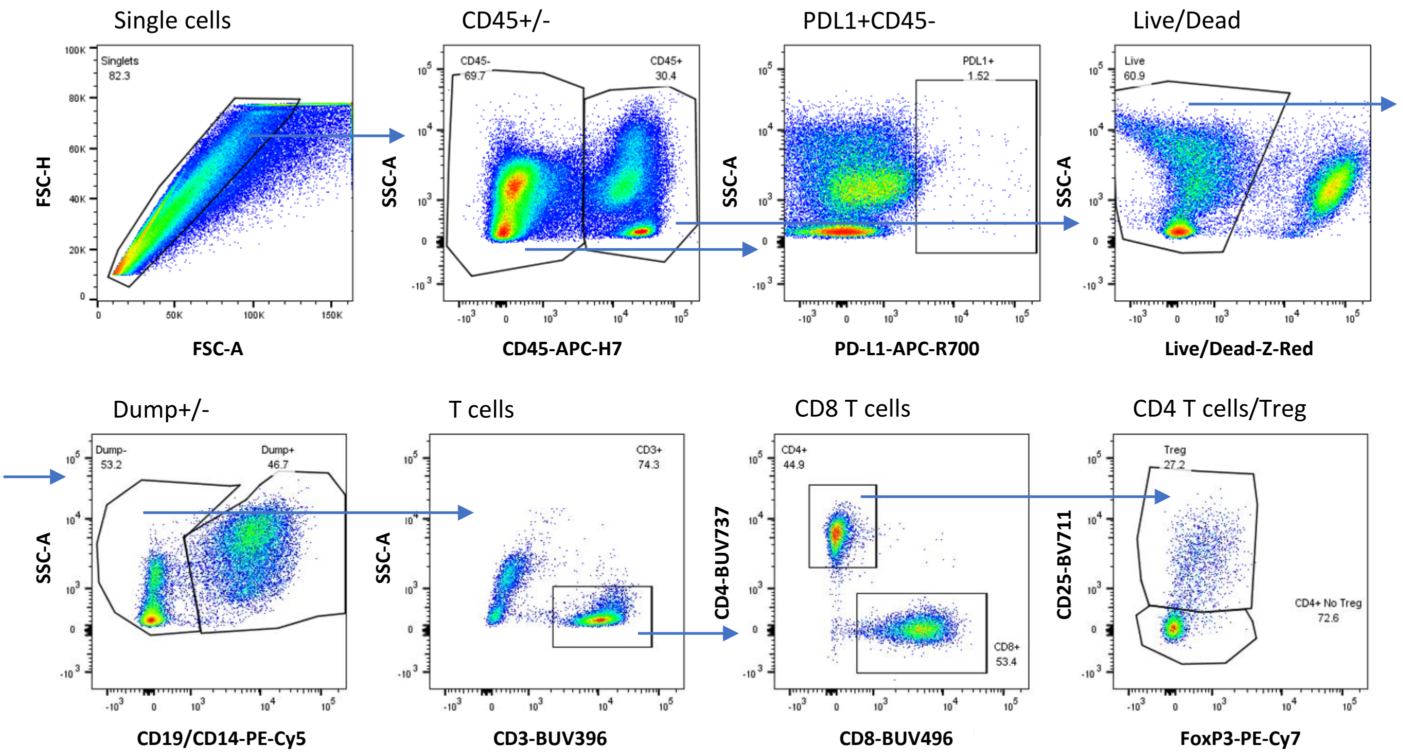

b

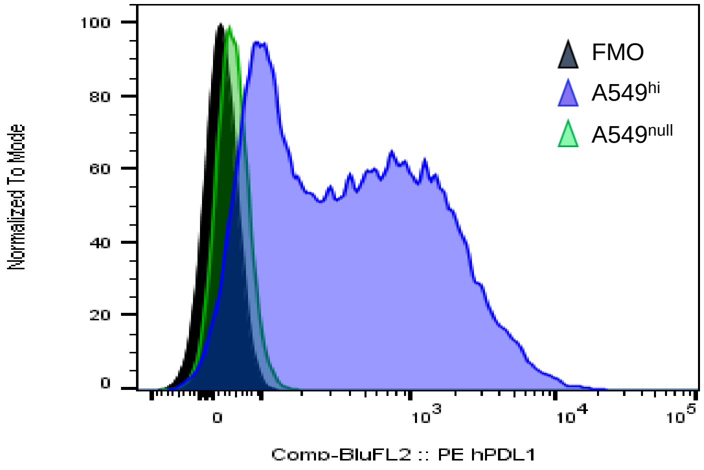

Figure S4 (cont)

c

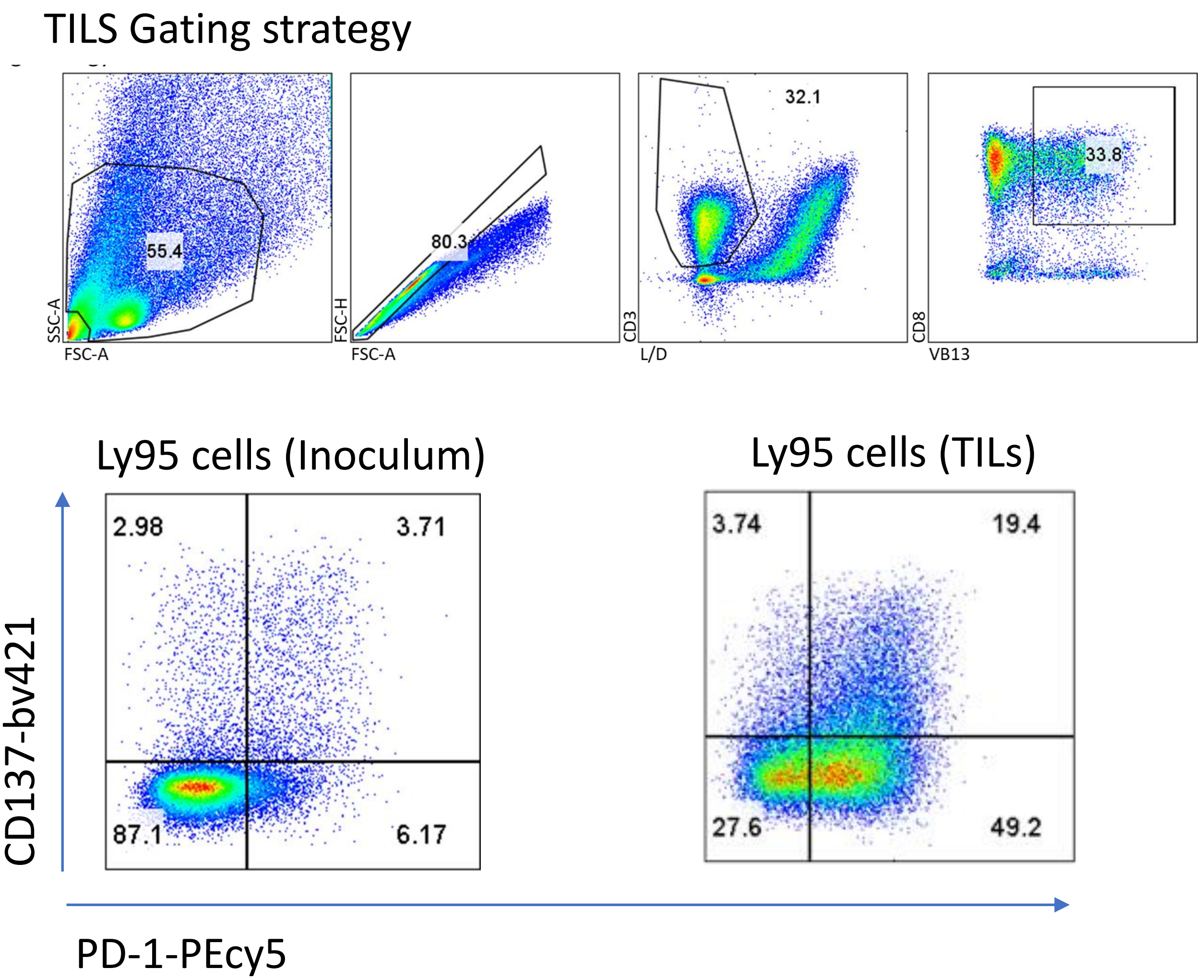

d

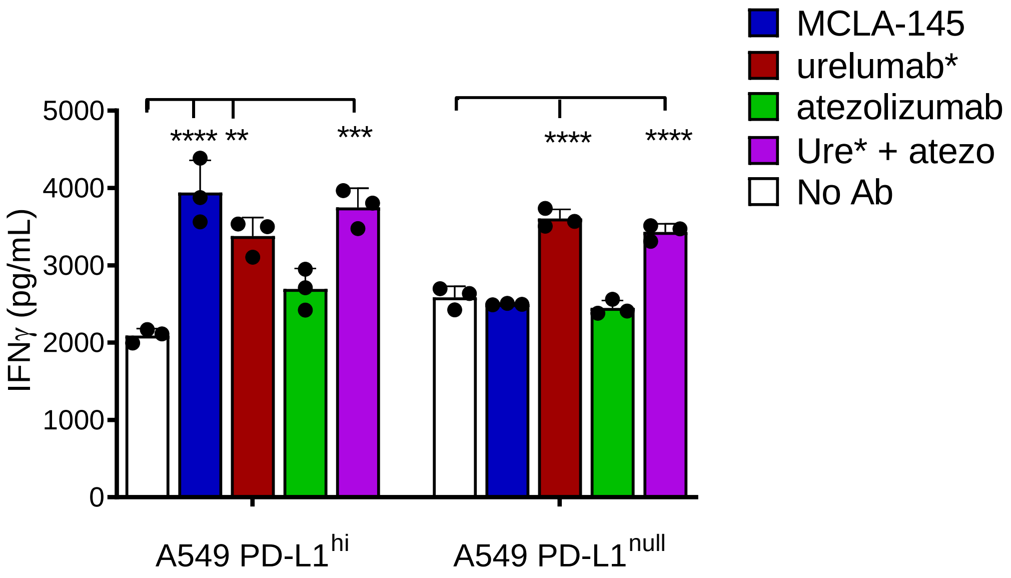

Figure S4 (cont)

e

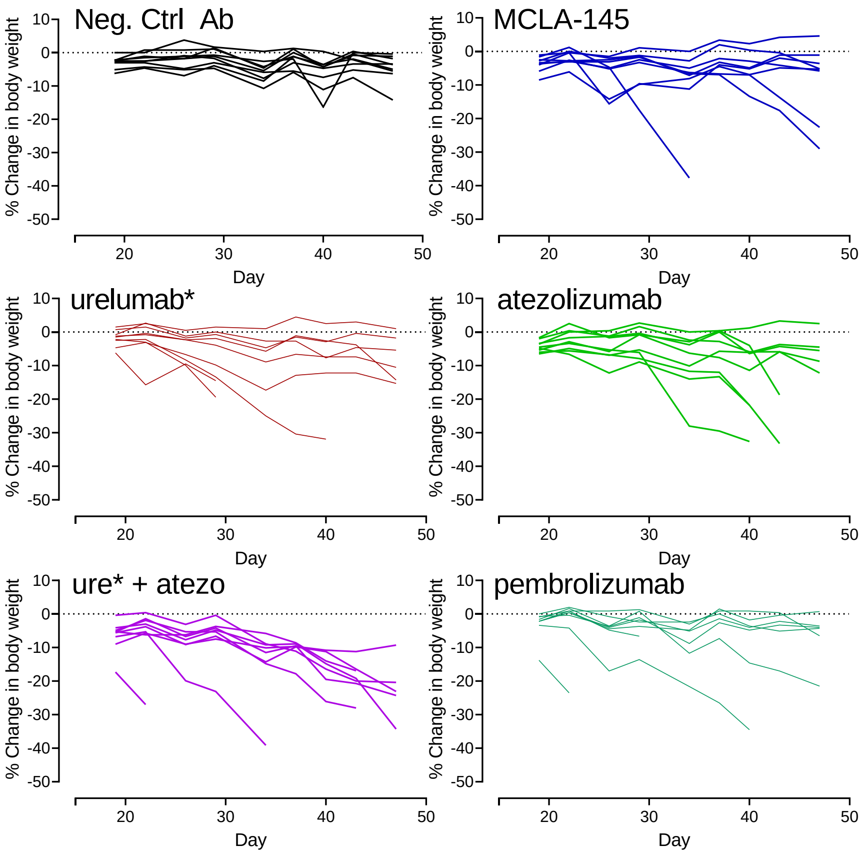

f

Compare column means (main column effect)					
Number of families	1				
Number of comparisons per family	21				
Alpha	0.05				
Bonferroni's multiple comparisons test	Mean Diff.	95.00% CI of diff.	Significant?	Summary	Adjusted P Value
Neg. Ctrl Ab vs. atezolizumab	3.26	0.2306 to 6.289	Yes	*	0.0236
Neg. Ctrl Ab vs. urelumab*	2.444	-0.5937 to 5.482	No	ns	0.2871
Neg. Ctrl Ab vs. ure* + atezo	8.322	4.957 to 11.69	Yes	****	<0.0001
Neg. Ctrl Ab vs. MCLA-145	2.062	-0.6204 to 4.744	No	ns	0.3888
Neg. Ctrl Ab vs. pembrolizumab	1.843	-1.220 to 4.906	No	ns	>0.9999
MCLA-145 vs. atezolizumab	-1.198	-4.857 to 2.461	No	ns	>0.9999
MCLA-145 vs. urelumab*	-0.3826	-4.047 to 3.282	No	ns	>0.9999
MCLA-145 vs. ure* + atezo	-6.261	-10.20 to -2.326	Yes	****	<0.0001
MCLA-145 vs. pembrolizumab	-0.2187	-3.903 to 3.465	No	ns	>0.9999

g

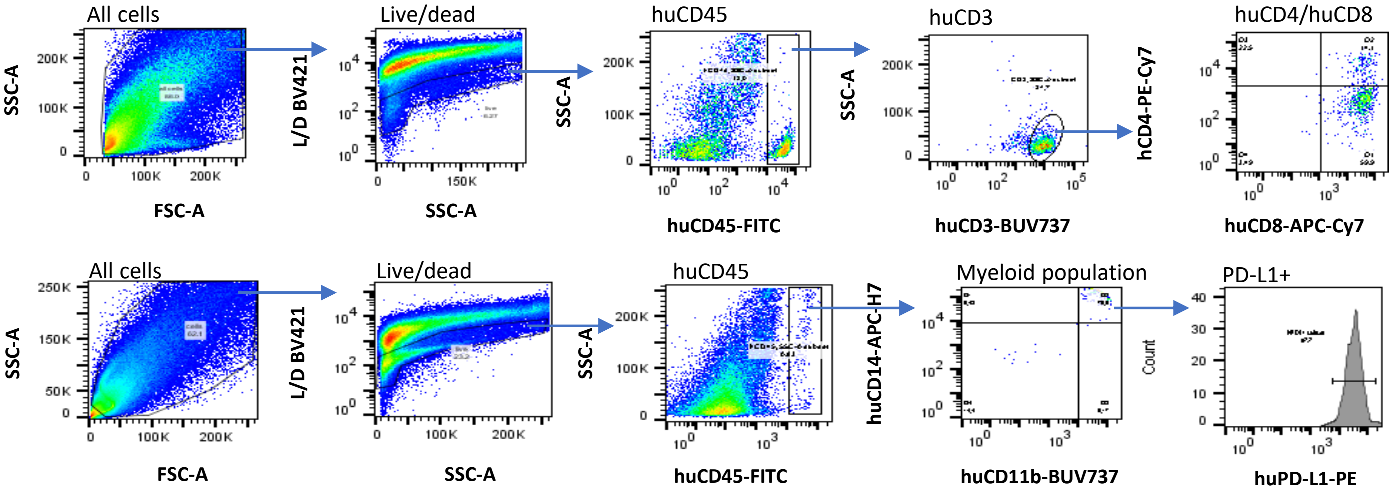

Supplementary figure 4. MCLA-145 recruits and activates T cells *ex vivo* and *in vivo* to enhance antitumor immunity

(a) *table* Percentage of PD-L1 positive cells in primary endometrial tumor explants as determined by flow analysis, *pseudocolor plots* example of gating strategy for flow cytometry to determine frequencies of endometrial tumor CD45- and CD45+ cells expressing PD-L1 and (data from Figure 4a, main text) T cell and T cell subset frequencies; (b) Flow analysis of PD-L1 expression of A549-PD-L1^{hi} and A549-PD-L1^{null} cells; (c) flow analysis of PD-1 and CD137 expression on Ly95 cells before inoculation and within tumor isolates (gating strategy for TIL isolates is illustrated); (d) Effect of MCLA-145 or reference antibodies on levels of IFNγ detected in supernatant of *in vitro* co-cultures of A549-PD-L1^{hi} or A549-PD-L1^{null} tumor cells and NY-ESO-1-specific T cells. Error bars represent SEM (n=6 independent experiments), **** $p<0.0001$ as determined by two-way ANOVA and Sidak's test; (e) The percentage weight change in individual human CD34+ engrafted NSG mice following subcutaneous inoculation with MDA-MB-231 cells and the indicated treatments (n=9 mice per group); (f) Table showing the results of a Bonferroni's multiple comparisons test after a two-way Anova. (g) Example (data from Figure 4G, main text) of MDA-MB-231 tumor flow cytometry gating strategy to determine percentages of CD4+ and CD8+ T cells and PD-L1 expressing monocytes within the human CD45+ cell population.

Figure S5

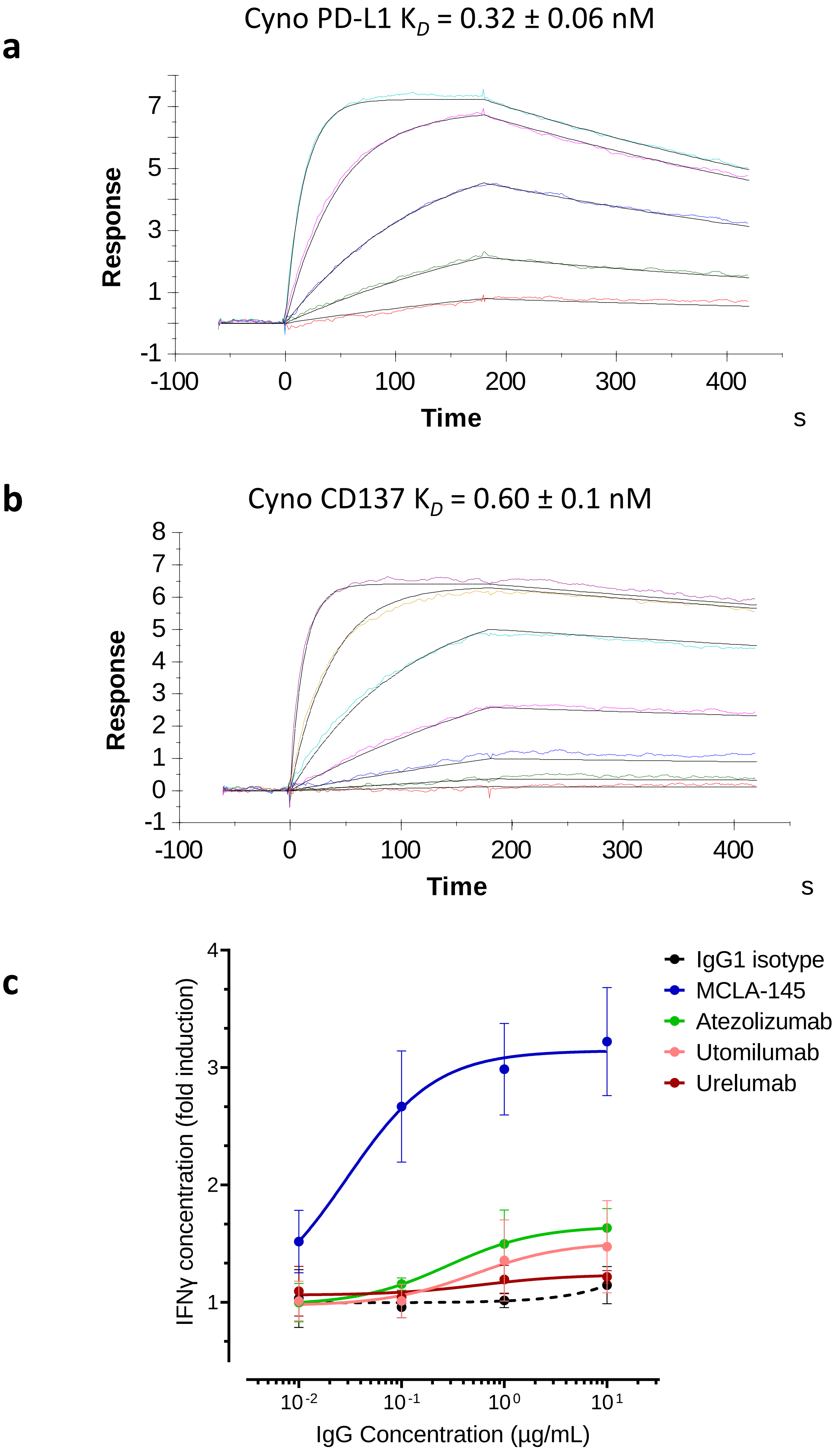

a
Cyno PD-L1 K_D = 0.32 ± 0.06 nM
Response
Time
s
-1
1
3
5
7
-100
0
100
200
300
400
b
Cyno CD137 K_D = 0.60 ± 0.1 nM
Response
Time
s
-1
0
1
2
3
4
5
6
7
8
-100
0
100
200
300
400
c
IFNγ concentration (fold induction)
IgG Concentration (µg/mL)
1
2
3
4
10^{-2}
10^{-1}
10^{0}
10^{1}
IgG1 isotype
MCLA-145
Atezolizumab
Utomilumab
Urelumab

Figure S5 (cont)

d

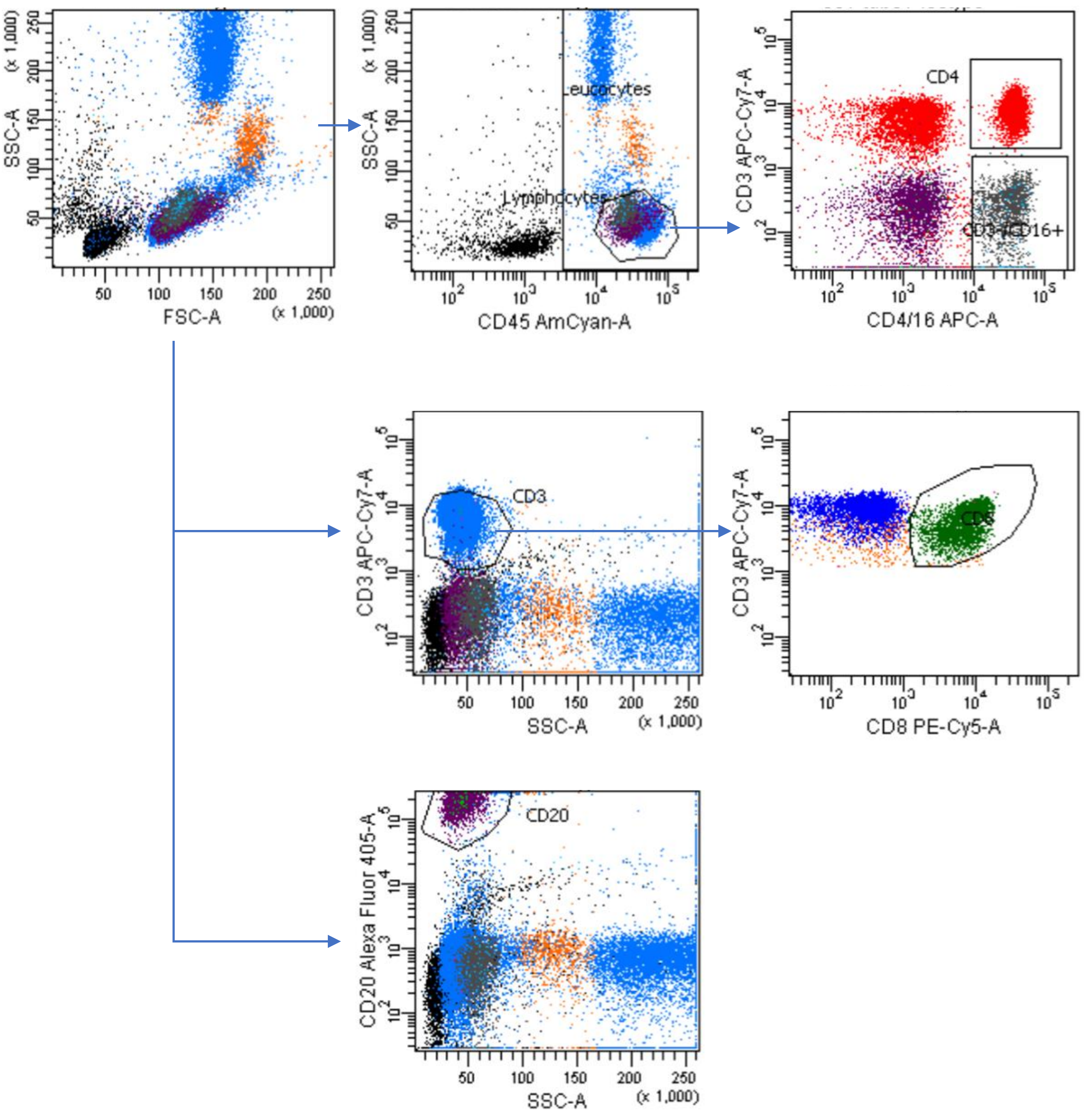

Supplementary figure 5. MCLA-145 is well tolerated in non-human primates at high doses

SPR Sensorgrams of cynomolgus PD-L1 (a) or cynomolgus CD137 (b) binding to MCLA-145, data are globally fitted to a 1 to 1 binding kinetics model with the calculated K_D indicated; (c) IFNγ production by cynomolgus monkey whole blood stimulated with SEB in the presence of various concentration of MCLA-145 (blue line), atezolizumab* (green line), urelumab (orange line), utomilumab* (red line), IgG1 isotype (black dashed line), or IgG4 isotype (gray dashed line) for 72 hours. Data represent the average fold change induction compared to blood stimulated in the absence of antibody (n = 4, error bars are mean ± SD statistical analysis was performed by ordinary one-way Anova and Dunnett's test, $p < 0.0001$ for Neg. Ctrl vs MCLA-145); (d) Example (data from Figure 5f, main text) of the gating strategy for flow cytometry to determine blood T (CD3+), CD4 T (CD45+, lymphocytes, CD3+/CD4+), CD8 (CD3+/CD8+), B (CD20+) and NK (CD45+, lymphocytes, CD3-/CD16+) cells.

Figure S6

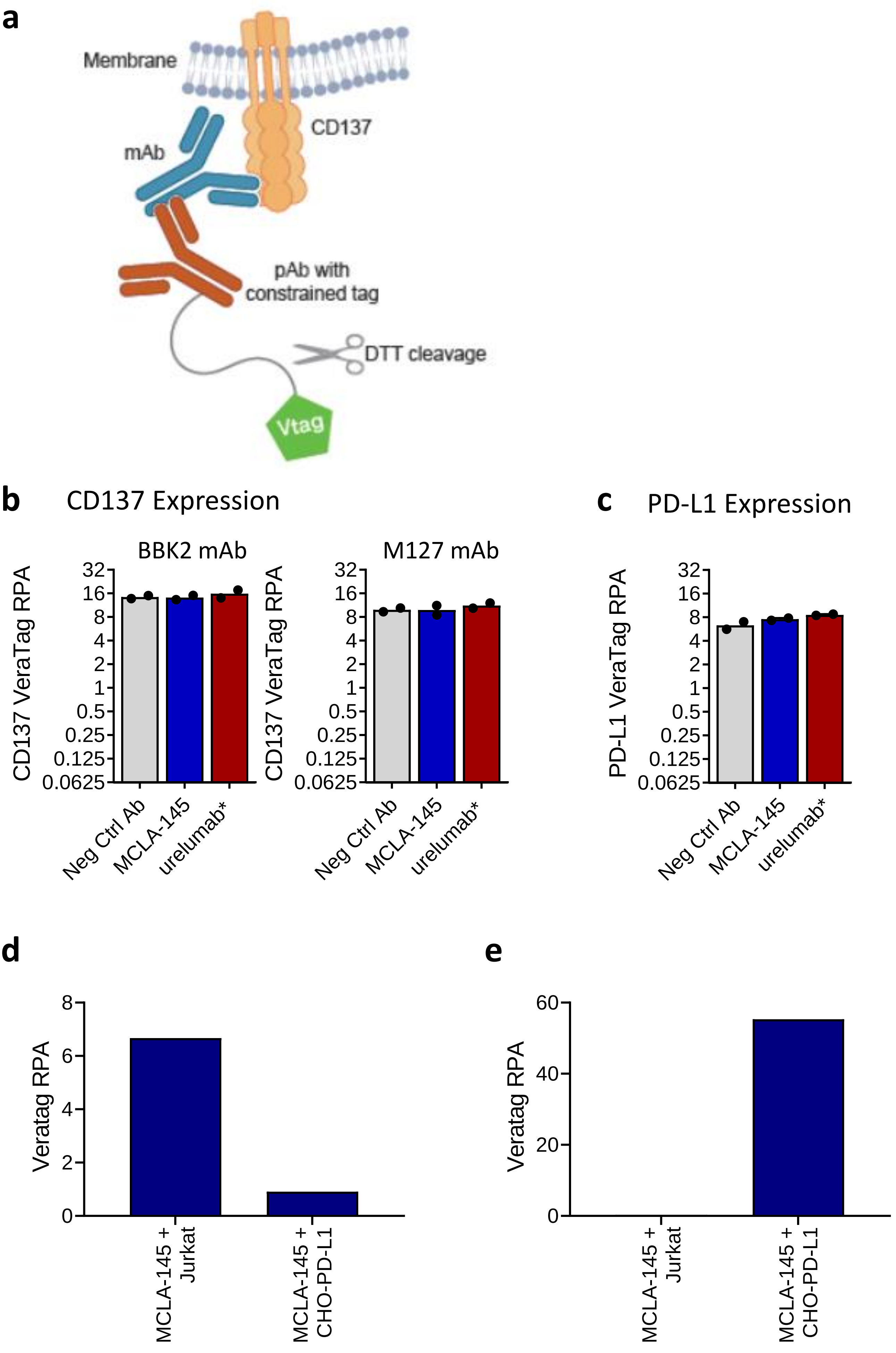
a
Membrane
CD137
mAb
pAb with constrained tag
DTT cleavage
Vtag
b
CD137 Expression
BBK2 mAb
M127 mAb
CD137 VeraTag RPA
32
16
8
4
2
1
0.5
0.25
0.125
0.0625
Neg Ctrl Ab
MCLA-145
urelumab*
c
PD-L1 Expression
PD-L1 VeraTag RPA
d
Veratag RPA
8
6
4
2
0
MCLA-145 + Jurkat
MCLA-145 + CHO-PD-L1
e
60
40
20
0

Figure S6 (cont)

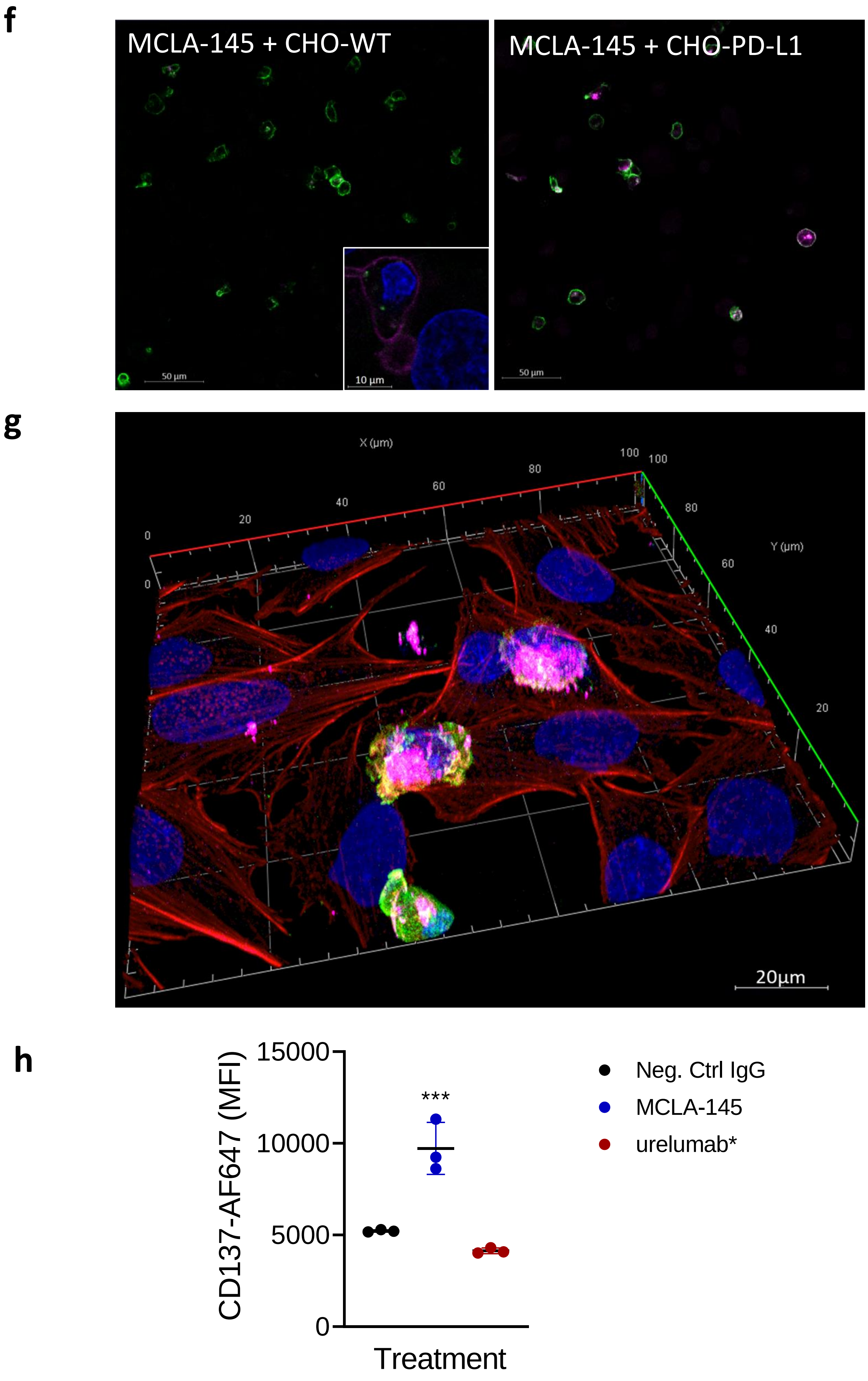

Supplementary figure 6. MCLA-145 transactivates CD137 in a PD-L1 dependent manner via receptor clustering

(a) Schematic of Vera Tag assay setup to detect expression; expression of CD137 using two different antibodies BBK2 and M127 (b), or PD-L1 (c) in co-cultures treated with MCLA-145 or control antibodies; expression of CD137 (d) or PD-L1 (e) in single cultures treated with MCLA-145; confocal images of CD137 receptor internalization in cocultures, (f) T cells and CHO-WT (upper panel) or CHO-PD-L1^{+} cells (lower panel) incubated with MCLA-145 Single plane view, magnification 20x (inset 63x); (g) 3D reconstruction of sequential confocal images of cells incubated with MCLA-145 and CHO-PD-L1^{+} cells, nuclear (DAPI) staining (blue), CD8 (green), CD137 (magenta), actin (red) Single plane view, magnification 20x (insets 63x); (h) using segmentation analysis, mean intensity of CD137-AF647 objects were measured as a subset of the CD8-AF488 positive cells, images from 3 separate microscopy fields were taken with 20X magnification per sample; error bars are mean ± SD statistical analysis was performed by Ordinary one-way Anova and Dunnett's test, *** p = 0.0003. Data is representative of two repeat experiments for figure 6f and 6g.

SUPPLEMENTARY TABLES

Supplementary Table 1 | Characteristics of cLC antibodies binding PD-L1 and CD137

FAB ID	Target	Super cluster[$]	Bin[#]	Domain[%]	Agonistic mAb[||]	Ligand blockade (%)[a]
001	CD137	20	A	1/2	X	44
002	CD137	20*	A	1/2	X	6
003	CD137	4	A	1 or 2	X	26
004	CD137	3*	A	1 or 2	-	-60
005	CD137	22*	B	1/2	-	50
006	CD137	19*	B	1/2	-	24
007	CD137	13*	B	1/2	-	-126
008	CD137	15*	C	4	-	24
009	CD137	73	D	ND	-	77
010	CD137	68	D	ND	-	32
011	CD137	43	E	1	-	44
012	CD137	10*	E	1	X	8
013	CD137	72	E	1	-	2
014	CD137	73*	E	1	-	-67
015	CD137	8*	E	1	-	-3
016	CD137	11	E	1	-	100
017	CD137	51*	E	1	-	32
018	CD137	57*	E	1	-	-76
019	**CD137**	**13**	**F**	**2**	**-**	**101**
020	**CD137**	**12**	**F**	**2**	**-**	**101**
021	CD137	50	F	2	X	72
022	CD137	27	F	2	X	99
023	CD137	15	F	2	-	95
024	CD137	35	F	2	-	96
025	CD137	52	F	2	-	24
026	**CD137**	**49**	**G**	**2/3**	**-**	**99**
027	CD137	31	G	2	X	89
028	CD137	19	G	2	X	67
029	CD137	8	G	2 or 3	X	84
030	CD137	17	G	2 or 3	-	58
031	CD137	1	G	2 or 3	-	-2
032	CD137	53	H	3	-	81

033	CD137	18	H	3	X	76
034	CD137	39	H	3	X	20
035	CD137	75*	I	4	-	-16
036	CD137	1*	J	ND	-	-22
037	CD137	6*	J	ND	X	-80
038	CD137	2*	J	ND	X	-76
039	CD137	14*	J	ND	-	-97
040	CD137	54*	J	ND	-	-43
041	CD137	69*	J	ND	-	-105
042	CD137	53*	J	ND	-	-64
043	CD137	65*	J	ND	-	-101
044	CD137	17*	J	ND	-	-71
045	**CD137**	**6**	**K**	**1/2**	**-**	**102**
046	CD137	63	K	1/2	X	55
047	CD137	24*	K	1/2	-	-100
048	PD-L1	16	NA	NA	NA	100
049	PD-L1	13	NA	NA	NA	100
050	**PD-L1**	**78**	**NA**	**NA**	**NA**	**100**
051	PD-L1	21	NA	NA	NA	100
052	PD-L1	2	NA	NA	NA	0
053	PD-L1	67	NA	NA	NA	100
054	PD-L1	66	NA	NA	NA	100
055	PD-L1	12	NA	NA	NA	100
056	PD-1	12	NA	NA	NA	100
057	PD-1	12	NA	NA	NA	100
058	PD-1	3	NA	NA	NA	0

[$] Supercluster, defined as VH sequences belonging to the same VH germ line gene based on cluster analysis, a HCDR3 (heavy chain complementarity determining region 3) of the same amino acid length and having 70% amino acid sequence identity in the HCDR3 region, each supercluster so defined is arbitrarily assigned a numerical ID; [#] Bin, arbitrary classification based on classes of binding profiles against chimeric CD137 constructs (note these were in some cases distributed over different domains); [%] Domain, CD137 domain to which antibody was mapped using mouse human swapped-domain constructs (1 or 2 means that the antibody could not be clearly mapped to one of the two domains); [||] Agonistic mAb, capacity of bivalent antibody to activate Jurkat-NFkB-luc-CD137; [a] Ligand blockade (%), capacity of Fab arm to block interaction with CD137L or

PD-1; most potent Fab arms in bispecific format indicated in bold;[*] Indicates FAB superclusters that are derived from selections with synthetic libraries; NA, Not applicable; ND, Not done.

Supplementary Table 2 | Summary of kinetic parameters determined by SPR for human PD-L1 and human CD137 binding to antibodies

Antibody	Target	Capture (RU[#])	Rmax thr[a]	Rmax.exp	k_a (1/Ms)	k_d (1/s)	K_D (nM)
MCLA-145	PD-L1	135	23	21	3.059E+6	0.001551	0.51
Parental mAb[b]	PD-L1	135	47	46	2.084E+6	0.001389	0.67
atezolizumab*	PD-L1	146	51	48	7.446E+5	4.372E-4	0.59
avelumab*	PD-L1	164	57	55	3.312E+5	3.592E-4	1.1
MCLA-145	CD137	115	14	16	8.732E+5	0.001679	1.9
Parental mAb[b]	CD137	99	24	27	1.038E+6	0.001121	1.1
utomilumab*	CD137	100	24	25	2.192E+6	0.01888	8.6
urelumab*	CD137	86	20	21	6.515E+5	0.002993	4.6

[#] Response Unit
[a] Theoretical Rmax (maximal response) calculated from capture level, MW, and stoichiometry
[b] Fab arm binding target from MCLA-145 reformatted as a bivalent IgG1 mAb

Supplementary Table 3 | Details of cell affinity measurements for radiolabeled MCLA-145

Cell Line	Ligand	Assay #	K_D (nM)	95% CI[#] (nM)	R^2	# of Cells
CHO-PD-L1	^{125}I-MCLA-145	1	0.48	0.26 to 0.70	0.97	4,000
CHO-PD-L1	^{125}I-MCLA-145	2	0.46	0.24 to 0.69	0.98	4,000
CHO-PD-L1	^{125}I-MCLA-145	3	0.52	0.21 to 0.83	0.99	4,000
		Mean ± SD	*0.49 ± 0.030*			
CHO-CD137	^{125}I-MCLA-145	1	2.05	0 to 5.35	0.87	16,000
CHO-CD137	^{125}I-MCLA-145	2	2.00	1.03 to 2.98	0.96	16,000
CHO-CD137	^{125}I-MCLA-145	3	2.02	0.28 to 3.75	0.98	16,000
		Mean ± SD	*2.02 ± 0.022*			

[#] Confidence interval

Supplementary Table 4 | Antibody information

Antibody	Catalogue number	Lot number	Dilution	Use
anti-huCD3 OKT3	16-0037-85	4303589	1: 4000	Assay
anti-huPD-L1 MIH1	14-5983-82	E04651-1630	1:500	FACS
anti-huCD137 M127	552532	7054673	1:500	Vera Tag
anti-huCD137 BBK2	MS-621	SI2448351L	1:200	Vera Tag
mouse IgG1, k isotype control antibody MOPC-21	401408	B189676	1:100	Vera Tag
anti-huPD-L1 E1L3N	1368		1: 200	Vera Tag
rabbit control antibody Rabbit (DA1E) mAb IgG XP® Isotype Control			1:2500	Vera Tag
biotin-conjugated anti-huCD137L BAF2295		UQI0116071	1:50	FACS
FITC anti-huCD8HIT8a	555634	524298	1:60	FACS
anti-huCD45ROUCLH1	555493	6320585	1:100	FACS
APC-H7 anti-huCD45RAHI100	560674	7047723	1:50	FACS
APC/Fire™ 750 anti-huCD62LDREG-56	304846	B222694	1:100	FACS
PE anti-huCCR7150503	FAB197P	LEV1415041	1:100	FACS
PE anti-huCD57TB01	12-0577-42	4292328	1:100	FACS
APC-H7 anti-huCD28CD28.2	561368	3031774	1:50	FACS
PE anti-huCD27M-T271	555441	7087984	1:100	FACS
AF488 anti-huCD56B159	561905	6057866	1:60	FACS
APC-H7 anti-huCD3SK7	560176	5255854	1:60	FACS
anti-huCD8SK1	345784	5335947	1:100	FACS
anti-huCD137 4B4-1	555955		1:100	FACS
anti-huPD-L1 130021	1561		1:100	FACS
anti-huCD3 OKT3	317315		1:1000	FACS
anti-huCD28 28.2	302923		1:1000	FACS
BV421 anti-huCD5UCHT2	562646	6049748	1:20	FACS
PerCP/Cy5.5 anti-huCD4 RPA-T4	560650	6209689	1:60	FACS
PerCP/Cy5.5 anti-huCD20 2H7	560736	6105587	1:20	FACS
APC-H7 anti-huCD19HIB19	560727	5281893	1:20	FACS
PerCP/Cy5.5 anti-huCD16 3G8	560717	6168980	1:60	FACS
APC-H7 anti-huCD14MΦP9	560180	526191	1:60	FACS
FITC anti-huCD11cB-ly6	561355	5128648	1:60	FACS
PerCP/Cy5.5 anti-	560904	6070695	1:20	FACS

huCD1237G3				
PE anti-huPD-L1MIH1	557924	5295791	1:20	FACS
PE Mouse IgG1, k isotype control antibody MOPC-21	556650	3042677	1:60	FACS
BV421 anti-HLA-DRG46-6	562805	6077504	1:20	FACS
APC anti-huCD1374B4-1	550890	5225539	1:20	FACS
APC Mouse IgG1, k isotype control antibody MOPC-21	400122	B210935	1:20	FACS
AF647 anti-huCD1374B4-1	309823		1:50	Confocal imaging
AF488 anti-huCD8 RPA-T8	557696		1:100	Confocal imaging
human IgG4 isotype control antibody QA16Al5	94242	8261576	1:100	FACS
BV510 anti-huCD226 11A8	338330	B259831	1:40	FACS
Super Bright 436 anti-ICOS ISA-3	62-9948-42	432838	1:160	FACS
PE anti-CTLA-4 BNI3	555853	7208849	1:40	FACS
BV605 anti-CD137 4b4-1	745256	8292716	1:40	FACS
AF647 anti-OX40 ACT35	350018	B245379	1:160	FACS
PerCP Cyanine5.5 anti-Lag-3 11C3C65	369312	B254045	1:80	FACS
BV650 anti-Tim-3 7D3	565564	8120836	1:80	FACS
AF488 anti-IL-10 JES3-9D7	501413	B189479	1: 10	FACS
BV786 anti-GITR V27-580	747661	8292722	1:40	FACS
APC-R700 anti huPD-L1 MIH1	565188	8092804	1:400	FACS
BUV737 anti-huCD3 UCHT1	564307	8101534	1:20	FACS
APC-Cy7 anti-huCD8 SK1	557834	8184768	1:20	FACS
PE-Cy7 anti-huCD4 SK3	557852	8317967	1:20	FACS
AF647 anti-huCCR7 3D12	557734	8194595	1:20	FACS
BUV737 anti-huCD11b M1/70	564443	7338572	1:20	FACS
PE anti-huPD-L129E.2A3	329706	B262098	1:20	FACS
BV 711™ anti-hu CD45RAHI100	304138	B250615	1:20	FACS
FITC anti-huCD45 HI30	555483		1:20	FACS
anti-Vβ13.1 TCR chain REA560	130-108-742		1:20	FACS
anti-huCD137 monoclonal antibody (utumilumab analog)				FACS, in vitro /in vivo assays
anti-huCD137 monoclonal				FACS, in vitro

antibody (urelumab analog)				/in vivo assays
anti-huPDL1 monoclonal antibody (atezolizumab analog)				FACS, in vitro /in vivo assays
anti-huPDL1 monoclonal antibody (atezolizumab)				FACS, in vitro /in vivo assays
anti-huPD-1 monoclonal (pembrolizumab)				FACS, in vitro /in vivo assays
