# Supplementary material for: A human CD137×PD-L1 bispecific antibody promotes anti-tumor immunity via context-dependent T cell costimulation and checkpoint blockade
Source: Nat Commun. 2021 Jul 21;12:4445. doi: 10.1038/s41467-021-24767-5 (PMC8295259; doi:10.1038/s41467-021-24767-5)
Supplement: Supplementary file 2 — Reporting Summary [file 41467_2021_24767_MOESM2_ESM.pdf]

nature research

Corresponding author(s): Mark Throsby
Last updated by author(s): Nov 21, 2020

Reporting Summary

Nature Research wishes to improve the reproducibility of the work that we publish. This form provides structure for consistency and transparency in reporting. For further information on Nature Research policies, see our Editorial Policies and the Editorial Policy Checklist.

Statistics

For all statistical analyses, confirm that the following items are present in the figure legend, table legend, main text, or Methods section.

n/a	Confirmed	
☐	☒	The exact sample size (*n*) for each experimental group/condition, given as a discrete number and unit of measurement
☐	☒	A statement on whether measurements were taken from distinct samples or whether the same sample was measured repeatedly
☐	☒	The statistical test(s) used AND whether they are one- or two-sided<br>*Only common tests should be described solely by name; describe more complex techniques in the Methods section.*
☒	☐	A description of all covariates tested
☐	☒	A description of any assumptions or corrections, such as tests of normality and adjustment for multiple comparisons
☐	☒	A full description of the statistical parameters including central tendency (e.g. means) or other basic estimates (e.g. regression coefficient) AND variation (e.g. standard deviation) or associated estimates of uncertainty (e.g. confidence intervals)
☐	☒	For null hypothesis testing, the test statistic (e.g. *F*, *t*, *r*) with confidence intervals, effect sizes, degrees of freedom and *P* value noted<br>*Give P values as exact values whenever suitable.*
☒	☐	For Bayesian analysis, information on the choice of priors and Markov chain Monte Carlo settings
☒	☐	For hierarchical and complex designs, identification of the appropriate level for tests and full reporting of outcomes
☒	☐	Estimates of effect sizes (e.g. Cohen's *d*, Pearson's *r*), indicating how they were calculated

Our web collection on statistics for biologists contains articles on many of the points above.

Software and code

Policy information about availability of computer code

Data collection	FACSDiva v8, FacsDiva v7.10, ZEN 2.3 software, Biacore T100 control software
Data analysis	GraphPad Prism v. 7.0; eBioscience analysis software; BIAevaluation software, FlowJo, v9.2, Flowjo v10.4.2, Image J

For manuscripts utilizing custom algorithms or software that are central to the research but not yet described in published literature, software must be made available to editors and reviewers. We strongly encourage code deposition in a community repository (e.g. GitHub). See the Nature Research guidelines for submitting code & software for further information.

Data

Policy information about availability of data

All manuscripts must include a data availability statement. This statement should provide the following information, where applicable:
- Accession codes, unique identifiers, or web links for publicly available datasets
- A list of figures that have associated raw data
- A description of any restrictions on data availability

The data supporting the findings of this study are available within the article and its supplementary Information files and from the corresponding authors upon reasonable request.

Field-specific reporting

Please select the one below that is the best fit for your research. If you are not sure, read the appropriate sections before making your selection.

☒ Life sciences ☐ Behavioural & social sciences ☐ Ecological, evolutionary & environmental sciences

For a reference copy of the document with all sections, see nature.com/documents/nr-reporting-summary-flat.pdf

Life sciences study design

All studies must disclose on these points even when the disclosure is negative.

Sample size	No statistical test was used to determined sample size. Instead sample size was determined empirically according to previous knowledge of the variation in experimental setup
Data exclusions	No data were excluded from the analyses
Replication	Only data that we were able to replicate at least in two independent experiments were included in the manuscript
Randomization	For experiments shown in Figure 4b-e, Fig 4f-g and Fig 5 Animals were randomly assigned to treatment groups as described in methods. Humanized Mice were randomized with maximum achievable p-value based on Donor and tumor volume.
Blinding	We did not perform a blinded assessment

Behavioural & social sciences study design

All studies must disclose on these points even when the disclosure is negative.

Study description	*Briefly describe the study type including whether data are quantitative, qualitative, or mixed-methods (e.g. qualitative cross-sectional, quantitative experimental, mixed-methods case study).*
Research sample	*State the research sample (e.g. Harvard university undergraduates, villagers in rural India) and provide relevant demographic information (e.g. age, sex) and indicate whether the sample is representative. Provide a rationale for the study sample chosen. For studies involving existing datasets, please describe the dataset and source.*
Sampling strategy	*Describe the sampling procedure (e.g. random, snowball, stratified, convenience). Describe the statistical methods that were used to predetermine sample size OR if no sample-size calculation was performed, describe how sample sizes were chosen and provide a rationale for why these sample sizes are sufficient. For qualitative data, please indicate whether data saturation was considered, and what criteria were used to decide that no further sampling was needed.*
Data collection	*Provide details about the data collection procedure, including the instruments or devices used to record the data (e.g. pen and paper, computer, eye tracker, video or audio equipment) whether anyone was present besides the participant(s) and the researcher, and whether the researcher was blind to experimental condition and/or the study hypothesis during data collection.*
Timing	*Indicate the start and stop dates of data collection. If there is a gap between collection periods, state the dates for each sample cohort.*
Data exclusions	*If no data were excluded from the analyses, state so OR if data were excluded, provide the exact number of exclusions and the rationale behind them, indicating whether exclusion criteria were pre-established.*
Non-participation	*State how many participants dropped out/declined participation and the reason(s) given OR provide response rate OR state that no participants dropped out/declined participation.*
Randomization	*If participants were not allocated into experimental groups, state so OR describe how participants were allocated to groups, and if allocation was not random, describe how covariates were controlled.*

Ecological, evolutionary & environmental sciences study design

All studies must disclose on these points even when the disclosure is negative.

Study description	*Briefly describe the study. For quantitative data include treatment factors and interactions, design structure (e.g. factorial, nested, hierarchical), nature and number of experimental units and replicates.*
Research sample	*Describe the research sample (e.g. a group of tagged Passer domesticus, all Stenocereus thurberi within Organ Pipe Cactus National Monument), and provide a rationale for the sample choice. When relevant, describe the organism taxa, source, sex, age range and*

any manipulations. State what population the sample is meant to represent when applicable. For studies involving existing datasets, describe the data and its source.

Sampling strategy	Note the sampling procedure. Describe the statistical methods that were used to predetermine sample size OR if no sample-size calculation was performed, describe how sample sizes were chosen and provide a rationale for why these sample sizes are sufficient.
Data collection	Describe the data collection procedure, including who recorded the data and how.
Timing and spatial scale	Indicate the start and stop dates of data collection, noting the frequency and periodicity of sampling and providing a rationale for these choices. If there is a gap between collection periods, state the dates for each sample cohort. Specify the spatial scale from which the data are taken
Data exclusions	If no data were excluded from the analyses, state so OR if data were excluded, describe the exclusions and the rationale behind them, indicating whether exclusion criteria were pre-established.
Reproducibility	Describe the measures taken to verify the reproducibility of experimental findings. For each experiment, note whether any attempts to repeat the experiment failed OR state that all attempts to repeat the experiment were successful.
Randomization	Describe how samples/organisms/participants were allocated into groups. If allocation was not random, describe how covariates were controlled. If this is not relevant to your study, explain why.
Blinding	Describe the extent of blinding used during data acquisition and analysis. If blinding was not possible, describe why OR explain why blinding was not relevant to your study.

Did the study involve field work? ☐ Yes ☐ No

Field work, collection and transport

Field conditions	Describe the study conditions for field work, providing relevant parameters (e.g. temperature, rainfall).
Location	State the location of the sampling or experiment, providing relevant parameters (e.g. latitude and longitude, elevation, water depth).
Access & import/export	Describe the efforts you have made to access habitats and to collect and import/export your samples in a responsible manner and in compliance with local, national and international laws, noting any permits that were obtained (give the name of the issuing authority, the date of issue, and any identifying information).
Disturbance	Describe any disturbance caused by the study and how it was minimized.

Reporting for specific materials, systems and methods

We require information from authors about some types of materials, experimental systems and methods used in many studies. Here, indicate whether each material, system or method listed is relevant to your study. If you are not sure if a list item applies to your research, read the appropriate section before selecting a response.

Materials & experimental systems

n/a	Involved in the study	
☐	☒	Antibodies
☐	☒	Eukaryotic cell lines
☒	☐	Palaeontology and archaeology
☐	☒	Animals and other organisms
☐	☒	Human research participants
☒	☐	Clinical data
☒	☐	Dual use research of concern

Methods

n/a	Involved in the study	
☒	☐	ChIP-seq
☐	☒	Flow cytometry
☒	☐	MRI-based neuroimaging

Antibodies

Antibodies used

anti-huCD3 OKT3; cat# 16-0037-85; #4303589 dilution 1: 4000
anti-huPD-L1 MIH1;14-5983-82; E04651-1630 dilution 1:500
anti-huCD137 M127; cat# 552532 ;#7054673 dilution 1:500
anti-huCD137 BBK2; cat# MS-621; SI2448351L dilution 1:200
Mouse IgG1, k isotype control antibody MOPC-21; cat# 401408; B189676 dilution 1:100
anti-huPD-L1 E1L3N; cat#1368; dilution 1: 200
Rabbit control antibodyRabbit (DA1E) mAb IgG XP® Isotype Control dilution 1:2500
biotin-conjugated anti-huCD137L BAF2295; UQI0116071 dilution 1:50
FITC anti-huCD8HIT8a; cat#555634; 524298 dilution 1:60
anti-huCD45ROUCLH1; cat#555493; 6320585 dilution 1:100
APC-H7 anti-huCD45RAHI100; cat#560674; 7047723 dilution 1:50
APC/Fire™ 750 anti-huCD62LDREG-56; cat#304846; B222694 dilution 1:100

PE anti-huCCR7150503; cat#FAB197P; LEV1415041 dilution 1:100
PE anti-huCD57TB01; cat#12-0577-42; 4292328 dilution 1:100
APC-H7 anti-huCD28CD28.2; cat#561368; 3031774 dilution 1:50
PE anti-huCD27M-T271; cat#555441; 7087984 dilution 1:100
AF488 anti-huCD56B159: cat#561905; 6057866 dilution 1:60
APC-H7 anti-huCD3SK7; cat#560176; 5255854 dilution 1:60
anti-huCD8SK1; cat#345784; 5335947dilution 1:100
anti-huCD137 4B4-1; cat#555955;dilution 1:100
anti-huPD-L1 130021; cat# 1561;dilution 1:100
anti-huCD3 OKT3; cat# 317315 dilution 1:1000
anti-huCD28 28.2; cat# 302923 dilution 1:1000
BV421 anti-huCD5UCHT2; cat#562646; 6049748 dilution 1:20
PerCP/Cy5.5 anti-huCD4 RPA-T4; cat#560650; 6209689 dilution 1:60
PerCP/Cy5.5 anti-huCD20 2H7; cat#560736; 6105587dilution 1:20
APC-H7 anti-huCD19HIB19 ; cat#560727; 5281893 dilution 1:20
PerCP/Cy5.5 anti-huCD16 3G8; cat#560717;6168980 dilution 1:60
APC-H7 anti-huCD14MΦP9; cat#560180; 526191 6dilution 1:60
FITC anti-huCD11cB-ly6; cat#561355;5128648 dilution 1:60
PerCP/Cy5.5 anti-huCD1237G3; cat#560904; 6070695 dilution 1:20
PE anti-huPD-L1MIH1; cat#557924; 5295791 dilution 1:20
PE Mouse IgG1,k isotype control antibodyMOPC-21; cat#556650; 3042677 dilution 1:20
BV421 anti-HLA-DRG46-6; cat#562805; 6077504 dilution 1:60
APC anti-huCD1374B4-1; cat#550890; 5225539 dilution 1:20
APC Mouse IgG1,k isotype control antibodyMOPC-21; cat#400122; B210935 dilution 1:20
AF647 anti-huCD1374B4-1; cat# 309823 dilution 1:50
AF488 anti-huCD8 RPA-T8; cat# 557696 dilution 1:100
human IgG4 isotype control antibody QA16Al5, Part No. 94242; 8261576 dilution 1:100
BV510 anti-huCD226 11A8; cat# 338330; B259831 dilution 1:40
Super Bright 436 anti-ICOS ISA-3; cat# 62-9948-42; 432838 dilution 1:160
PE anti-CTLA-4 BNI3; cat# 555853; 7208849 dilution 1:40
BV605 anti-CD137 4b4-1; cat# 745256; 8292716 dilution 1:40
AF647 anti-OX40 ACT35; cat# 350018; B245379 dilution 1:160
PerCP Cyanine5.5 anti-Lag-3; 11C3C65; cat# 369312; B254045 dilution 1:80
BV650 anti-Tim-3 7D3; cat# 565564; 8120836 dilution 1:80
AF488 anti-IL-10 JES3-9D7; cat#501413; B189479 dilution 1: 10
BV786 anti-GITR V27-580; cat# 747661; 8292722 dilution 1:40
APC-R700 anti huPD-L1; MIH1; cat#565188; 8092804 dilution 1:400
BUV737 anti-huCD3;UCHT1; cat# 564307; 8101534 dilution 1:20
APC-Cy7 anti-huCD8 SK1; cat# 557834; 8184768 dilution 1:20
PE-Cy7 anti-huCD4; SK3; cat# 557852; 8317967 dilution 1:20
AF647 anti-huCCR7; 3D12; cat# 557734; 8194595 dilution 1:20
BUV737 anti-huCD11b M1/70; cat # 564443; 7338572 dilution 1:20
PE anti-huPD-L129E.2A3; cat # 329706; B262098 dilution 1:20
BV 711™ anti-hu CD45RAHI100; cat# 304138; B250615 dilution 1:20
FITC anti-huCD45 HI30; cat# 555483 dilution 1:20
anti-Vβ13.1 TCR chain REA560; cat# 130-108-742 dilution 1:20
anti-huCD137 monoclonal antibody (utumilumab analog)
anti-huCD137 monoclonal antibody (urelumab analog)
anti-huPDL1 monoclonal antibody (atezolizumab analog)
anti-huPDL1 monoclonal antibody (atezolizumab)
anti-huPD-1 monoclonal (pembrolizumab)

Validation

anti-huCD3: Scramblase TMEM16F terminates T cell receptor signaling to restrict T cell exhaustion. Hu Y, et al. 2016. J Exp Med. 213: 2759 - 2772.
anti-huPD-L1: Amplification of N-Myc is associated with a T-cell-poor microenvironment in metastatic neuroblastoma restraining interferon pathway activity and chemokine expression. Layer J., Oncoimmunology . 2017 Apr 28;6(6)
anti-huCD137: Monoclonal antibodies against the 4-1BB T-cell activation molecule eradicate established tumors. Melero I, et al. Nat Med. 1997; 3(6):682-685
anti-huCD137 : 4-1BB is expressed on CD45RAhiROhi transitional T cell in human. Cell Immunol. Garni-Wagner BA, et. Al. 1996 169(1):91-8
Mouse IgG1, k isotype control antibody: Trend of telomerase activity change during human iPSC self-renewal and differentiation revealed by a quartz crystal microbalance based assay. Zhou Y, et al. Scientific reports 2014
anti-huPD-L1: Regulation of PD-L1 expression in K-ras-driven cancers through ROS-mediated FGFR1 signaling. Glorieux, C. et al. Redox Biol. 2021 Jan;38:101780.
"Rabbit control antibody:
Regulation of senescence escape by TSP1 and CD47 following chemotherapy treatment. Guillon, J et al. Cell Death Dis 2019 10(3):199"
biotin-conjugated anti-huCD137L: Crystal structure of the human 4-1BB/4-1BBL complex. RN Gilbreth, VY Oganesyan, H Amdouni, S Novarra, L Grinberg, A Barnes, M Baca.nJ Biol Chem. 2018 Jun 22;293(25):9880-9891
FITC anti-huCD8: An Isolated TCR alphabeta Restricted by HLA-A*02:01/CT37 Peptide Redirecting CD8+ T Cells To Kill and Secrete

IFN-gamma in Response to Lung Adenocarcinoma Cell Lines. Flores-Villanueva PO, Ganachari M, Guio H, Mejia JA, Granados J. J Immunol. 2018; 200(8):2965-2977.
anti-huCD45RO: Functional subsets of human helper-inducer cells defined by a new monoclonal antibody, UCHL1. Smith SH, Brown MH, Rowe D, Callard RE, Beverley PC. Immunology. 1986; 58(1):63-70.
APC-H7 anti-huCD45RA: Differential regulation of the peripheral lymph node homing receptor L-selectin on T cells during the virgin to memory cell transition.Picker LJ, Treer JR, Ferguson-Darnell B, Collins PA, Buck D, Terstappen LW. Control of lymphocyte recirculation in man. I. J Immunol. 1993; 150(3):1105-1121
APC/Fire™ 750 anti-huCD62L: Activation of an immunoregulatory and antiviral gene expression program in poly(I:C)-transfected human neutrophils. Tamassia N, et al. 2008. J. Immunol. 181:6563
PE anti-huCCR7: Pertussis circulation has increased T-cell immunity during childhood more than a second acellular booster vaccination in Dutch children 9 years of age. Schure R, de Rond L, Ozturk K, Hendrikx L, Sanders E, Berbers G, Buisman A PLoS ONE, 2102;7(7):e41928.
PE anti-huCD57: NKG2C(+)CD57(+) Natural Killer Cell Expansion Parallels Cytomegalovirus-Specific CD8(+) T Cell Evolution towards Senescence.Heath J,Newhook N,Comeau E,Gallant M,Fudge N,Grant M. I. J. Immunol. 2016:7470124
APC-H7 anti-huCD28: Verwilghen J, Vandenberghe P, Wallays G, et al. Simultaneous ligation of CD5 and CD28 on resting T lymphocytes induces T cell activation in the absence of T cell receptor/CD3 occupancy. J Immunol. 1993; 150(3):835-846.
PE anti-huCD27: A modulating disulfide-linked T cell activation antigen. Bigler RD, Bushkin Y, Chiorazzi N. S152 (CD27). J Immunol. 1988; 141(1):21-28. (Biology: Flow cytometry).
AF488 anti-huCD56: Schlossman SF. Stuart F. Schlossman .. et al., ed. Leucocyte typing V : white cell differentiation antigens : proceedings of the fifth international workshop and conference held in Boston, USA, 3-7 November, 1993. Oxford: Oxford University Press; 1995
APC-H7 anti-huCD3: Human T lymphocyte proliferation induced by a pan-T monoclonal antibody (anti-Leu 4): heterogeneity of response is a function of monocytes. Kaneoka H, Perez-Rojas G, Sasasuki T, Benike CJ, Engleman EG. J Immunol. 1983; 131(1):158-164
anti-huCD8: Function of multiple sclerosis-protective HLA class I alleles revealed by genome-wide protein-quantitative trait loci mapping of interferon signalling. Lundtoft C., et al 2020 Plos Genetics
anti-huCD137: 4-1BB is expressed on CD45RAhiROhi transitional T cell in humans. Garni-Wagner BA, Lee ZH, Kim YJ, Wilde C, Kang CY, Kwon BS. Cell Immunol. 1996; 169(1):91-98
anti-huPD-L1: IFN-gamma generates maturation-arrested dendritic cells that induce T cell hyporesponsiveness independent of Foxp3 + T-regulatory cell generation. Rojas D, Krishnan R, Immunol. Lett., 2010;132(1):31-7.
anti-huCD3: Scramblase TMEM16F terminates T cell receptor signaling to restrict T cell exhaustion. Hu Y, et al. 2016. J Exp Med. 213: 2759 - 2772.
anti-huCD28: Quantitative Interactomics in Primary T Cells Provides a Rationale for Concomitant PD-1 and BTLA Coinhibitor Blockade in Cancer Immunotherapy. elis-Gutierrez J et al. 2019. Cell Rep. 27(11):3315-3330
BV421 anti-huCD5: CD5 is associated with the human B cell antigen receptor complex. Lankester AC, van Schijndel GM, Cordell JL, van Noesel CJ, van Lier RA. Eur J Immunol. 1994; 24(4):812-816.
PerCP/Cy5.5 anti-huCD4: Schlossman SF et al., ed. Leucocyte typing V : white cell differentiation antigens : proceedings of the fifth international workshop and conference held in Boston, USA, 3-7 November, 1993. Oxford: Oxford University Press; 1995; .
PerCP/Cy5.5 anti-huCD20: CD20 (pan-B cell) antigen is expressed at a low level on a subpopulation of human T lymphocytes. Hultin LE, Hausner MA, Hultin PM, Giorgi JV. Cytometry. 1993; 14(2):193-204
APC-H7 anti-huCD19: The CD19 signal transduction complex of B lymphocytes. Deletion of the CD19 cytoplasmic domain alters signal transduction but not complex formation with TAPA-1 and Leu 13.Bradbury LE, Goldmacher VS, Tedder TF. J Immunol. 1993; 151(6):2915-2927
PerCP/Cy5.5 anti-huCD16: Human neutrophil Fc gamma receptor distribution and structure. Fleit HB, Wright SD, Unkeless JC. Proc Natl Acad Sci U S A. 1982; 79(10):3275-3279.
APC-H7 anti-huCD14: Human mononuclear phagocyte differentiation antigens. I. Patterns of antigenic expression on the surface of human monocytes and macrophages defined by monoclonal antibodies.Dimitriu-Bona A, Burmester GR, Waters SJ, Winchester RJ. J Immunol. 1983; 130(1):145-152.
FITC anti-huCD11c: Leucocyte typing IV : white cell differentiation antigens. Knapp W. et al., ed. Oxford New York: Oxford University Press; 1989; :1-1182.
PerCP/Cy5.5 anti-huCD123: Sun Q, et al. Monoclonal antibody 7G3 recognizes the N-terminal domain of the human interleukin-3 (IL-3) receptor alpha-chain and functions as a specific IL-3 receptor antagonist.. Blood. 1996; 87(1):83-92.
PE anti-huPD-L1: Carter L, et al. PD-1:PD-L inhibitory pathway affects both CD4(+) and CD8(+) T cells and is overcome by IL-2. Eur J Immunol. 2002; 32:634-643
PE Mouse IgG1,k isotype control antibody:
BV421 anti-HLA-DR: Sorg RV, et al. Identification of cord blood dendritic cells as an immature CD11c- population. Blood. 1999; 93(7):2302-2307
APC anti-huCD137: 4-1BB is expressed on CD45RAhiROhi transitional T cell in humans. Garni-Wagner BA, Lee ZH, Kim YJ, Wilde C, Kang CY, Kwon BS. Cell Immunol. 1996; 169(1):91-98
APC Mouse IgG1,k isotype control antibody:
AF647 anti-huCD137: Kinetic study of interleukin-2 binding on the reconstituted interleukin-2 receptor complexes including the human gamma chain. Matsuoka M, Takeshita T, Ishii N, Nakamura M, Ohkubo T, Sugamura K. Eur J Immunol. 1993; 23(10):2472-2476
AF488 anti-huCD8: Leucocyte typing V : white cell differentiation antigens : proceedings of the fifth international workshop and conference held in Boston, USA, 3-7 November, 1993. Schlossman SF. Stuart F. Schlossman .. et al., ed. . Oxford: Oxford University Press; 1995
human IgG4 isotype control antibody:
BV510 anti-huCD226: Cutting edge: CD96 (tactile) promotes NK cell-target cell adhesion by interacting with the poliovirus receptor (CD155)Fuchs A, et al. 2004. J. Immunol. 172:3994.
Super Bright 436 anti-ICOS: The adjuvant GLA-SE promotes human Tfh cell expansion and emergence of public TCRβ clonotypes. Hill DL, Pierson W, Bolland DJ, Mkindi C, Carr EJ, Wang J, Houard S, Wingett SW, Audran R, Wallin EF, Jongo SA, Kamaka K, Zand M, Spertini F, Daubenberger C, Corcoran AE, Linterman MA. J Exp Med. 2019 Aug 5;216(8):1857-1873
PE anti-CTLA-4: Activated T cells can induce high levels of CTLA-4 expression on B cells.Kuiper HM, Brouwer M, Linsley PS, van Lier RA. J Immunol. 1995; 155(4):1776-1783.
BV605 anti-CD137: 4-1BB is expressed on CD45RAhiROhi transitional T cell in humans. Garni-Wagner BA, Lee ZH, Kim YJ, Wilde C, Kang CY, Kwon BS. Cell Immunol. 1996; 169(1):91-98.
AF647 anti-OX40: Anti-apoptotic Protein BIRC5 Maintains Survival of HIV-1-Infected CD4 + T Cells. Kuo HH, et al. 2018. Immunity.

48:1183.
PerCP Cyanine5.5 anti-Lag-3: PD-1 silencing impairs the anti-tumor function of chimeric antigen receptor modified T cells by inhibiting proliferation activity. Wei J, et al. 2019. J Immunother Cancer. 7:209
BV650 anti-Tim-3: Tim-3 inhibits T helper type 1-mediated auto- and alloimmune responses and promotes immunological tolerance. Domenig C, Zheng XX, Sabatos CA, et al. Nat Immunol. 2003; 4(11):1093-1101.
AF488 anti-IL-10: Dimethyl fumarate induces changes in B- and T-lymphocyte function independent of effects on absolute lymphocyte count. Longbrake EE, et al. 2018. Mult Scler. 24:728.
BV786 anti-GITR: Cell-specific and context-dependent effects of GITR in cancer, autoimmunity, and infection. Clouthier DL, Watts TH. . Cytokine Growth Factor Rev. 2014; 25(2):91-106.
APC-R700 anti huPD-L1: Blockade of programmed death-1 ligand on dendritic cells enhances T cell activation and cytokine production. Brown JA, Dorfman DM, Ma FR, et al. J Immunol. 2003; 170:1257-1266
BUV737 anti-huCD3: CD3 complex.'Ernst DN, Shih CC. CD3 complex. J Biol Regul Homeost Agents. 2000; 14(3):226-229.
APC-Cy7 anti-huCD8: Activation of human T lymphocyte subsets: helper and suppressor/cytotoxic T cells recognize and respond to distinct histocompatibility antigens. Engleman EG, Benike CJ, Grumet FC, Evans RL. J Immunol. 1981; 127(5):2124-2129
PE-Cy7 anti-huCD4: Leucocyte typing IV : white cell differentiation antigens. Knapp W. et al., ed. Oxford New York: Oxford University Press; 1989; :1-1182.'Cy7PE and Cy7APC: bright new probes for immunofluorescence. Roederer M, Kantor AB, Parks DR, Herzenberg LA. Cytometry. 1996; 24(3):191-197.
AF647 anti-huCCR7: CCR7 and its ligands: balancing immunity and tolerance. Forster R, Davalos-Misslitz AC, Rot A. Nat Rev Immunol. 2008; 8(5):362-371
BUV737 anti-huCD11b: Competition between lymphocyte function-associated antigen 1 (CD11a/CD18) and Mac-1 (CD11b/CD18) for binding to intercellular adhesion molecule-1 (CD54). Lub M, van Kooyk Y, Figdor CG. J Leukoc Biol. 1996; 59(5):648-655
PE anti-huPD-L1: PD-L1 antibodies to its cytoplasmic domain most clearly delineate cell membranes in immunohistochemical staining of tumor cells. Mahoney KM, et al. 2015. Cancer Immunol. Res. 3:1308
BV 711™ anti-hu CD45RA: Abd Hamid M, et al. 2020. Cancer Immunology Research. 8(2):203-216.
FITC anti-huCD45: Mubritinib Targets the Electron Transport Chain Complex I and Reveals the Landscape of OXPHOS Dependency in Acute Myeloid Leukemia. Baccelli I, et al. 2020. Cancer Cell. 36(1):84-99
anti-Vβ13.1 TCR chain: TCR v(beta) usage and clonality of T cells isolated from progressing and rejected tumor sites before and after in vitro cultur. 'Kurt, R. A. et al. (2000) e. Int. Immunol. 12(5): 639-646
anti-huCD137 monoclonal antibody (utumilumab analog): WO2015119923 4-1BB binding molecules
anti-huCD137 monoclonal antibody (urelumab analog): WO 2005/035584 FULLY HUMAN ANTIBODIES AGAINST HUMAN 4-1BB
anti-huPDL1 monoclonal antibody (atezolizumab analog): WO 2010/077634 ANTI-PD-L1 ANTIBODIES AND THEIR USE TO ENHANCE T-CELL FUNCTION
anti-huPDL1 monoclonal antibody (atezolizumab): Roche
anti-huPD-1 monoclonal (pembrolizumab): Merck

Eukaryotic cell lines

Policy information about cell lines

Cell line source(s)

Human ES-2	ATCC	cat# CRL-1978 RRID:CVCL_CZ94
Human Freestyle 293F	Invitrogen	cat#R790-07 RRID: CVCL_D603
Human MDA-MB-231 cells	ATCC	cat# CRM-HTB-26 RRID:CVCL_0062
Human BxPC-3	ATCC	cat#CRL-1687; RRID:CVCL_0186
Human HEK293T ATCC cat-CRL-11268 RRID:CVCL_1926		
Human A549-A2-ESO Moon et al, Clin Cancer Res. 2016 Jan 15;22(2):436–47		
Human Jurkat E6.1	ATCC	cat# TIB-152; RRID:CVCL_0367
Human Jurkat CD137-NFκB-luc	This paper	
Human Jurkat CD137 clone K	This paper	
Human A549-A2-ESO-1-PD-L1-hi	This paper	
Human A549-A2-ESO-1-PD-L1-ko	This paper	
Hamster: CHO-K1	DSMZ	cat#ACC-110;RRID:CVCL_0214
Hamster: CHO-PD-L1	This paper	
Hamster: CHO-CD137	This paper	

Authentication

No cell line authentication was performed

Mycoplasma contamination

All cell lines were tested negative for mycoplasma.

Commonly misidentified lines (See ICLAC register)

No commonly misidentified cell lines were used (according to ICLAC register version 10)

Palaeontology and Archaeology

Specimen provenance

N/A

Specimen deposition

N/A

Dating methods | N/A

☐ Tick this box to confirm that the raw and calibrated dates are available in the paper or in Supplementary Information.

Ethics oversight | N/A

Note that full information on the approval of the study protocol must also be provided in the manuscript.

Animals and other organisms

Policy information about studies involving animals; ARRIVE guidelines recommended for reporting animal research

Laboratory animals	laboratory animals; All mice were group-housed (9 mice per cage) and maintained under a regular light-dark cycle altered every 12 hours with free access to water and is LabDiet - PMI NIH rat and mouse autoclavable 6% fat (as used by Jackson laboratory). Along with hydrogel and dietgel (distributed by Clear H2O). Female huCD34+ NSG mice 12 weeks post CD34 engraftment, were obtained from the Jackson Laboratory, Bar Harbor, ME and were 27-28 weeks of age at the time of MDA-MB231 study initiation study. Ly95-NY-ESO model, NSG mice Female were 6-8 weeks of age at study initiation.<br>NHP studies: Macaca fascicularis Male and female Animals were 115 and 169 weeks old and weighed between 2.06 to 4.04 kg at the start of dosing.
Wild animals	No wild animals were used in the study
Field-collected samples	No field collected samples were used in the study
Ethics oversight	The utilization of animals for the described studies was approved by University of Pennsylvania's IACUC (Animal Welfare Assurance #A3079-01 / Protocol # 804000). All experiments were performed in accordance with the committee's guidelines and regulations. All authors have complied with the guidelines of Animal Research: Reporting of In Vivo Experiments; Cynomolgus studies were conducted in accordance with the requirements of the Animals (Scientific Procedures) Act 1986 (UK), and a local ethical review was maintained.

Note that full information on the approval of the study protocol must also be provided in the manuscript.

Human research participants

Policy information about studies involving human research participants

Population characteristics	Blood samples were derived from healthy donors. Endometrial tumor samples were obtained from Avaden Biosciences (Seattle WA). Every sample was actively consented in an IRB approved research protocol at a US-based CLIA/CAP accredited laboratory.
Recruitment	Healthy donors were recruited by Sanquin Blood Supply (Amsterdam, the Netherlands) and BioIVT (Westbury, NY).
Ethics oversight	Sanquin:all donors provided written informed consent in accordance with the Declaration of Helsinki and the protocol of the local institutional review board, the Medical Ethics Committee of Sanquin Blood Supply. BioIVT: all informed consent were documented in writing from each individual leukopak donor per the requirements of the Department of Health and Human Services regulations for the protection of human subjects (45 CFR §46.116 and §46.117) and Good Clinical Practice (GLP), (ICH E6).

Note that full information on the approval of the study protocol must also be provided in the manuscript.

Clinical data

Policy information about clinical studies

All manuscripts should comply with the ICMJE guidelines for publication of clinical research and a completed CONSORT checklist must be included with all submissions.

Clinical trial registration	N/A
Study protocol	N/A
Data collection	N/A
Outcomes	N/A

Dual use research of concern

Policy information about dual use research of concern

Hazards

Could the accidental, deliberate or reckless misuse of agents or technologies generated in the work, or the application of information presented in the manuscript, pose a threat to:

No	Yes	
☒	☐	Public health
☒	☐	National security
☒	☐	Crops and/or livestock
☒	☐	Ecosystems
☒	☐	Any other significant area

Experiments of concern

Does the work involve any of these experiments of concern:

No	Yes	
☒	☐	Demonstrate how to render a vaccine ineffective
☒	☐	Confer resistance to therapeutically useful antibiotics or antiviral agents
☒	☐	Enhance the virulence of a pathogen or render a nonpathogen virulent
☒	☐	Increase transmissibility of a pathogen
☒	☐	Alter the host range of a pathogen
☒	☐	Enable evasion of diagnostic/detection modalities
☒	☐	Enable the weaponization of a biological agent or toxin
☒	☐	Any other potentially harmful combination of experiments and agents

ChIP-seq

Data deposition

☐ Confirm that both raw and final processed data have been deposited in a public database such as GEO.

☐ Confirm that you have deposited or provided access to graph files (e.g. BED files) for the called peaks.

Data access links<br>*May remain private before publication.*	N/A
Files in database submission	N/A
Genome browser session<br>(e.g. UCSC)	N/A

Methodology

Replicates	N/A
Sequencing depth	N/A
Antibodies	N/A
Peak calling parameters	N/A
Data quality	N/A
Software	N/A

Flow Cytometry

Plots

Confirm that:

☒ The axis labels state the marker and fluorochrome used (e.g. CD4-FITC).

☒ The axis scales are clearly visible. Include numbers along axes only for bottom left plot of group (a 'group' is an analysis of identical markers).

☐ All plots are contour plots with outliers or pseudocolor plots.

☒ A numerical value for number of cells or percentage (with statistics) is provided.

Methodology

Sample preparation	For sample preparation see Supplementary Information "Human Blood Samples", "Cell lines" and "T cell transactivation assay
Instrument	Cells were analyzed on a FACSCanto Flow cytometer (BD, ref. no. 337175) or a FACSFortessa Flow cytometer (BD; ref no. 67465)
Software	FlowJo, v9.2, Flowjo v10.4.2
Cell population abundance	Figure S1A: T cell purity after isolation at the start of assay > 95%<br>Figure S1C: average abundance (% of total PBMC): CD3+CD4+ (33%), CD3+CD8+ (18%), CD19+CD20+ (7%), CD56+CD3-CD14-CD19-(12%), CD14+HLADR+ (12%), HLA-DR+CD3-CD14-CD19-CD11c-CD123+ (0.1%) and HLA-DR+CD3-CD14-CD19-CD11c +CD123- (1%)<br>Figure S2B, S4B: Cell line purity 100%<br>Figure S4A: T cells account for 3 - 24 % of total cell population within endometrial tumor samples (donor dependent); T cell subpopulations as % of T cells: Treg (6 - 18%), CD4+ (33 - 69%) and CD8+ (16 - 53%)<br>Figure S4G: huCD45+ cells account for approximately 12% of live cells within tumor sample, of which 0.3 - 60% T cells<br>Figure S4C: Ly95 cells account for approximately 34% of the CD3+ TIL population<br>Figure S7: abundance (% of lymphocyte population, average) CD3 = 55%, CD4 = 30%, CD8 = 18%, CD20 = 23%, CD16 = 17%
Gating strategy	Figure S1A, S2B and S4B and S4C: FSC/SSC plot was used to gate cells and exclude debris; histograms were plotted of relevant fluorescent channels (PE, APC) for the cells gated in the FSC/SSC plot.<br>Figure S1C: Single cells were gated (FSC-A/FSC-H), FSC-A/SSC-A plot to exclude debris, FVD-e506/FSC-A to gate live cells, and PD-L1 expression analyzed on CD3+CD4+, CD3+CD8+, CD19+CD20+ (7%), CD56+CD3-CD14-CD19-, CD14+HLADR+, HLA-DR +CD3-CD14-CD19-CD11c-CD123+ and HLA-DR+CD3-CD14-CD19-CD11c+CD123- populations.<br>Figure S4A: Single cells were gated, CD45+ and CD45- cells to determine PD-L1 expression on both populations; CD45+ cell gate was used for gating live cells, followed by CD19-CD14- cells to gate T cells (CD3+). CD8+ were gated within T cell gate to identify CD8+ T cell population. CD4+ cells were gated within CD3+ population and CD4+ helper T cells identified by gating FoxP3-CD25- cells and Treg cells as CD25+FoxP3+ cells.<br>Figure S4G: FSC-A/SSC-A plot to exclude debris, Live/dead gate to gate live cells, huCD45+ to gate human hematopoeitic cells, CD3+ to gate human T cells within hematopoietic cells. CD11b+CD14+ cells within hematopoietic cells were gated to identify myeloid cells.<br>Figure S7: Lymphocytes were gated based on CD45+ and SSC and analyzed for CD3+, CD3+CD4+, CD3+CD8+, CD16+CD3- and CD20+ subpopulations.

☒ Tick this box to confirm that a figure exemplifying the gating strategy is provided in the Supplementary Information.

Magnetic resonance imaging

Experimental design

Design type	N/A
Design specifications	N/A
Behavioral performance measures	N/A

Acquisition

Imaging type(s)	N/A
Field strength	N/A
Sequence & imaging parameters	N/A
Area of acquisition	N/A

Diffusion MRI ☐ Used ☒ Not used

Preprocessing

Preprocessing software	N/A
Normalization	N/A
Normalization template	N/A
Noise and artifact removal	N/A
Volume censoring	N/A

Statistical modeling & inference

Model type and settings: N/A

Effect(s) tested: N/A

Specify type of analysis: ☐ Whole brain ☐ ROI-based ☐ Both

Statistic type for inference (See Eklund et al. 2016): N/A

Correction: N/A

Models & analysis

n/a	Involved in the study
☒	☐ Functional and/or effective connectivity
☒	☐ Graph analysis
☒	☐ Multivariate modeling or predictive analysis

Functional and/or effective connectivity: *Report the measures of dependence used and the model details (e.g. Pearson correlation, partial correlation, mutual information).*

Graph analysis: *Report the dependent variable and connectivity measure, specifying weighted graph or binarized graph, subject- or group-level, and the global and/or node summaries used (e.g. clustering coefficient, efficiency, etc.).*

Multivariate modeling and predictive analysis: *Specify independent variables, features extraction and dimension reduction, model, training and evaluation metrics.*
